# Fusion-positive rhabdomyosarcoma oncofusions share a common interactome

**DOI:** 10.1038/s41467-026-73749-y

**Published:** 2026-05-28

**Authors:** S. P. Zimmerman, C. D. Delaney, B. K. Lau, L. B. DeGraw, G. H. Rupprecht, M. J. A. Groot Koerkamp, T. de Souza, J. Drost, R. A. Schoot, M. T. Meister, J. F. Shern, L. M. Wagner, G. G. Wang, K. C. Wood, C. M. Linardic, C. M. Counter

**Affiliations:** 1 Department of Pharmacology & Cancer Biology, Duke University, Durham, NC USA; 2 UNC MD/PHD Program, University of North Carolina at Chapel Hill School of Medicine, Chapel Hill, NC USA; 3 Department of Medicine, Division of Medical Oncology, Duke University, Durham, NC USA; 4 Princess Máxima Center for Pediatric Oncology, Utrecht, The Netherlands; 5 Oncode Institute, Utrecht, The Netherlands; 6 Division Cell Biology, Metabolism & Cancer, Department Biomolecular Health Sciences, Faculty of Veterinary Medicine, Utrecht University, Utrecht, The Netherlands; 7 Pediatric Oncology Branch, Center for Cancer Research, National Cancer Institute, Bethesda, MD USA; 8 Department of Pediatrics, Division of Pediatric Hematology-Oncology, Duke University, Durham, NC USA

**Keywords:** Oncogenes, Paediatric cancer

## Abstract

Fusion-positive rhabdomyosarcoma (FP-RMS) arises from at least seven distinct oncofusions sharing a common PAX3/7 N-terminal DNA-binding domain fused to divergent C-terminal partners. How different oncofusions produce the same cancer was unknown. Here we show they are functionally interchangeable, associate with a shared protein network we term the common interactome, bind overlapping target genes, and drive a similar core transcriptional program. The common interactome contains the C-terminal partners of known oncofusions and a newly identified translocation, suggesting oncofusions arise by PAX3/7 DNA-binding domain fusing to interactome members. As loss of common interactome proteins impaired oncogenic activity we screened the interactome for shared vulnerabilities. This identified thymidylate synthase as preferentially required for FP-RMS growth. Accordingly, the antifolate pralatrexate suppressed growth across all seven oncofusions, in multiple human FP-RMS cell lines, and a patient-derived xenograft. These findings demonstrate that divergent FP-RMS oncofusions are functionally fungible through a shared interactome that defines common vulnerabilities.

## Abstract (web summary)

Fusion-positive rhabdomyosarcoma is driven by several distinct gene fusions that produce the same cancer type. Here, the authors show that these oncofusions share a common set of interacting proteins and gene targets that drive a shared transcriptional program and reveal shared therapeutic vulnerabilities, including sensitivity to antifolate treatment.

## Introduction

*R*habdo*m*yo*s*arcoma (RMS) is the most common pediatric soft-tissue sarcoma, comprised of two major histological subtypes, *e*mbryonal (eRMS) and *a*lveolar (aRMS). While most therapeutic progress has been made in eRMS^1^, the overall survival of aRMS has remained at ~30% for over the last 30 years^2,3^. aRMS is typically characterized by the reciprocal translocation t(2;13) that gives rise to a transcriptional oncofusion comprised of the N-terminal portion of the *pa*ired bo*x 3* (*PAX3)* transcription factor encoding DNA-binding domains fused in-frame to the C-terminal portion of the *fo*rkhead bo*x O1* (*FOXO1*) transcription factor encoding the transactivation domain^1^. This *PAX3::FOXO1* gene fusion, termed here as *P3F1* for ease of discussion, appears to be the initiating mutation. Namely, P3F1 supports bypass of the senescence checkpoint in primary human skeletal muscle myoblasts^4^ and is an obligate gene to genetically transform these cells into a tumorigenic state that resembles aRMS^5^. Activating *P3F1* during myogenesis also generates aRMS-like tumors in mice^6^. Conversely, inactivating endogenous *P3F1* in human aRMS cell lines leads to cell death and differentiation^7,8^. Since there are few cooperating genetic alterations detected in aRMS tumors^9^, understanding how this oncofusion arises would shed light on the origin of this devastating pediatric cancer.

The oncogenic activity of P3F1 can be ascribed to the DNA-binding activity of the PAX3 portion recruiting transcriptional machinery, via the transactivation domain of the FOXO1 portion, to aberrantly activate genes associated with aRMS^10^. Beyond this, the translocation itself marries DNA upstream of *PAX3* and downstream of *FOXO1*, which has been shown to deregulate expression of the resultant oncofusion gene^11^. The fusion also deletes portions of both PAX3 and FOXO1 that bind regulatory proteins, sites of post-translational modifications, and potentially even the DNA-binding specificity that together alter the activity of the remaining PAX3 and FOXO1 proteins^10,12,13^. Indeed, P3F1 generates a transcription pattern distinct from PAX3^14^, which favors cell proliferation and disfavors terminal differentiation^8,15,16^.

While most aRMS tumors contain the P3F1 oncofusion, termed fusion-positive RMS (FP-RMS), approximately 20% lack the characteristic t(2;13) translocation. However, a number of these cases harbor translocations comprised of the DNA-binding domain of PAX3 or its paralog PAX7 fused to FOXO1, FOXO4, NCOA1, NCOA2, MAML3, or INO80D, referred to as P3F4, P3N1, P3N2, P3M, P3I, and P7F1^9,17–21^. The seven translocations cluster into four groups based on homology. The first group, P3F1, P3F4, and P7F1, are derived from PAX and FOXO paralogues. *PAX3* and *PAX7* were likely produced by gene duplication^22^, share 93% and 97% sequence identity in the paired-box and homeobox DNA-binding domains, respectively^23^, and *PAX7* knock-in at the *PAX3* locus recapitulates most *PAX3* functions in mice^24^. FOXO1 and FOXO4 share homology in their N-terminal winged-helix and forkhead DNA-binding domains, with their C-terminal transactivation domains showing 56% identity, shared binding partners, and conserved post-translational modifications^25,26^. The second group, P3N1 and P3N2, involves NCOA1 and NCOA2. These nuclear receptor co-activators for steroid and hormone receptors share 54% identity and contain similar C-terminal transactivation domains^27^, which when fused to a DNA-binding domain, are sufficient to drive transcription^28^, a circumstance analogous to fusion with PAX3. The third group, P3M, involves MAML3, a transcriptional co-activator in Notch-mediated gene expression via its C-terminal transactivation domain^29^. The fourth group, P3I, involves INO80D, a subunit of the INO80 complex which contributes to chromatin remodeling and DNA repair^30,31^. Beyond being broadly involved in chromatin remodeling, transcription, and/or DNA repair, the C-terminus of these four groups share neither regions of obvious sequence identity nor a more defined common function. The mechanism by which fundamentally distinct oncofusions, sharing only a DNA-binding domain, drive the same cancer was unclear.

Here, we provide evidence that the different oncofusions arise from the same PAX3/7 DNA-binding domain fused to proteins within a common interactome to drive a shared oncogenic program, and consequently, harbor common vulnerabilities that can be therapeutically targeted.

## Results

### P3F1-TurboV5 is oncogenic

Protein-protein interactions are critical for the oncogenic activity of P3F1^10,32–34^. To gain insights into oncofusion function, we employed proximity labeling, wherein a protein-of-interest is fused to the engineered biotin ligase TurboID to biotinylate proteins within ~10 nm and thereby capturing weak or transient interactions in live cell^35–37^. Indeed, a prior version of full-length BirA* has been fused to P3F1 and over-expressed in the dual adenoviral- and SV40-transformed embryonic kidney cell line 293T^32^ and TurboID has been fused to oncofusions in other cancers^38^ to identify potential interacting proteins. Here, we applied this approach to human FP-RMS cells.

**Fig. 1:**
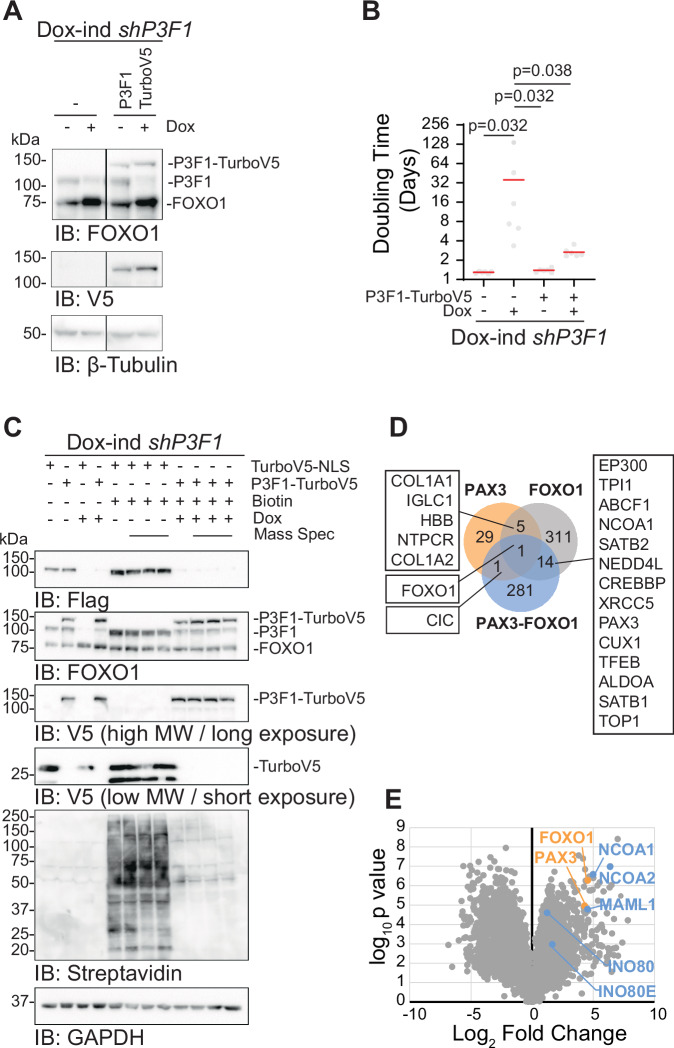
The P3F1 interactome contains the C-terminal fusion partners of other oncofusions. **A** Immunoblot (IB) with the indicated antibodies from RH4-flag cells transduced with shRNA-resistant P3F1-TurboV5 or vector control and treated with or without doxycycline (Dox) to induce *shP3F1* expression. Representative images of 2 replicate experiments. **B** Bar plot of the mean (red line) and individual doubling time (circles) of RH4-flag cells with indicated doxycycline induction of *shP3F1* and expression of P3F1-TurboV5. Values were calculated by fitting growth curves to data presented in Suppl. Fig. S1B. (*n* = 6 replicates over 2 independent experiments per sample. One-way ANOVA. *p < 0.05, red line represents mean). **C** Immunoblot analysis with the indicated antibodies from RH4-flag cells transduced with shRNA-resistant P3F1-TurboV5 or TurboV5-NLS and treated or without Dox to induce *shP3F1* expression and biotin to covalently label proteins. Indicated samples were sent for quantitative mass spectrometry. (*n* = 1 immunoblot of 4 technical replicates). **D** Venn diagram of proteins identified by proximity labeling by P3F1-TurboID (fold-change > 5 and *p* < 0.01; two-tailed heteroscedastic t-test; *n* = 3 technical replicates per sample) and previously identified PAX3 and FOXO1 interaction (BioGrid). **E** Volcano plot of fold-change values of normalized protein levels identified by proximity labeling between P3F1-TurboV5 and TurboV5-NLS versus -Log_10_ p value. (two-tailed heteroscedastic t-test; *n* = 3 technical replicates per sample). Source data are provided as a Source Data file.

To link proximity-labeled proteins to P3F1 function, we first validated that the fusion of P3F1 to the TurboID moiety did not interfere with its oncogenic activity. To avoid inactivating the wild-type *PAX3* and *FOXO1* alleles, which would confound results, we stably expressed doxycycline-inducible shRNA targeting the unique P3F1 breakpoint (*shP3F1*) in the human FP-RMS cell line RH4 in which the endogenous P3F1 was flag-epitope tagged (RH4-flag)^32^. In parallel, we also generated a lentiviral expression vector encoding an *shRNA*-resistant version of *P3F1* cDNA fused in-frame at the 3′ end to the *Turbo*ID moiety containing a *V5*-epitope tag for detection purposes (P3F1-TurboV5). *shP3F1* RH4-flag cells were then stably infected with a lentivirus encoding P3F1-TurboV5 or no transgene as a control and treated with or without doxycycline. Immunoblotting with anti-FOXO1 and -V5 antibodies confirmed ectopic P3F1-TurboV5 expression (at levels comparable to endogenous P3F1) was retained upon suppressing endogenous P3F1 via *shP3F1* (Fig. 1A).

We next compared cell growth in control and P3F1-TurboV5-expressing *shP3F1* RH4-flag cells, with or without doxycycline induction. Without doxycycline, both cell lines exhibited similar growth. Upon induction, cell numbers steadily declined, an effect blunted by P3F1-TurboV5 expression (Fig. 1B; Suppl. Fig. 1A, B). After an initial adjustment period, perhaps due to the previously observed selection for optimal protein expression^39^, ectopic P3F1-TurboV5 fully restored the doubling time to levels observed without doxycycline induction (*see* below). Thus, P3F1-TurboV5 retains oncogenic activity and is suitable for proximity labeling in FP-RMS cells.

### The P3F1 interactome contains fusion partners from other FP-RMS oncofusions

To determine the corresponding interactome, quadruplicate cultures of *shP3F1* RH4-flag cells expressing P3F1-TurboV5 were induced with doxycycline, such that proliferation was maintained by the ectopic oncofusion, followed by culture in biotin for proximity labeling, after which lysates were collected for immunoblotting and mass spectrometry. To control for non-specific biotin labeling, quadruplicate cultures of *shP3F1* RH4-flag cells were stably infected with a lentivirus expressing the TurboV5 moiety fused in-frame to a Nuclear Localization Sequence (TurboV5-NLS) and treated as above. Finally, four samples not exposed to biotin were collected to assess background biotin labeling, namely *shP3F1* RH4-flag cells expressing TurboV5-NLS or P3F1-TurboV5 with or without doxycycline induction. Immunoblotting with anti-FOXO1 and -flag antibodies confirmed reduced expression of endogenous P3F1 in all samples after doxycycline induction. Immunoblotting with an anti-V5 antibody and streptavidin confirmed ectopic expression as well as biotinylation of endogenous proteins by P3F1-TurboV5 and TurboV5-NLS. As expected, the latter was more highly expressed, presumably owing to its smaller size and hence more effectively biotinylated proteins (Fig. 1C). Given that P3F1-TurboV5 was validated to both maintain oncogenesis upon inactivation of the endogenous *P3F1* gene and biotinylated proteins, samples were subjected to streptavidin affinity-capture and biotinylated proteins identified by mass spectrometry, with one sample from each set subjected to qualitative analysis and three devoted to quantitatively measure protein abundance.

Normalized protein expression values were computed from peptide intensities for each sample and the mean value across all three replicates compared between P3F1-TurboV5 and TurboV5-NLS to determine a fold-change enrichment and *p*-value. Using a rigorous threshold of fold-change enrichment > 5 and *p* < 0.01, 297 proteins were identified (Suppl. Dataset 1), which we term the interactome for ease of description. Very few proteins have been identified as direct P3F1 interactors to benchmark the interactome. However, the BioGRID^40^ database of protein-protein interactions compiled 36 proteins that may interact with PAX3, two of which we detected, and 331 proteins that may interact with FOXO1, 15 of which we detected (Fig. 1D). This represents a statistical enrichment of proteins suggested to interact with PAX3 or FOXO1 (Fischer’s exact test; *p* = 0.00012). Additionally, roughly a quarter of the proteins we identified overlap with those proximity labeled by P3F1-BirA* when overexpressed in 293T cells^32^ (Suppl. Fig. 1C, D), with presumably the remaining three quarters reflecting the difference between human FP-RMS cancer cells and virally-transformed kidney cells. Nevertheless, this also represents a statistical enrichment of proteins previously identified by proximity labeling (Fischer’s exact test; *p* < 1 × 10^−14^). Taken together, these data coupled with the finding that P3F1-TurboV5 rescued the loss of endogenous P3F1 suggest successful proximity labeling. Surprisingly, this analysis identified NCOA1 and NCOA2, a close homolog of MAML3 (MAML1), and members of the IND80 complex (INO80 and INO80E) in the P3F1 interactome at somewhat lower stringency (fold-change > 2, *p* < 0.05; Fig. 1E), suggesting that the full-length version of the C-terminal fusion partners of the other oncofusions, their paralogs, or related complex members reside within the P3F1 interactome.

### FP-RMS oncofusions are functionally interchangeable

**Fig. 2:**
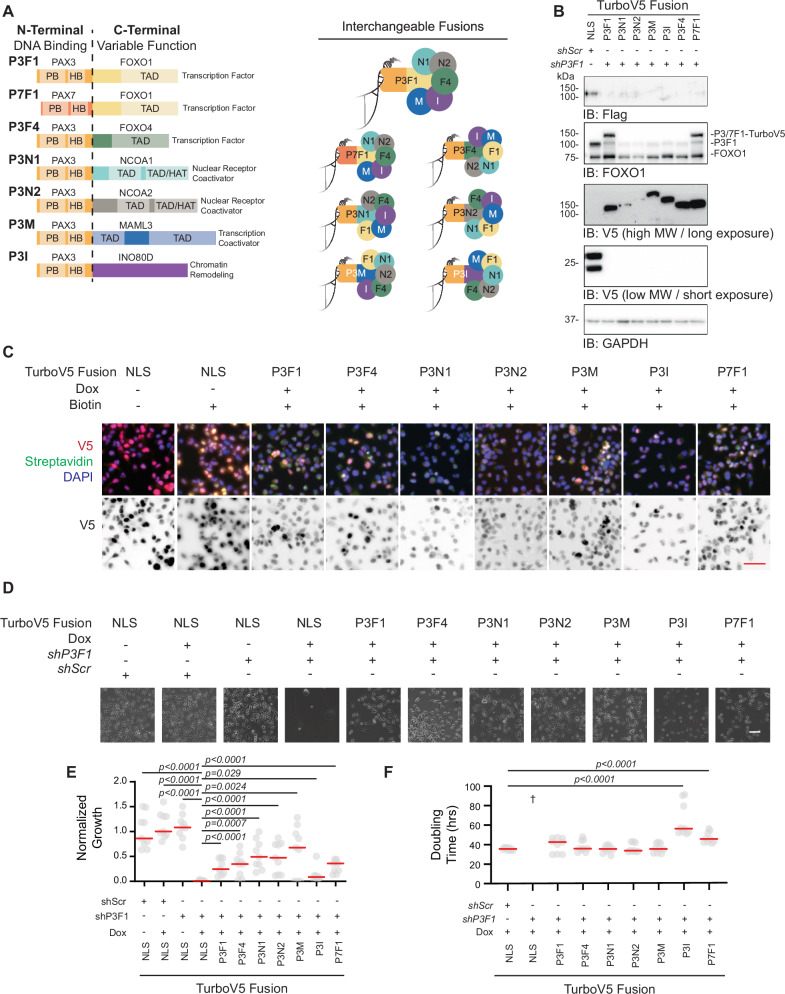
FP-RMS oncofusions rescue the loss of endogenous P3F1. **A** Diagram of PAX fusion proteins found in FP-RMS and a model of their possible interchangeable interactions. **B** Immunoblot analysis (IB) with the indicated antibodies from RH4-flag cells transduced with the indicated TurboV5 fusion and treated with doxycycline (Dox) to induce expression of the indicated shRNAs. Representative images of 2 replicate experiments. **C** Representative immunofluorescent micrographs of cells expressing TurboV5-tagged fusion genes. Due to drastic differences in expression, the V5 channel for each fusion was computationally brightened (histogram set 10-100) in comparison to TurboV5-NLS (histogram set 10-255). Scale Bar = 50 µm. (*n* = 3 images for each condition). **D** Representative phase-contrast micrographs of RH4-flag cells transduced with the indicated doxycycline-inducible shRNAs and TurboV5 fusion proteins after 13 days of growth. Scale Bar = 50 µm. (*n* = 3 images for each condition). **E** Bar plot of the quantified mean (red line) and individual normalized growth rate of RH4-flag cells from (**D**). Cells were induced with doxycycline on day 0, counted at day 13, and growth was normalized to RH4-flag cells transduced with *shScr*, TurboV5-NLS and induced with doxycycline. (*n* = 9 replicates from 3 independent experiments. Unpaired two-sided t-test). **F** Bar plot of the quantified mean (red line) and individual doubling times (circles) of each fusion rescue cell line determined after doxycycline induction of the indicated shRNA for 2 weeks before the start of the assay. Doubling times were determined by fitting exponential growth curves to the data presented in Suppl. Fig. 2B. (*n* = 9 replicates from 3 independent experiments. Unpaired two-sided *t*-test. † - no cells after 2 weeks of doxycycline induction). Source data are provided as a Source Data file.

Although the various FP-RMS oncofusions share a common PAX3 or PAX7 N-terminal DNA-binding domain^22–24^, their C-terminal fusion partners differ (Fig. 2A, Suppl. Dataset 2). While many of these oncofusions have been identified in histologically similar RMS, only P3F1 and P7F1 have been validated as the oncogenic driver of the disease. This raises the questions, does each oncofusion function as a driver mutation, do they generate phenotypically similar diseases, and if so, how? Our finding that the P3F1 interactome includes C-terminal fusion partners or their paralogs from other RMS oncofusions suggests that the different C-termini recruit the same proteins to drive oncogenic transcription (Fig. 2A). Such a model predicts that the different oncofusions should be functionally interchangeable. To test this, we generated lentiviral vectors encoding the six additional oncofusions fused to TurboV5. RH4-flag cells transduced with the doxycycline-inducible *shP3F1* or a scrambled shRNA sequence (*shScr*) were stably transduced with these six other TurboV5-oncofusions, in addition to P3F1-TurboV5 as a positive control and TurboV5-NLS as a negative control. The resultant cells were treated with doxycycline and analyzed for protein expression. Immunoblotting with anti-V5, -FLAG, and -FOXO1 antibodies confirmed robust knockdown of endogenous P3F1 and variable expression of the oncofusions (Fig. 2B), perhaps reflecting a selection for specific levels unique to the individual oncofusions^39^. Immunofluorescence microscopy of duplicate cultures with an anti-V5 antibody, streptavidin, and DAPI staining verified that each oncofusion localized to the nucleus and biotinylated proteins in a biotin-dependent manner (Fig. 2C).

We next monitored culture growth in triplicate over time. In control cells expressing *shP3F1* and TurboV5-NLS, P3F1 suppression rapidly impaired growth, whereas *shScr* RH4-flag cells expressing TurboV5-NLS proliferated normally in the presence of doxycycline. Initial growth assays showed variable rescue by the oncofusions (Fig. 2D, E), ostensibly due to transient imbalances in protein expression post-induction, as previously observed^39^. However, after two weeks of doxycycline-induced selection, cell doubling times were fully restored by five (~40 h) or partially restored by two (P3I-TurboV5, ~65 h and P7F1-TurboV5, ~47 h) oncofusions compared to control cells (Fig. 2F; Suppl. Fig. 2A, B). Similarly, while P3F1 suppression significantly reduced spheroid growth and colony formation, the TurboV5 oncofusions all rescued these phenotypes to control levels, with the exception of P7F1-TurboV5, which was somewhat less effective (Suppl. Fig. 2C–F). On the other hand, the loss of P3F1 could not be rescued by ectopic expression of Turbo-V5-tagged FOXO1 (Suppl. Fig. 2G–I). Finally, to ensure that the oncogenic growth imparted by each oncofusion was specific to both RMS and the C-terminal fusions, the above cell-growth rescue assays were repeated using a TurboV5 version of the transcription oncofusion *EWSR1*::*FLI1* (EF-TurboV5) found in Ewing’s sarcoma^41^ or its transactivation domain fused to *PAX3* DNA-binding domains (EP3-TurboV5). While immunoblot with an anti-V5 antibody confirmed that these proteins were expressed in the absence of P3F1 (Suppl. Fig. 2A), neither EF-TurboV5 nor EP3-TurboV5 restored growth after P3F1 knockdown (Suppl. Fig. 2J). Thus, despite their divergent C-termini, RMS oncofusions interchangeably drive oncogenic growth (Fig. 2A).

### Different oncofusions share a common transcriptional program and chromatin interactions

**Fig. 3:**
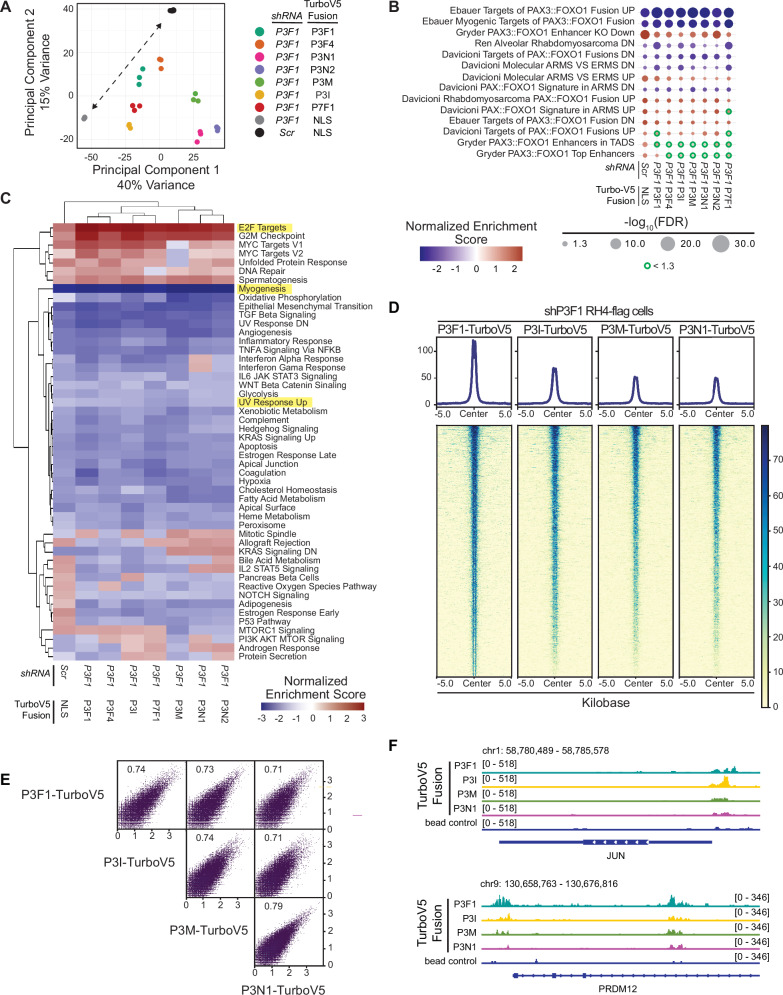
A common core transcriptional response of oncofusions aligns with common chromatin interactions. **A** A plot of the first two principal components from transcriptional profiling of RH4-flag cells expressing the indicated shRNAs and TurboV5 oncofusions. Arrow indicates the axis between the positive and negative control samples. (*n* = 3 technical replicates per condition). **B** A bubble heatmap of RMS-related gene signatures from MSigDB C2: Curated gene sets. GSE analysis was performed using the entire C2 gene set library and gene sets were reported based on the relationship to FP-RMS. Each column is in comparison to RH4-flag cells expressing shP3F1 and TurboV5-NLS. (*n* = 3 technical replicates per condition. Empirical permutation test. *p*-values adjusted using Benjamini-Hochberg correction. Green circles: FDR > 0.05). **C** A heatmap of normalized enrichment scores for MSigDB hallmark gene sets, clustered by hierarchy. Each column is in comparison to RH4-flag cells expressing shP3F1 and TurboV5-NLS. (*n* = 3 technical replicates per condition. Yellow highlights: gene sets with normalized enrichment scores in the same direction and an FDR < 0.05 across all samples). **D** Heatmap of CUT&Tag signals for TurboV5-tagged P3F1, P3I, P3M or P3N1 ectopically expressed in shP3F1 RH4-flag cells, from ± 5 kb of the called P3F1 peaks. **E** Scatter plots showing the correlation between the CUT&Tag signals of P3F1, P3I, P3M, and P3N1. Pearson correlation coefficient values are indicated at the upper left of each plot. **F** Integrative Genome Viewer (IGV) tracks of indicated CUT&Tag signal of TurboV5 tagged P3F1, P3I, P3M, and P3N1 at known P3F1 targets in *shP3F1* RF4-flag cells.

As the different oncofusions were largely interchangeable, it stands to reason that they drive a common transcription program to promote oncogenesis (Fig. 2A). To test this, mRNA was isolated from triplicate cultures of *shP3F1* RH4-flag cells in which endogenous P3F1 loss was rescued by each of the seven oncofusion-TurboV5 proteins, as well as from the controls of *shScr* RH4-flag cells expressing TurboV5-NLS induced with doxycycline and *shP3F1* RH4-flag cells expressing TurboV5-NLS (induced for five days with doxycycline to reduce endogenous P3F1 expression but maintain the cell population). High-throughput RNA sequencing determined transcript abundance (Suppl. Dataset 3). Normalized transcript data was subjected to Principal Component Analysis (PCA), which revealed that replicates clustered tightly, indicating high data quality. Plotting along an axis between positive and negative controls showed that the group of P3F1, P3F4, and P7F1 oncofusions and, to some extent, P3I closely resembled the endogenous P3F1 transcriptome, while P3M, P3N1, and P3N2 displayed distinct transcriptional signatures, consistent with the C-termini derived from very different proteins (Fig. 3A).

Given that the different oncofusions rescued the loss of P3F1, we tested whether they share any similarity with known transcriptional signatures of P3F1 or FP-RMS. Differential expression analysis compared each sample to control *shScr* RH4-flag cells expressing TurboV5-NLS, followed by Gene Set Enrichment Analysis(GSEA) using MSigDB C2 curated gene sets. Censoring for P3F1 and aRMS gene sets^8,15,16,42,43^ revealed Normalized Enrichment Scores (NES) trending similarly across all samples (Fig. 3B; Suppl. Dataset 4), including the *shScr* RH4-flag cells expressing TurboV5-NLS. Thus, all oncofusions produce transcriptional changes consistent with P3F1 and aRMS. Furthermore, GSE analysis using MSigDB hallmark gene set^44^ identified three gene sets with consistent NES trends and an FDR < 0.05. Namely, upregulation of “E2F Targets” and downregulation of “Myogenesis” and “UV Response UP” representing known biological states driving FP-RMS (Fig. 3C; Suppl. Dataset 5)- promoting proliferation and inhibiting myogenic differentiation^8^. Taken together, despite differences in their overall transcriptomes, the different oncofusions converge upon a common core transcriptional program associated with oncogenic features of FP-RMS.

Since the different oncofusions share a common core transcriptome, we tested whether these oncofusions also share chromatin-binding functions. CUT&Tag^45^ was performed with a V5 antibody and *shP3F1* RH4-flag cells rescued with a TurboV5-tagged oncofusion from each of the four aforementioned groups (P3F1, P3I, P3M, or P3N1). Sequencing analysis showed significant overlap and correlation in genomic localization (Fig. 3D, E), with over 900 shared peaks (Suppl. Fig. 3A). Among these shared peaks, ~45% localized to promoters, including known P3F1 target genes^8,15,16,43^ such as *JUN*, *PIPOX*, and *MYOD1*. Similarly, acute degradation of P3F1 has been linked to downregulation of *KLF4*, *FGGY*, and *PRDM12*^34^, and these genes were bound by all four oncofusions (Fig. 3F, Suppl. Fig. 3B). Motif-enrichment analysis of the 900 shared peaks also revealed significant enrichment of P3F1 and myogenic transcription factor motifs (*MYF5*, *MYOG*, and *MYOD*; Suppl. Fig. 3C), genes that are crucial for maintaining RMS transcriptional programs^46^. In summary, each oncofusion generates a core transcriptional output related to P3F1-driven oncogenesis, share a substantial number of chromatin interactions previously identified as P3F1 targets, and are interchangeable in their proliferative ability, suggesting that each drive an analogous disease state through a shared aberrant epigenetic reprograming of the cell.

### The different oncofusions share a common interactome

**Fig. 4:**
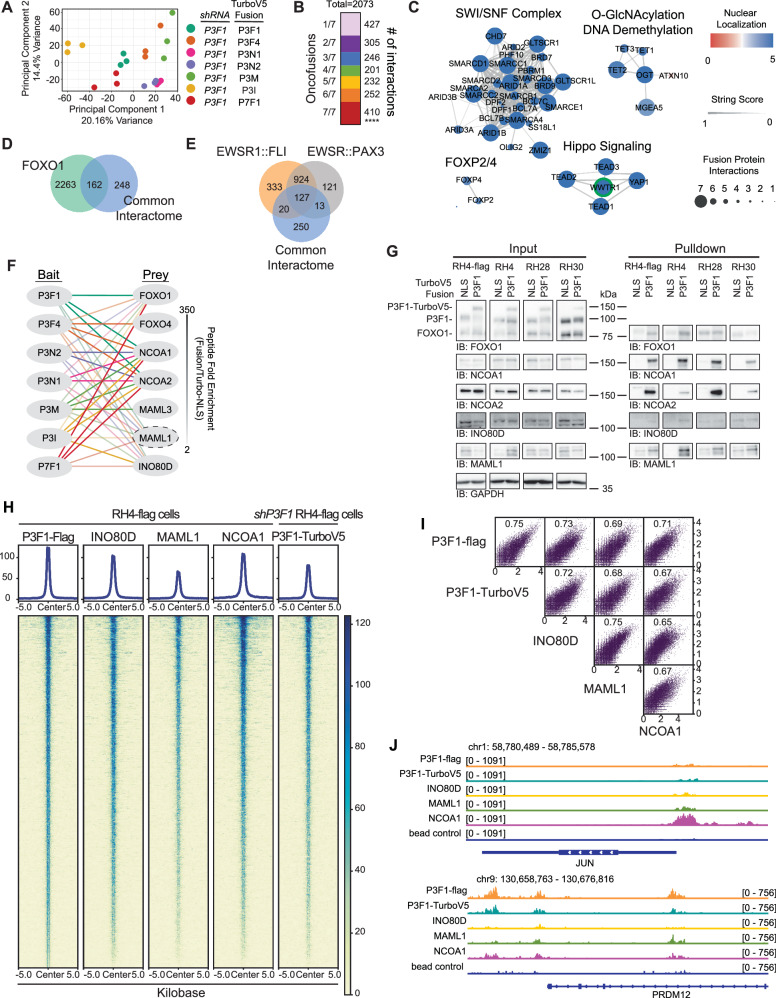
The interactome of each FP-RMS oncofusion shares a common set of proteins including the C-terminal fusion partners of the other oncofusions. **A** A scatter plot of the first two principal components from proximity labeling of RH4-flag cells with the indicated shRNA and TurboV5 fusion proteins. (*n* = 3 technical replicates per condition). **B** Diagram of overlapping fusion protein interactions. Each layer represents increasing number of oncofusion proteins which identified the same proteins by proximity labeling (fold change > 1.5, *p* < 0.05, two-tailed heteroscedastic t-test. Binomial test ****p < 10^−15^). **C** Markov clustered STRING interaction subnetworks of all proteins identified in (**B**). Clusters of interest were manually selected. Full network is provided in Suppl. Fig. 4. WWTR1 is highlighted in green. Clusters annotated using STRING enrichment analysis. **D** Venn diagram of interactions identified in the common interactome compared to FOXO1-TurboV5. **E** Venn diagram of interactions identified in the common interactome compared to EF-TurboV5 and EP3-TurboV5. **F** Network of TurboV5 oncofusion baits and the full-length proteins of C-terminal fusion partners. MAML1 is demarcated since it is not found in a fusion but a close homologue of MAML3 (*see* Discussion). **G** Representative immunoblot with the indicated antibodies from the indicated human RMS cells transduced with P3F1-TurboV5 or TurboV5-NLS and treated with biotin for 16 h. Pulldown of the indicated biotinylated proteins was performed by streptavidin affinity-capture. (representative of *n* = 2). **H** Heatmap of CUT&Tag signal for endogenous P3F1 (Flag-tagged) and its co-regulators (INO80D, MAML1 and NCOA1) in RH4-flag cells, as well as those of V5-tagged ectopically-expressed P3F1 in *shP3F1* RH4-flag cells from ± 5 kb of the called endogenous P3F1 peaks. **I** Scatter plots of the correlation between the CUT&Tag signals of endogenous P3F1, ectopic P3F1, and the co-regulators INO80D, MAML1, and NCOA1. Pearson correlation coefficient values are indicated at the upper left of each plot. **J** Integrative Genome Viewer (IGV) tracks showing the indicated CUT&Tag signals of endogenous P3F1, ectopic P3F1, and the co-regulators INO80D, MAML1, and NCOA1 at the known P3F1 targets in RH4-flag cells. Source data are provided as a Source Data file.

To explore whether the different oncofusions are functionally interchangeable via a core set of shared proteins, *shP3F1* RH4-flag cells rescued with each of the seven oncofusion-TurboV5 proteins and *shScr* RH4-flag cells expressing TurboV5-NLS as a control were cultured in triplicate with biotin and labeled proteins were affinity captured and subjected to mass spectrometry. This identified 4,362 proteins across all samples. Enrichment values calculated in comparison to TurboV5-NLS samples (Suppl. Dataset 6) were subjected to PCA. This revealed that replicates clustered tightly, with P3F1, P3F4, and P7F1 forming one cluster, P3N1 and P3N2 forming another, and P3M and P3I being the most divergent (Fig. 4A), consistent with differences in their C-termini. The interactomes were then subjected to a low stringency enrichment threshold (fold-change > 1.5, *p* < 0.05) to favor commonality between oncofusions over strict statistical enrichment, predictably at the expense of more false-positives. This identified between 1072 and 1229 proteins enriched in each oncofusion sample, or 2073 unique proteins across all samples. However, by then applying the stringent criteria of proteins enriched by all seven oncofusions, this was reduced to 410 proteins (Fig. 4B). For context, less than one protein from the total of 4362 proteins identified by mass spectrometry is expected to be enriched in all seven oncofusions using the above threshold. Hence, the identification of 410 proteins is highly significant (*p* = 2.15 × 10^−^^13^), and as such, is defined as a common interactome.

We next generated a protein-interaction network using the STRING database, censored for experimentally validated interactions^47^. Clustering the network into 162 functional groups revealed enrichment in nuclear localization and clusters previously linked to P3F1 such as SWI/SNF (BAF) and Hippo signaling pathways^32^. This lends credence to the idea that other shared clusters may mediate a common oncogenic activity. To this end, we censored these 162 clusters for those having at least one protein found in all seven interactomes, which revealed 27 clusters with a range of functions not previously linked to these oncofusions (Fig. 4C; Suppl. Fig. 4). Case in point, we identified a cluster containing MGEA5, OGT, and TET2, perhaps suggesting transcriptional regulation through coincident DNA methylation and O-GlycNAcylation of histones^48^. Thus, the common interactome potentially contains multiple functional clusters.

We similarly determined the interactomes of FOXO1-TurboV5, EF-TurboV5 and EP3-TurboV5 expressed in RH4-flag cells (Suppl. Dataset 7 and 8). Using the same low-stringency threshold as above (fold-change > 1.5, *p* < 0.05), the FOXO1 interactome shared 162 proteins with the common interactome (Fig. 4D, Suppl. Fig. 5A), with the remaining 239 proteins in the common interactome presumably reflecting oncofusion-specific interactions. EF-TurboV5 and EP3-TurboV5, which again do not rescue the loss of P3F1 (Suppl. Fig. 2J), share 160 proteins with the common interactome (Fig. 4E; Suppl. Fig. 5B), with the remaining 210 common interactome proteins unique to P3F1, consistent with the common interactome not simply being a generic transcriptional complex, but having some specificity for FP-RMS oncofusions.

In summary, although the interactomes grossly differ between oncofusions, all seven nevertheless contain a common set of core proteins, suggesting that this common interactome is enriched with proteins that imbue each oncofusion with a common oncogenic activity unique to FP-RMS.

### C-terminal fusion partners reside within the common interactome

As observed with P3F1-TurboV5, the interactomes of each of the other FP-RMS oncofusions contained the proteins corresponding to the full-length versions of the C-terminal fusion partners from the other oncofusions, with two exceptions, FOXO4 and MAML3. However, even in these cases, a paralog was identified, namely FOXO1 in place of FOXO4, and MAML1 in place of MAML3 (Fig. 4F). Further, all these fusion partners were unique to FP-RMS, as they were not enriched in the EP3- or EF-TurboV5 interactomes (Suppl. Fig. 5B; Suppl. Dataset 8). To independently validate these interactions, we tested whether the full-length version of C-terminal fusion partners from the other oncofusions was captured by P3F1-TurboV5 in an expanded set of human FP-RMS cell lines. RH4, RH4-flag, RH28, and RH30 cell lines were stably infected with lentiviruses encoding either P3F1-TurboV5 or TurboV5-NLS as a control. The eight cell lines were then cultured with biotin, and labeled proteins were affinity-captured as above, followed by immunoblotting with anti-FOXO1, -NCOA1, -NCOA2, -MAML1, and -INO80D antibodies. This confirmed appropriate expression of P3F1-TurboV5, and moreover, that each of the six different full-length C-terminal fusion partners or their paralogs were affinity captured from all four P3F1-Turbo cell lysates at a greater extent compared to control TurboV5-NLS lysates. The only exceptions were that FOXO1 and INO80D were captured by P3F1-TurboV5 and TurboV5-NLS in RH30 cells (Fig. 4G). Thus, we validate that the P3F1 interactome contains the full-length C-terminal fusion partners or their paralogs in multiple human FP-RMS cancer cell lines.

To test whether these full-length fusion partners co-localized with the oncofusion at chromatin sites critical for FP-RMS oncogenesis, CUT&Tag was performed using Flag, INO80D, MAML1, and NCOA1 antibodies in parental RH4-flag cells. As expected, we observed similar chromatin localization between endogenous flag-tagged and previously tested ectopic TurboV5-tagged P3F1 (Fig. 4H, I). We also observed a close correlation of chromatin localization between endogenous P3F1 and INO80D, MAML1 and NCOA1 (Fig. 4H, I), with over 800 shared peaks (Suppl. Fig. 6A), including at the known targets of P3F1- *JUN*, *MYOD1*, *PIPOX*, and *PRDM12* (Fig. 4J; Suppl. Fig. 6B). Motif analysis further highlighted enrichment of *MYF5*-, *MYOG*-, *MYOD*-, and P3F1-binding sites at shared targets (Suppl. Fig. 6C). Thus, the full-length C-terminal fusion partners are part of the common interactome and co-localize with P3F1 on chromatin. These findings suggest a model whereby the DNA-binding domain of PAX3 or PAX7 fused to components within the common interactome recruit the same core set of proteins to chromatin and drive oncogenesis through a common transcriptional program (Fig. 2A).

### The common interactome predicts a new oncofusion in RMS

**Fig. 5:**
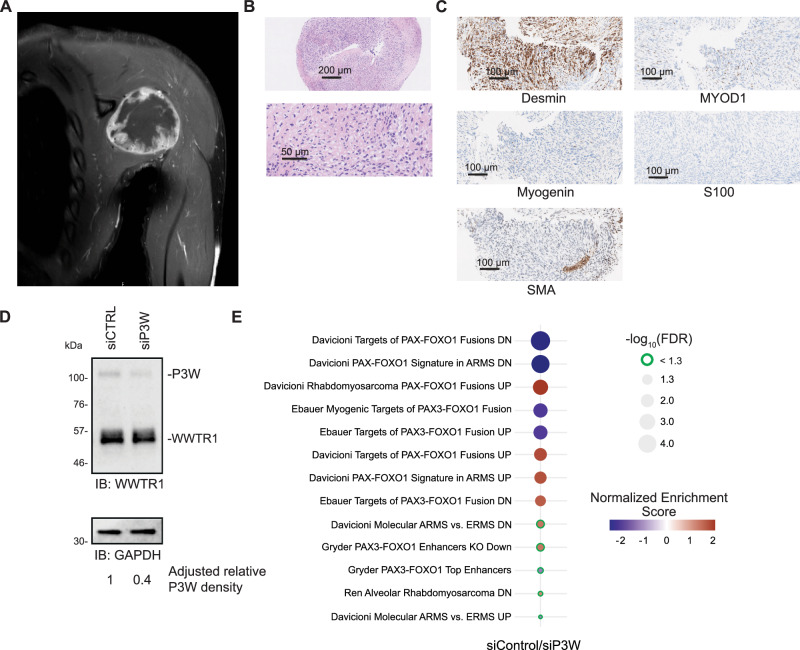
The predicted *PAX3::WWTR1* gene fusion is an oncogenic driver in a rhabdomyosarcoma patient. **A** T1-weighted image with fat suppression, post-contrast, showing a heterogeneous lesion with a maximum diameter of 7 cm. (*n* = 1). **B** Representative H&E staining of the tumor specimen (magnification 8x and 40x). Scales included. (*n* = 1). **C** Representative IHC staining of the tumor specimen for conventional RMS markers (desmin, MYOD1, myogenin; magnification 16x) and markers for biphenotypic sinonasal sarcoma. (S100, SMA; magnification 16x). Scales included. (*n* = 1). **D** Immunoblot (IB) analysis with the indicated antibodies from RMS000EEC tumoroids derived from the patient after transfection with control or *P3W* targeted siRNA. *Bottom*: quantification. GAPDH served as loading control. (*n* = 1). **E** A bubble heatmap of RMS-related gene signature enrichment scores comparing control and knockdown *P3W* (KD) siRNA RMS000EEC cells. (*n* = 2 technical replicates. Green circles: FDR < 0.05). Source data are provided as a Source Data file.

One prediction of the above model is that any component of the common interactome could, in principle, be a potential C-terminal fusion partner. To this end, we note a recent RMS case in which the patient presented with heterogeneous swelling in the axilla, which was fluorodeoxyglucose avid with signs of central necrosis on imaging (Fig. 5A) and histologically comprised of small round blue cells that morphologically resemble eRMS (Fig. 5B). Tumor cells stained positive for Desmin, MyoD1, and Myogenin, conventional RMS markers, and negative for Smooth Muscle Actin (SMA) and S100, markers of the related biphenotypic sinonasal sarcoma^42^, further supporting an RMS diagnosis (Fig. 5C). Previous STAR Fusion analysis of RNA sequencing failed to identify a *P3F1* transcript but instead found an undescribed fusion transcript consisting of *PAX3* fused to *WWTR1*^49^. We note that WWTR1 is a component of the common interactome within a Hippo pathway cluster (Fig. 4C; Suppl. Fig. 4), a pathway previously implicated in RMS, although not directly through the oncofusion^50,51^. The discovery of this new fusion transcript (termed P3W), detected in both the original tumor and a subsequent pulmonary relapse, is suggestive of a new oncofusion. The question raised for this patient was whether to consider histology as a favorable risk factor (i.e., eRMS) or if this is a new oncofusion and should be considered unfavorable (i.e., aRMS).

To begin addressing this question, PCA was performed on the patient RNA-seq data, but analysis failed to specifically cluster the tumor with either fusion-negative eRMS or fusion-positive aRMS samples (Suppl. Fig. 7A). Given this, we investigated whether P3W was indeed an oncofusion. We had previously generated a tumor organoid (tumoroid) culture line (RMS000EEC) from this patient^52^, which we subjected to *siRNA* targeting the unique *P3W* fusion breakpoint (*siP3W*). Immunoblotting with an anti-WWTR1 antibody detected the WWTR1 protein as well as a protein of approximately 110 kDa, the estimated size of the P3W, in the parental tumoroids, which was ablated upon transduction with *siP3W* (Fig. 5D). RT-qPCR also validated a reduction of *P3W* mRNA by *siP3W* (Suppl. Fig. 7B). mRNA from these tumoroids was further subjected to RNA sequencing (Supple. Dataset 9). STAR Fusion analysis of the sequenced transcripts showed a significant reduction of fusion transcripts in cells transfected with *siP3W* (Suppl. Fig. 7C), independently verifying the *siRNA* function and validating the STAR fusion detection of the fusion transcript. GSE analysis of the RNA-seq dataset further revealed aRMS- and P3F1-related gene sets were similarly altered by *siP3W* in the tumoroids (Fig. 5E). Finally, pathway analysis of differentially expressed genes showed that loss of P3W expression resulted in the activation of pathways involved in muscle differentiation (Suppl. Fig. 7D). Collectively, these data support P3W as an oncofusion driving RMS. As WWTR1 is one of the 410 proteins comprising the common interactome (Fig. 4C; Suppl. Fig. 4) that was not biotinylated by either EF- or EP3-TurboV5 (Suppl. Fig. 5B; Supple. Dataset 8), the common interactome predicted an oncofusion.

### The common interactome is enriched with FP-RMS gene dependencies

**Fig. 6:**
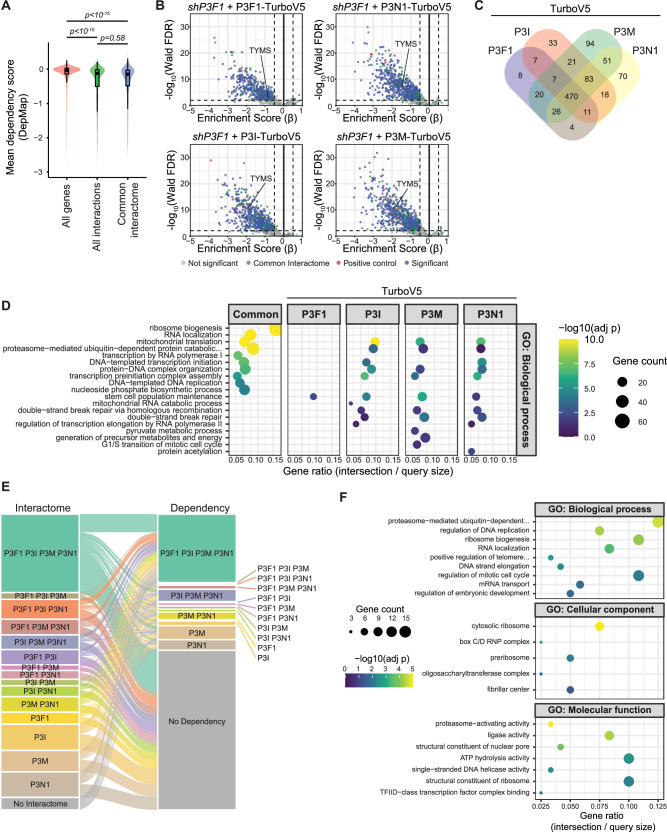
The FP-RMS oncofusion interactome is enriched with genetic dependencies including common and fusion-specific dependencies. **A** Violin-box plots of mean DepMap dependency scores for all genes in seven FP-RMS cell lines, the 2,073 proximity-labeled oncofusion interactome proteins (all interactions), or the 410 common interactome proteins. Center line: median; box: 25th–75th percentiles; whiskers: ±1.5× IQR. (Two-sided Kolmogorov-Smirnov test). **B** Volcano plots of sgRNA β enrichment scores (late versus early timepoints) in RH4-flag cells expressing shP3F1 and the indicated oncofusion-TurboV5. (*n* = 3 technical replicates. Dotted lines: FDR < 0.01 and *β* < −0.5). **C** Venn diagram of negatively enriched genes (*β* < −0.5, FDR < 0.01) from (**B**) across the four rescue conditions. **D** GO Biological Process enrichment bubble plot for sgRNA-target genes negatively enriched uniquely in each rescue condition or across all four (common). Gene ratio: intersection/query size. Circle size: gene count; color: −log10(adjusted p). (Hypergeometric test, g:SCS correction). **E** Sankey plot linking proximity-labeled proteins from the 2,073-protein interactome (Fig. 4B) to negatively enriched sgRNA targets from B for the indicated oncofusion combinations. No interactome: proteins uniquely labeled by the oncofusions that were not tested in the loss-of-function CRISPR/Cas9 screen. No dependency: genes not negatively enriched in any condition. **F** GO Biological Process, Cellular Component, and Molecular Function enrichment for the 120 proteins that are both common interactome members and dependencies (from **E**). Gene ratio, circle size, and color as in (**D**). (Hypergeometric test, g:SCS correction).

It stands to reason that genes encoding proteins that associate with the oncofusions are likely enriched for FP-RMS genetic dependencies. To test this in silico, we turned to the Cancer Dependency Map (DepMap)^53^ and assessed distribution of mean dependency scores of all FP-RMS cell lines in the database. Comparing the dependency scores of all identified interactions (2,073) and the common interactome (410) genes to all genes assayed in DepMap revealed a statistically significant negative shift in dependency scores signifying an enrichment for genes on which FP-RMS is dependent (Fig. 6A). Given this, we designed a focused CRISPR loss-of-function library targeting all 2,073 interactors, along with essential-gene and negative controls (Suppl. Dataset 10), and performed pooled screening in RH4-flag cells expressing inducible *shP3F1* rescued with P3F1-, P3N1-, P3I-, or P3M-TurboV5. MAGeCK-MLE analysis revealed a correlation in sgRNA enrichment between the four cell lines (Suppl. Fig. 8A), with most being negatively enriched (Fig. 6B, Suppl. Dataset 11), consistent with shared functional programs driven by the oncofusions.

Using thresholds defined by the essential-gene controls (FDR < 0.01, *β* < –0.5), we identified 923 dependencies (Fig. 6C). We note that each oncofusion had 8 to 94 unique dependencies, suggestive of specific activities related to the function of the C-terminal fusion partner. For example, the C-terminus of P3N1 encoded the acetyltransferase domain of NCOA1, and GO analysis of the unique dependencies in P3N1-driven cells identified selective enrichment for genes involved in protein acetylation (Fig. 6D). Nevertheless, the majority (71%) of the dependencies were shared by three or more oncofusions. GO enrichment analysis of dependencies shared by all four oncofusions revealed pathways associated with ribosome biogenesis, translation, transcription, and DNA replication (Fig. 6D, Suppl. Fig 8B), processes associated with general oncogenic proliferation^54^ and FP-RMS biology^8,55,56^.

We next explored the relationship between protein interactions and genetic dependencies by Sankey analysis. Namely, we mapped all the proteins specifically biotin labeled by any of the tested TurboV5-oncofusions (from 2,073 in total, Fig. 4B) that were found in one, two, three or all four of the P3F1, P3I, P3M, and P3N1 interactomes against their dependency (Fig. 6C) in *shP3F1* RH4-flag cells rescued by one, two, three or all four of these oncofusions (Fig. 6E). More than half of the genes tested exhibited no dependency, and generally there was not clear relationship between the degree a protein was associated with the different oncofusions and dependency, except in one case. Namely, the common interactome contributed the single largest number of genes (120) to the common dependency class, suggesting that interactions shared across all four oncofusions are the most reliable predictor of shared vulnerabilities. Furthermore, GO analysis of these 120 genes revealed enrichment for the same core pathways identified for the broader common dependency set – ribosome and translation, transcription, and DNA replication (Fig. 6F). Beyond commonalities, we also identified oncofusion-specific interactions/dependencies. Namely, P3N1, P3I, and P3M uniquely associated with 10, 1, and 3 proteins in which the corresponding sgRNAs were also negatively enriched only in cells rescued with the same oncofusion. These included proteins with protein regulatory functions such as the kinase MAP2K7 and E3 ubiquitin ligase LTN1 for P3N1, perhaps reflecting oncofusion-specific activities. In summary, the interactomes contain proteins required for oncogenesis driven by all as well as specific oncofusions.

### The P3F1 transcriptional program is mediated by interactome proteins

**Fig. 7:**
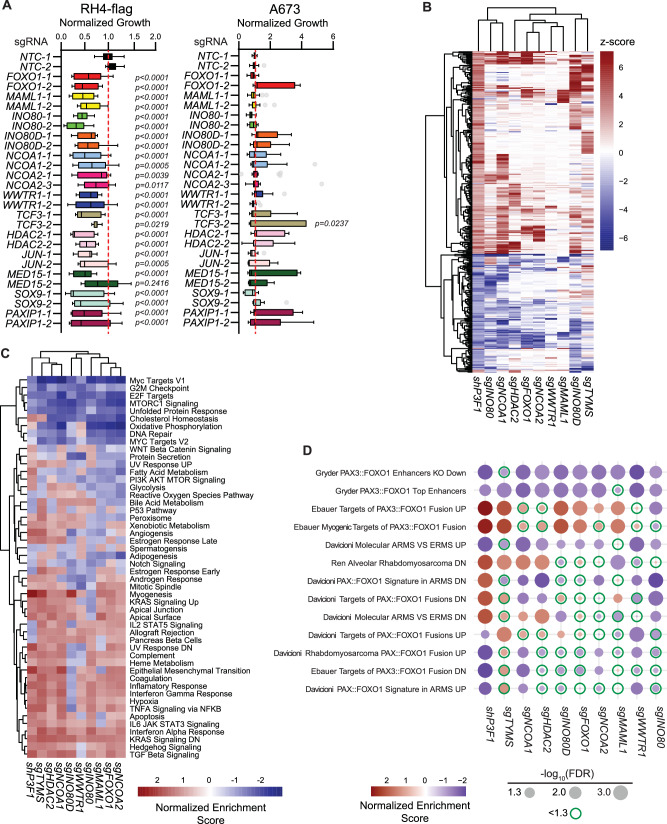
Candidate FP-RMS oncofusion interactions reinforce P3F1 transcriptional program. **A** Box and whisker plot of normalized growth of RH4-flag and A673 cells transduced with the indicated sgRNA. Values are normalized to the *sgNTC* (non-targeting control) transduced cells. Center line: median. Box: 25^th^ to 75^th^ percentiles. Whisker: 75^th^ or 25^th^ percentile plus or minus 1.5x IQR. (RH4-flag: *n* = 12 wells per condition from 3 individual experiments, A673: *n* = 16 wells per condition from 4 individual experiments, one way ANOVA). **B** A heatmap of z scores corresponding to mRNA expression changes comparing RH4-flag cells transduced with the indicated shRNA or sgRNA versus *shScr* or *sgNTC*, clustered by hierarchy. The transcripts depicted are differentially expressed (2 standard deviations from the mean, *p*-adj < 0.05. Wald test, p-values adjusted using Benjamini-Hochberg correction) in *shP3F1* compared to *shScr* and at least one of the other samples. (*n* = 3 technical replicates per condition). **C** A heatmap of normalized enrichment scores for MSigDB hallmark gene sets, clustered by hierarchy. Each column is in comparison of RH4-flag cells transduced with the indicated shRNA or sgRNA versus *shScr* or *sgNTC*. (*n* = 3 technical replicates per condition). **D** A bubble heatmap of RMS-related gene signatures from MSigDB C2: Curated gene sets. GSE analysis was performed using the entire C2 gene set library and gene sets were reported based on the relationship to FP-RMS. Each column is in comparison of RH4- RH4-flag cells transduced with the indicated shRNA or sgRNA versus *shScr* or *sgNTC*. (*n* = 3 technical replicates per condition. Green circles represent FDR > 0.05. Empirical permutation test, *p*-values adjusted using Benjamini-Hochberg correction). Source data are provided as a Source Data file.

To assess how the common interactome influences oncofusion-driven transcription, we first assembled a panel of lentiviral vectors expressing two independent sgRNAs against genes encoding proteins of the common interactome. Namely the full-length version of C-terminal fusion partners (FOXO1, MAML1, INO80D, NCOA1, NCOA2, and WWTR1) or common interactome proteins prioritized by proximity labeling enrichment and DepMap dependency (INO80, TCF3, HDAC2, JUN, MED15, SOX9, and PAXIP1). RH4-flag cells alongside two non-targeting controls (*sgNTC*) were stably infected with each of these 26 sgRNA vectors in triplicate, three to four independent times and evaluated for proliferation by Crystal Violet staining after two weeks. In parallel, A673 cells, derived from a human pediatric (Ewing’s) sarcoma driven by an oncofusion (EWSR1::FLI1), were similarly tested. With the exception of *sgMED15-*2, all these sgRNAs significantly reduced proliferation relative to *sgNTC*-1 of RH4-flag cells, but none in A673 cells (Fig. 7A).

Given that inactivating these common interactome genes in an FP-RMS cells line, but not another closely related fusion-positive cancer cell line, we selected a subset for RNA-seq analysis (INO80, HDAC2, FOXO1, MAML1, INO80D, NCOA1, NCOA2, and WWTR1). Triplicate independent cultures of RH4-flag cells stably infected with one of these sgRNAs targeting each of these eight genes or the non-targeting *sgNTC* were subjected to RNAseq. Fold-differences were determined (Suppl. Dataset 12), z scores calculated and compared to those derived from above RNA-seq analysis of RH4-flag cells in which P3F1 was inactivated and compared to RH4-flag *shScr* control cells (Fig. 3B). This revealed a positive correlation between inactivating interactome proteins versus inactivating P3F1 (Spearman coefficient = 0.042–0.245; Suppl. Fig. 9A). Transcripts differentially expressed along the positive correlation axis (> or < 2 SD from the mean; adjusted *P* < 0.05) were identified (Suppl. Fig. 9B), and plotted as a heatmap. This revealed similar transcriptional profiles induced by the loss of common interactome proteins and the loss of P3F1, with individual components of the common interactome appearing to contribute to different segments of the P3F1 transcriptome (Fig. 7B).

We next performed GSE analysis using MSigDB Hallmark gene sets and a post-hoc–censored MSigDB C2 collection focused on P3F1 and aRMS related sets. Hallmark NES values were strongly correlated across sgRNA samples (Spearman coefficient = 0.27–0.91; mean 0.592), with sgFOXO1 most closely resembling shP3F1 (Spearman coefficient = 0.65; Suppl. Fig. 9C). Permutation-based null models confirmed that the observed correlations far exceeded those expected by chance (Suppl. Fig. 9D). The commonly enriched pathways reflect repression of myogenic programs and increased activity of MYC targets, E2F targets, and G2M checkpoint genes, consistent with enhanced proliferation and impaired differentiation (Fig. 7C). Analysis using censored C2 gene sets further demonstrated that many perturbations alter curated P3F1 and aRMS transcriptional programs (Fig. 7D). Correlations across samples ranged from –0.12 to 0.90 (mean 0.527), with *sgINO80* showing an inverse signature and *sgFOXO1* again showing the strongest concordance with *shP3F1* (Suppl. Fig. 9E). Permutation testing again demonstrated that these correlations are highly unlikely to occur at random (Suppl. Fig. 9F). Collectively these data support the common interactome mediating the oncogenic transcriptional programs of the oncofusions.

### TYMS is a vulnerability of the common interactome

**Fig. 8:**
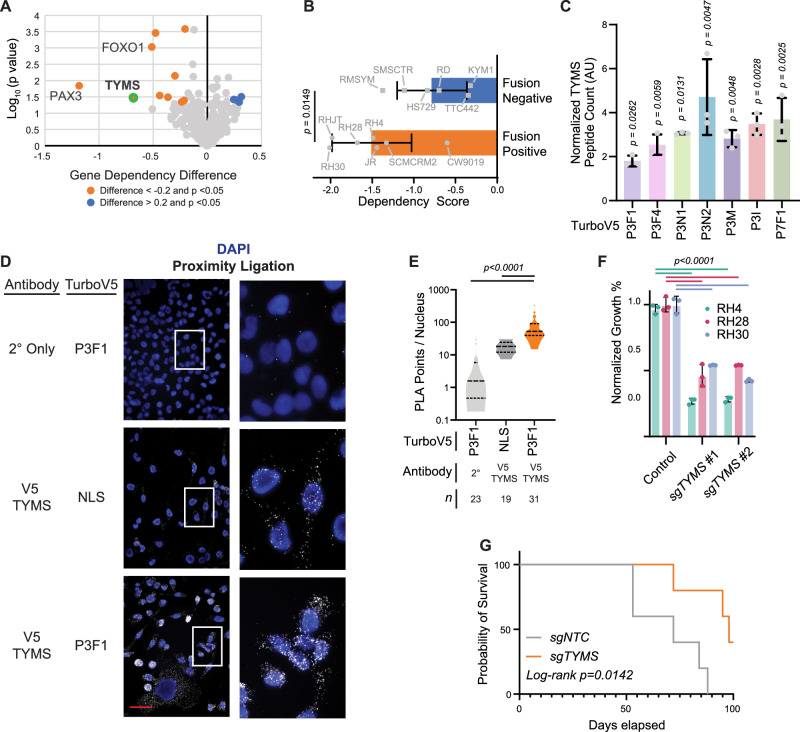
The FP-RMS common interactome protein thymidylate synthase is a common vulnerability. **A** Scatter plot of the difference in average dependency score from DepMap of the 410 interactome genes versus -Log_10_(p-value). Data points represent the difference between seven aRMS cell lines and 1,079 other cell lines. (two-sided Welch’s t-test). **B** Bar plot of the mean ± SD *TYMS* gene dependency scores from fusion-positive versus fusion-negative RMS cell lines. (*n* = 6 fusion negative cell lines and 7 fusion-positive cell lines. Two-sided *t*-test, *p < 0.05). **C** Bar plot of normalized mean ± SD TYMS peptide count (AU; Arbitrary Unit) identified by mass spectrometry of proteins streptavidin affinity-purified from RH4-flag cells in which the endogenous *PAX3::FOXO1* gene was inactivated and rescued with the indicated fusion-TurboV5 transgenes. Peptide counts were normalized to samples from TurboV5-NLS transduced cells. (two-tailed heteroscedastic t-test; *n* = 3 technical replicates per condition). **D** Representative micrographs of proximity-ligation assays (PLA) with the indicated primary antibodies and TurboV5 construct expressed in RH4 cells. Scale Bar = 50 µm. **E** Violin plot corresponding to D of quantified PLA points per nucleus (*n*: number of images analyzed from 2 biological replicates; one-way ANOVA). **F** Bar plot of mean ± SD normalized growth of crystal violet-stained cells in the indicated cell lines transduced with Cas9 and sgRNAs targeting *TYMS* in RH4, RH28, and RH30 cells. (*n* = 3 technical replicates per condition; one-way ANOVA). **G** Kaplan-Meier survival plot of mice bearing subcutaneous xenograft tumors derived from RH28 cells transduced with a vector encoding *sgNTC* (Non-Targeting Control) or *sgTYMS*. (*n* = 5 mice per condition, log-rank Mantel-Cox test). Source data are provided as a Source Data file.

As the above CRISPR screen of the common interactome yielded many dependencies (Fig. 6B), we posited that some common interactome proteins may be therapeutically actionable vulnerabilities. We thus turned to DepMap as a way to identify fusion-specific vulnerabilities. Namely, we compared the mean dependency scores of the 410 interactome genes across the seven FP-RMS cell lines in DepMap relative to 1,079 non–FP-RMS lines. As expected, sgRNAs targeting *PAX3* and *FOXO1* were amongst the largest negative dependency differences, likely reflecting direct targeting of the fusion itself (Fig. 8A). After these two, TYMS (Thymidylate Synthase) emerged as the top FP-RMS–selective vulnerability by dependency difference (Fig. 8A; Suppl. Fig. 5). Additionally, we note that *TYMS* sgRNAs were significantly depleted in the loss-of-function CRISPR screens in all four RH4-flag cell lines driven by P3F1-, P3I-, P3N1-, or P3M-TurboV5 (Fig. 6B), further credentialing TYMS as a vulnerability independent of the oncofusion. Further analysis of DepMap revealed that TYMS dependency scores were significantly lower in fusion-positive compared to fusion-negative RMS lines (Fig. 8B), indicating possible subtype selectivity.

Given the above, we validated that TYMS is a component of the common interactome. First, TYMS is in close proximity to each of the seven oncofusions. Namely, proximity labeling of TYMS by each of the oncofuion-TurboV5 was increased 1.8- to 4.7-fold relative to the TurboV5-NLS control (Fig. 8C). Second, TYMS is in close proximity to other common interactome proteins. Namely, comparing BioGRID-annotated TYMS interactors with the 410 common interactome proteins revealed eight overlapping proteins, a significant enrichment (Suppl. Fig. 10A, B; Fisher’s exact test *p* = 0.00135). Third, TYMS co-localizes with the oncofusion. Namely, proximity ligation assays (PLA) demonstrated a > 4-fold increase in TYMS–P3F1 puncta per nucleus in RH4-flag cells expressing P3F1-TurboV5 compared to control cells expressing *shCtrl* and TurboV5-NLS (Fig. 8D, E). Interestingly, co-staining the nucleus with DAPI revealed that interactions take place in and outside of the nucleus. Together, these results show that FP-RMS oncofusions and TYMS reside in close proximity to one another.

To explore the effect of TYMS on the FP-RMS transcriptome, we performed RNA-seq on RH4-flag cells expressing *sgTYMS* or *sgNTC*. While overall transcriptional changes showed minimal correlation with P3F1 depletion (Spearman coefficient = 0.022; Suppl. Fig. 9A, B), TYMS loss nevertheless affected a subset of P3F1-regulated transcripts (Fig. 7B). This contributed to a highly correlated pattern in GSEA using both hallmark and P3F1-associated gene sets (Fig. 7C, D; Suppl. Fig. 9C–F; Spearman coefficient = 0.56 and 0.51, respectively). Namely, TYMS loss enhanced myogenic differentiation programs, resulting in decreased MYC targets, E2F targets, and G2M checkpoint signature transcripts (Suppl. Fig. 10H), as observed for other common interactome proteins (Fig. 7C). These changes are consistent with increased differentiation and decreased proliferation programs, although interestingly, loss of TYMS does not appear the alter the occupancy of P3F1 on chromatin (Suppl. Fig. 10C).

We next tested the functional requirement for TYMS in FP-RMS. RH4, RH28, and RH30 cells were transduced with Cas9 and one of two independent *TYMS*-targeting sgRNAs or a non-targeting control. Immunoblot analysis verified TYMS loss in all three cell lines (Suppl. Fig. 10D). These nine cell lines were then assayed in triplicate of changes in growth as assessed by Crystal Violet staining one week later, which revealed that loss of TYMS by either sgRNA significantly reduced proliferation in all three FP-RMS lines compared to the control cells (Fig. 8F; Suppl. Fig. 10E). In combination with the previously described DepMap analysis and the targeted loss-of-function CRISPR screens we conclude that loss of TYMS inhibits the growth of FP-RMS cell lines.

To test whether this effect extended in vivo, RH28 cells were again stably infected with lentiviral vector expressing *sgNTC* or *sgTYMS-1*, *TYMS* knockout was confirmed by immunoblot (Suppl. Fig. 10F), and the cells transplanted into the flank of five SCID/*beige* mice each. This revealed a significant reduction in tumor growth upon loss of TYMS, namely no tumor formed in two mice, and in the other three grew significantly more slowly, resulting in a marked difference in overall survival compared to the *sgNTC* control tumors (Fig. 8G; Suppl. Fig. 10G), with one of these tumors exhibiting TYMS expression (Suppl. Fig. 10H). Collectively, these results support TYMS being a component of the common interactome required for tumorigenesis.

### FP-RMS cell lines are sensitive to pralatrexate

**Fig. 9:**
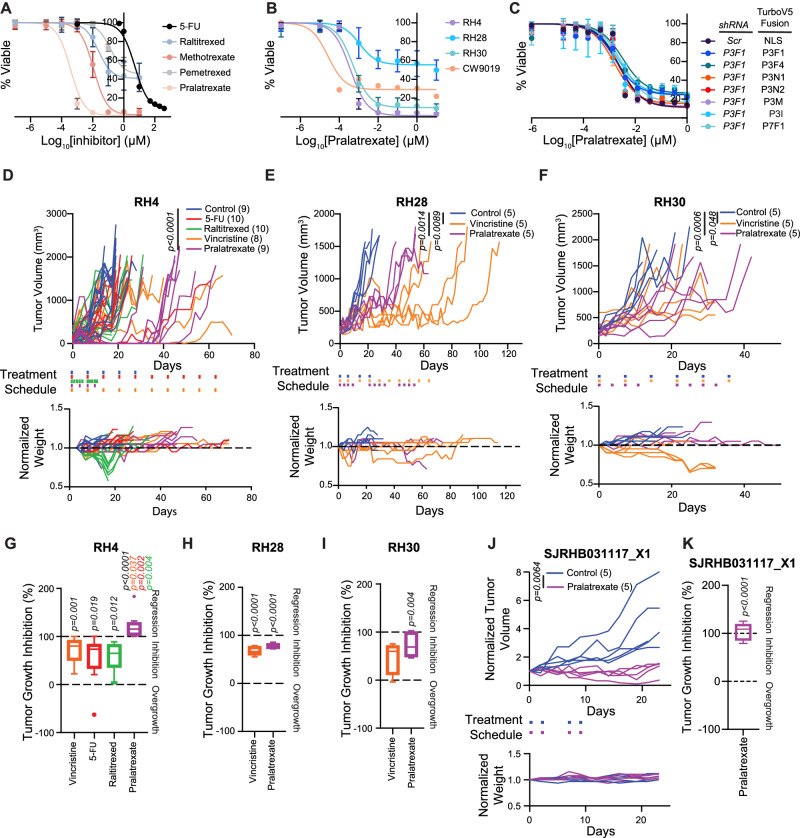
FP-RMS tumors are sensitive to inhibitors of folate metabolism. **A** Scatter plot of mean ± SD dose response and fit curves represented as percent (%) viability versus concentration of the indicated inhibitor for RH4 cells. **B** Scatter plot of mean ± SD dose response and fit curves represented as percent (%) viability versus concentration of pralatrexate for RH4, RH28, RH30, and CW9019 cells. **C** Scatter plot of mean ± SD dose-response and fit curves represented as percent (%) viability versus concentration of pralatrexate for RH4-flag cells in which the endogenous *PAX3::FOXO1* gene was inactivated and rescued with the indicated oncofusion-TurboV5 transgenes. Summaries of curve fitting, IC_50_ values, and *n* for each curve are reported in Supplementary Dataset 13. **D**–**F** Tumor volume (*top*), drug schedule (*middle*) and normalized animal weight (*bottom*) versus time of mice bearing subcutaneous xenograft tumors derived from (**D**) RH4, (**E**) RH28, or (**F**) RH30 cells treated with the indicated drugs or a vehicle control (*n* annotated in parathesis of key, mixed effect model, F-tests (Type III)). **G**–**I** Box and whisker plot of tumor growth inhibition (compared to vehicle control) of mice bearing subcutaneous xenograft tumors derived from (**G**) RH4, (**H**) RH28, or (**I**) RH30 cells treated with the indicated drugs. Center line: median; box: 25th–75th percentiles; whiskers: ±1.5× IQR. Blue asterisk: comparison to the vehicle control treated tumors. (one-way ANOVA; *n* is annotated in the related panels (**D**–**F**). **J** Tumor volume (*top*), drug schedule (*middle*), and normalized animal weight (*bottom*) versus time of mice (*n* = 5) bearing subcutaneous xenograft tumors derived from SJRHB031117_X1 patient derived xenografts treated with the indicated drugs or a vehicle control. (mixed effect model, F-tests (Type III)). **K** Box and whisker plot of tumor growth inhibition of mice bearing subcutaneous xenograft tumors derived from SJRHB031117_X1 patient derived xenografts treated with the indicated drugs. Center line: median; box: 25th–75th percentiles; whiskers: ±1.5× IQR. (two-sided t-test; *n* is annotated in the related panel **J**). Source data are provided as a Source Data file.

TYMS is a key enzyme in folate metabolism^57,58^, and this form of metabolism is targeted for the treatment of at least ten cancer types with the antifolates pemetrexed, raltitrexed, methotrexate, and pralatrexate or 5-fluorouracil (5-FU)^59–63^. Given this, we generated dose-response curves by measuring the number of metabolically active cells using the CellTiter-Glo reagent after 96 h of culturing RH4 cells in the presence of increasing concentrations of these four antifolates or 5-FU (Fig. 9A; Suppl. Dataset 13). Notably, pralatrexate exhibited the lowest mean IC_50_ (0.3712 nM) and maximal growth inhibition (nearly 100%) of all tested drugs. Furthermore, the mean IC_50_ values for 5-FU, raltitrexed, and pralatrexate were comparable to those reported for cell models^64–66^ corresponding to cancers in which these drugs are a standard component of treatment^60,62,63^. Give this, we expanded this analysis to RH28, RH30, and CW9019 human FP-RMS cancer cell lines. This revealed dose-response curves and IC_50_ values comparable to those observed in RH4 cells (Fig. 9B, Suppl. Fig. 11A, B; Suppl. Dataset 13). Importantly, pralatrexate yielded substantially lower IC_50_ values than the other two inhibitors in all cells. Notably, CW9019 cells are driven by P7F1, suggesting that FP-RMS cells may be broadly sensitive to antifolates and 5-FU, independent of the type of oncofusion. To explicitly test this, we repeated the analysis of pralatrexate in the aforementioned panel of RH4-flag cells whereby the endogenous *P3F1* gene was silenced and rescued with each of the seven oncofusion-TurboV5 proteins. Indeed, all exhibited sensitivity to pralatrexate at a level similar to control cells (*shScr* + TurboV5-NLS, Fig. 9C; Suppl. Dataset 13).

### FP-RMS tumors are sensitive to pralatrexate

Since 5-FU, raltitrexed, and pralatrexate showed in vitro efficacy in FP-RMS cells on par with the effect on cell lines derived from cancers treated with these drugs^64–66^, we next assessed the effect of these therapies on FP-RMS tumor growth in vivo. RH4 cells were implanted into the flanks of ten SCID/*beige* mice per treatment group, and once tumors reached 150 mm^3^, the mice were injected intraperitoneal at established doses and schedules^67–70^ with 5-FU (67 mg/kg, 1×/week for 10 weeks), raltitrexed (7.5 mg/kg, 5×/week for 2 weeks), pralatrexate (60 mg/kg, 2×/week for 2 weeks), vincristine (1 mg/kg, 1×/week for 10 weeks), or vehicle control (1x /week for 10 weeks). Some cohorts were reduced in size due to an absence of tumors or for other reasons. With the exception of raltitrexed, mice did not lose weight during the course of treatments, suggesting minimal toxicity (Fig. 9D). Tumors in vehicle-treated mice reached endpoint size within three weeks. Vincristine significantly inhibited tumor growth, with complete regression in two of eight tumors. Similarly, 5-FU suppressed tumor growth, with one complete regression, whereas raltitrexed produced minimal tumor growth inhibition. However, pralatrexate induced complete tumor regression in all mice in this cohort within two weeks despite the shorter treatment duration than vincristine, consistent with the sensitivity of these cells to these drugs in culture. Once pralatrexate treatment was terminated, however, regressed tumors eventually recurred (Fig. 9D). Given this, we extended this analysis to RH28 and RH30 xenografts (*n* = 5 per condition) with an extended pralatrexate dosing regimen (60 mg/kg, 3 week cycles of 2×/week for 2 weeks with a 1 week holiday for 9 weeks total) in comparison to treatment with vincristine as the positive control and vehicle as the negative control. Pralatrexate significantly inhibited tumor growth in both tumor models (Fig. 9E, F). To assess the degree of inhibition in each tumor model, the percentage of tumor growth inhibition was calculated for each drug at the time the first tumor in vehicle-control mice reached the tumor size end-point. This analysis confirmed that vincristine, 5-FU, and raltitrexed significantly inhibited the growth of tumors generated from RH4 cells, while pralatrexate significantly outperformed these drugs and caused regression in these tumors (Fig. 9G) as well as significantly inhibited growth of tumors derived from RH28 and RH30 cells (Fig. 9H, I). Immunoblot analysis of tumor lysates derived from these mice revealed a trend or significant increase in dihydrofolate reductase in RH4- and RH30 and -derived tumors from mice specifically treated with pralatrexate (Suppl. Fig. 11C, D), perhaps related to the observation that this gene is upregulated in tumors resistant to antifolates^71^. Finally, as FP-RMS Patient Derived Xenografts (PDX) better model the cellular heterogeneity of human tumor compared to cell line derived xenografts^72^, we treated five mice bearing established PDX tumors derived from the SJRHB031117_X1^73^ with pralatrexate (60 mg/kg, 2×/week for 2 weeks) or vehicle control and monitored tumor volume for four weeks or until endpoint. In all cases, pralatrexate inhibited tumor growth and in some cases even caused tumor regression (Fig. 9J, K) with no apparent weight loss, indicating limited toxicity. These findings support repurposing pralatrexate for the treatment of FP-RMS driven by different oncofusions.

### Combinations that improve pralatrexate efficacy

**Fig. 10:**
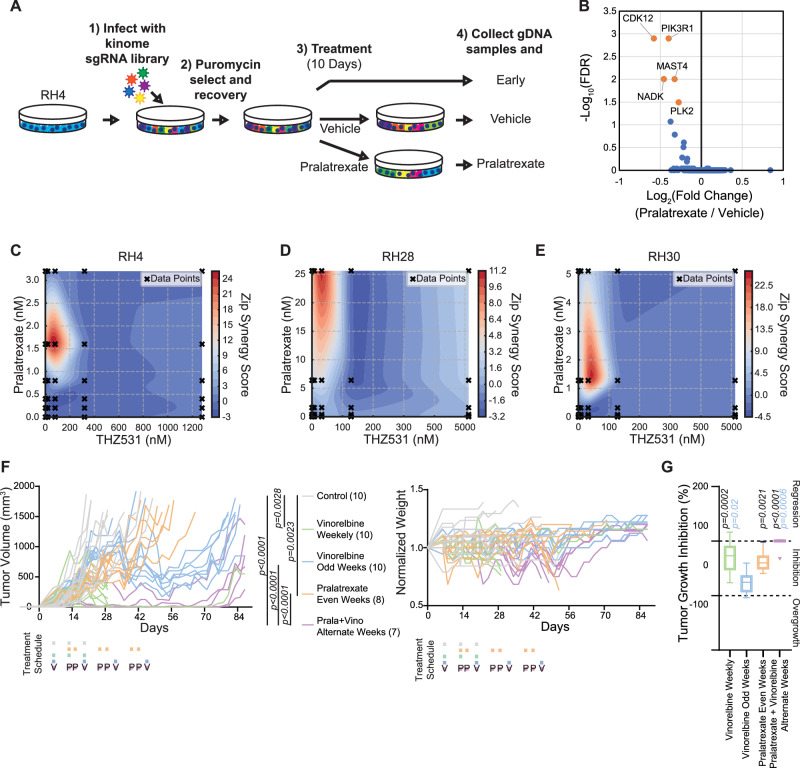
Combination therapy sensitizes FP-RMS cells to pralatrexate. **A** Schematic of the kinome-targeted CRISPR/Cas9 screen to identify synergistic therapeutic targets for pralatrexate treatment. **B** Volcano plot of the log_2_ fold change versus the -log_10_ false discovery rate (FDR) representing differential gene scores comparing RH4 cells transduced with a sgRNA library targeting the kinome and treated for 10 days with 1.5 nM pralatrexate or DMSO control (*n* = 3 technical replicates per condition. Orange:FDR < 0.05). **C**–**E** Gaussian smoothed topographical heat map of Zip synergy scores for (**C**) RH4, (**D**) RH28, and (**E**) RH30 cells treated with THZ531 and pralatrexate. (*n* = 3 per condition). Dose response matrix results from which the synergy scores were calculated are reported in Supplementary Fig. 12B–D. **F** Tumor volume (*left*), normalized animal weight (*right*), and drug schedule (*bottom*) versus time of mice (*n* = 7–10) bearing subcutaneous xenograft tumors derived from RH28 cells treated with the indicated drugs or a vehicle control. (mixed effect model, F-tests (Type III)). **G** Box and whisker plot of tumor growth inhibition of mice bearing subcutaneous xenograft tumors derived from RH28 cells treated with the indicated drugs. Center line: median. Box: 25^th^ to 75^th^ percentiles. Whisker: 75^th^ or 25^th^ percentile plus or minus 1.5x interquartile range. (one-way ANOVA; *n* is annotated in the related panel (**F**). Source data are provided as a Source Data file.

While FP-RMS tumors were sensitive to pralatrexate, they persisted or recurred following treatment withdrawal. As combination therapy is a common strategy to overcome chemotherapeutic resistance^74^, we employed two independent approaches to identify therapeutics to combine with pralatrexate. First, kinases are highly tractable targets, with at least 80 drugs targeting 22 kinases currently approved by the FDA^75^. Given this, triplicate cultures of RH4 cells were infected with a lentiviral sgRNA library targeting 763 genes encoding kinases or their regulatory subunits^76^, and after seven days of puromycin selection, a portion of these cells was harvested as an early time point control, while the remaining cells were treated with vehicle or 1.5 nM pralatrexate for 10 days. Genomic DNA was extracted, and sgRNA sequences barcoded, amplified, and sequenced (Fig. 10A). Comparing normalized sgRNA counts between pralatrexate- and vehicle-treated samples identified sgRNAs targeting *CDK12, PIK3R1, NADK, MAST4*, and *PLK2* as disproportionately depleted in the pralatrexate-treated cells (Fig. 10B; Suppl. Dataset 14), but having little to no effect in the absence of pralatrexate (Suppl. Fig. 12A). As CDK12 was the top hit from this screen and has been pharmacologically targeted with the compound THZ531^77^, we performed combination dose-response assays in RH4, RH28, and RH30 cells in triplicate with eight different doses of each. Derived ZIP synergy scores^78^ revealed subtherapeutic concentrations of THZ531 are synergistic at the IC_50_ of pralatrexate in all three cell lines (Fig. 10C–E; Suppl. Fig. 12B–D). These findings highlight CDK12 inhibition as a promising strategy to enhance pralatrexate efficacy in FP-RMS.

Second, vinorelbine is used as a second-line therapy for FP-RMS patients with recalcitrant disease^79,80^. As such, the most likely entry point for evaluating pralatrexate for the treatment of FP-RMS would be in combination with vinorelbine. As preliminary studies revealed toxicities when these two drugs were administered at the same time, we instead treated mice at previously published maximum tolerated doses (pralatrexate @ 60 mg/kg, 2×/week, vinorelbine @ 10 mg/kg, 1×/week), but alternating their weekly administration for 10 weeks or until endpoint. As controls, we included cohorts of mice treated with vehicle, vinorelbine alone on odd weeks, pralatrexate alone on even weeks, and vinorelbine weekly. To effectively test if the combination improved upon monotherapy, xenograft tumors were derived from the RH28 cell line, as pralatrexate inhibits but does not lead to regression of these tumors, and therapy began once tumors were palpable. As expected^79,80^, weekly vinorelbine treatment inhibited tumor growth in comparison to the vehicle, inducing complete regression in one tumor, but at the cost of significant toxicity in all mice after the third dose that prevented further analysis of all but one mouse (Fig. 10F). The three other treatments also led to weight loss by seven weeks, and hence all treatments were halted at that point. Bi-weekly vinorelbine treatment caused regression in six of ten tumors, but with a substantial delay, with all tumors beginning to grow after treatments ceased. Pralatrexate caused significant tumor growth inhibition compared to vehicle, with all tumors reaching maximum tumor volume by 60 days (Fig. 10F), similar to that observed previously (Fig. 9E), but now at a reduced treatment schedule. The combination therapy, however, led to regression in all seven tumors, and when treatments ceased, tumors took a protracted period of time to resume growth. The percentage of tumor growth inhibition was calculated for each drug at the time the first tumor in vehicle-control mice reached the tumor size end-point. This revealed that all but the odd-week treatment with vinorelbine led to significant tumor growth inhibition (Fig. 10G), although at later time points this treatment did lead to transient tumor regression (Fig. 10F). However, of all the therapies, only the combination therapy led to significant tumor regression (Fig. 10G). Thus, the combination therapy was superior to the other tested therapies, and with less toxicity than second line therapy vinorelbine at the same weekly dosing schedule. Collectively we suggest pralatrexate and vinorelbine is an immediately actionable combination to evaluate in the second line setting of FP-RMS, while the pralatrexate and CDK12 inhibition offers a potential future second combination therapy.

## Discussion

There is a growing number of translocations being identified in histologically similar tumors that are consistent with an RMS diagnosis^9,17–21^, which are known or predicted to encode proteins comprised of the N-terminus of PAX3 or its paralog PAX7, but with very different C-terminal fusion partners. This raises the question of how such divergent C-terminal partners impart similar oncogenic activity. To this end, first, we demonstrate that these different oncofusions are functionally interchangeable, engage a common interactome, bind to similar genes involved in RMS, and drive a common core transcriptional program. Related, different oncofusions involving the same NUP98 N-terminal fusion driving leukemia were also reported to similarly share a common interactome^81^. Second, we find that the full-length C-terminal fusion partners, or their paralogs, are components of the common interactome and cohabitate genes with the oncofusion. A parallel example is the AFF4 protein, whereby the C-terminal partner in the leukemia MLL::AFF4 oncofusion co-immunoprecipitated with other MLL fusion proteins^82^. We thus propose that FP-RMS oncofusions arise from PAX3/7 fused to functionally distinct C-terminal fusion partners found within a shared set of core oncofusion-protein interactions. We further suggest that each oncofusion therefore functions by localizing the common interactome to shared genomic loci, driving a common oncogenic transcriptional program (Fig. 2A).

One prediction of this model is that essentially any component of the common interactome could, at least in principle, be a candidate C-terminal fusion partner. Indeed, a recently described RMS case^49,52,83^ reported a *PAX3::WWTR1* fusion transcript, and we identified WWTR1 in the common interactome. Consistent with this prediction, we found that the encoded P3W fusion protein is expressed and regulates the same transcriptional pathways as those of P3F1. Currently, in the United States, diagnosis of FP-RMS is determined by Archer fusion sequencing or fluorescent in situ hybridization using fusion-specific probes, and hence will not detect rare or yet-to-be-discovered fusions. As such, comprehensive sequencing-based approach informed by the common interactome could be used to detect rare and previously undescribed RMS oncofusions that may better represent the molecular underpinnings of the tumor, and in turn, better stratify patients for treatment protocols.

The common interactome may not, however, predict all possible oncofusions. For instance, since PAX3 or PAX7 promoters drive transcription of the oncofusion, the C-terminal partner may not need to be expressed and could be absent from the common interactome. However, such partners would likely still interact with common interactome proteins. For example, while MAML3 of P3M was neither expressed in RH4-flag cells nor detected in other oncofusion interactomes, its paralog MAML1 is a component of the common interactome. Similarly, a *PAX3::MYOCD* translocation was recently reported in RMS^84^, and while MYOCD was neither expressed nor detected in the common interactome, its paralog MRTFB^85^ was identified in P3F1 and P7F1 interactomes. Additionally, not all common interactome genes may recombine with *PAX3* or *PAX7* due to genome-wide variations in recombination rates^86^. Likewise, FP-RMS cell lines are sensitive to expression levels of the oncofusion^39^, which may influence candidate fusion partners. Other factors, such as the ability to recruit the entire interactome, protein size, disruptive activities, heterozygous loss of function, a suitable breakpoint that retains the correct reading frame, or unaccounted features, may also limit fusion partner candidacy.

Interestingly, components of the common interactome are also found in other oncofusions. For example, NCOA2 of P3N2 is fused to MEIS1 in renal sarcomas and to ESR1 or GREB1 in ovarian sex-cord tumors, while MAML3 of P3M is fused to BCOR in sarcomas and ETV6 in salivary gland carcinomas^42,87–90^. The common interactome proteins ASXL1, AUTS2, and NCOR1 are also fusion partners with PAX5 in B-cell leukemia^23,91^. Cross-referencing the common interactome with catalogues of fusion genes reported across cancers (ChimerSeq+^92^, FusionGDB2^93–95^, and FOdb-II^96^ datasets) revealed enrichment of common interactome genes in both N- and C-terminal positions, even when restricting the fusions to transcription factors (Suppl. Fig. 13), suggesting shared mechanisms of transcriptional regulation across cancers.

The common interactome also offers a rich pool of therapeutic targets independent of the initiating oncofusion. Indeed, we show that the common interactome is enriched in FP-RMS dependencies, and that targeted inactivation of such components can disrupt oncofusion-driven transcription. A number of ubiquitin ligases comprise the common interactome (Suppl. Fig. 5; Suppl. Dataset 6), which may provide a future avenue to target the oncofusions^97^. However, the common interactome also contained more immediately actionable targets as well. Case in point, the folate-dependent enzyme TYMS is a component of the common interactome, an FP-RMS–selective vulnerability, and — like other common interactome members — supports the oncogenic transcriptional program of P3F1. These findings align with recent reports that methotrexate perturbs the P3F1 transcriptional program^98^ and that ectopic expression of human TYMS in mice promotes spontaneous sarcoma formation, including rhabdomyosarcoma, in the absence of the *Ink4a/Arf* tumor suppressor^99^.

Taken together, these data provided a strong rationale to evaluate antifolates and 5-FU in FP-RMS. Indeed, we found that pralatrexate induced complete regression in one FP-RMS cell-derived tumor model, significantly inhibited tumor growth in two others, and drove regression in a patient-derived xenograft. In agreement, methotrexate was shown to inhibit FP-RMS xenograft tumor growth^98^, and in a small pediatric sarcoma case study of 5-FU that included two RMS patients, one of these patients showed reduced marrow tumor burden, and the other achieved a complete response^100^. Furthermore, a phase II trial administering high-dose methotrexate up-front in adolescents with high-risk or metastatic RMS reported a ~30% response rate, with three of five aRMS patients achieving stable disease or partial responses^101^. Given that we found that pralatrexate outperformed 5-FU and other antifolates in vitro and in vivo, and has never been evaluated in FP-RMS patients, it is reasonable to predict superior clinical benefit relative to 5-FU or methotrexate.

While these data support clinical evaluation of pralatrexate, pralatrexate alone may not evoke a complete response. In this regard, we performed a kinome-targeted CRISPR/Cas9 screen that identified CDK12 as synergistic with pralatrexate, and further validated this synergy with a tool CDK12 inhibitor, and demonstrate that pralatrexate enhanced the antineoplastic activity of second-line therapy vinorelbine and reduced the cumulative toxic effects of vinorelbine by prolonging the time between doses. These findings suggest multiple potential paths to develop pralatrexate as a therapy for FP-RMS.

Although pralatrexate was antineoplastic regardless of the driving oncofusion, we note that principal component analysis revealed that the interactomes and transcriptomes of different oncofusions are largely distinct. As such, proteins uniquely associating with specific oncofusions may provide fusion-specific therapeutic targets. For example, C-terminal fusions appear to confer varying levels of oncogenic activity. This is supported by findings that patients with the P7F1 oncofusion tend to have better prognoses than those with P3F1 and in many cases go unrecognized as FP-RMS^3,102^, an observation currently being evaluated in the COG study ARST1431 (NCT02567435). Consistent with this, we observed slower cell growth in P7F1- and P3I-rescued cells, along with notable variability in fusion interactomes and transcriptional programming. Indeed, the CRISPR screen targeting all identified oncofusion interactomes in four cell lines driven by different oncofusions revealed oncofusion-specific vulnerabilities with interesting biological and clinical contexts such as differences in acetylation, ubiquitin ligase, and other dependencies (Fig. 6D; Suppl. Fig. 8B). Thus, there may also be oncofusion-specific vulnerabilities that could be exploited.

In summary, we report that the different oncofusions driving FP-RMS share a common core interactome, which we theorize renders them fungible. As this common interactome contains all the known full-length C-terminal fusion partners or their paralogs, we further suggest that oncofusions in RMS arise from the region of *PAX3* or P*AX7* encoding a common DNA-binding domain fused to genes encoding proteins within this common interactome. Finally, as all seven oncofusions engage the same core set of proteins, this common interactome could provide a way to target the oncogenic function of oncofusions, regardless of the C-terminal fusion partner; case-in-point being TYMS.

## Methods

### Patient sample collection and ethics statement

This research complies with all relevant ethical regulations, including informed patient consent. In relation to rhabdomyosarcoma patient-derived xenograft SJRHB03117_X, excess, de-identified tumour material was collected from patients with solid tumours at St. Jude Children’s Research Hospital in agreement with local institutional ethical regulations and institutional review board approval. Patient consent for tissue acquisition was obtained under the guidelines of the MAST protocol (Title: Molecular Analysis of Solid Tumors; St Jude's IRB Number: Pro00001240; Mnemonic: XPD09-234 MAST (NCT01050296); IRB Approval Date: 3/15/22).

RMS000EEC tumoroids and patient tumor samples were obtained via an established tumor sample acquisition route from patients treated at the Emma Children’s Hospital Amsterdam (Amsterdam UMC; RMS006, RMS007, RMS013) or as part of the biobank initiative of the Princess Maxima Center for Pediatric Oncology, Utrecht, the Netherlands (PMC; remaining tumor samples). Ethics approval was granted for the biobanking initiative, and the PMC biobank committee granted approval for this project (PMCLAB2018-009). All patients and/or their legal representatives signed informed consent to have tumor samples taken for biobank usage. Experiments conformed to the principles set out in the WMA Declaration of Helsinki and the Department of Health and Human Services Belmont Report.

### Mouse models

All mouse care and experiments were performed in accordance with a protocol approved by the Institutional Animal Care and Use Committee (IACUC) of Duke University (protocol no. A143-22-08). All mice were housed in the Duke University Division of Laboratory Animal Resources (DLAR) facility under a 12 h light/12 h dark cycle at an ambient temperature of 20–26 °C and relative humidity of 30–70%. Fox Chase SCID/*beige* mice (CB17.Cg-PrkdcscidLystbg-J/Crl, Charles River) were used in all studies. Experiments began with mice within 6–8 weeks of age and humanely euthanized before 6 months by CO_2_ asphyxiation with secondary euthanasia performed by decapitation, after which tumors were removed during necropsy. Maximum tumor burden approved by IACUC was 1.5 cm^3^ and this was not exceeded.

### Cell culture

#### Adherent culture

RH4 cells engineered to express a C-terminal flag-tagged P3F1 from the endogenous locus^32^, termed RH4-flag here for ease of description (a kind gift from Dr. Beat Schäfer) were cultured in RPMI supplemented with 10% FBS at 37 °C and 5% CO_2_. RH4, RH28, RH30, and CW9019 cells were STR profiled and cultured as above. 293T cells used to produce lentivirus were cultured in DMEM supplemented with 10% FBS at 37 °C and 5% CO_2_. To induce *shRNA* expression, cells were treated with 1 µg/ml doxycycline (MilliporeSigma). All stable cell lines generated by lentiviral transduction are available upon request.

#### Tumoroid culture

RMS000EEC tumoroids were cultured as previously described^52^. Briefly, cultures from early passage cryovials were restarted at high density (50,000 cells/cm^2^) in BM1* medium supplemented with 0.3% BME. Cells were grown at 37 °C and 5% CO_2_ as attached cells with a doubling time of approximately 144 h and passaged at an initial density of 25,000 cells/cm^2^.

### Vectors

EZ-Tet-pLKO-Blast was a gift from Dr. Cindy Miranti (Addgene plasmid # 85973; http://n2t.net/addgene:85973; RRID:Addgene_85973)^103^. pMT025 was a gift from Drs. John Doench and David Root (Addgene plasmid # 158579; http://n2t.net/addgene:158579; RRID:Addgene _158579)^104^. C1(1-29)-miniTurbo-V5_pLX304 was a gift from Dr. Alice Ting (Addgene plasmid # 107176; http://n2t.net/addgene:107176; RRID:Addgene_107176)^35^.

### Cloning

#### shRNA

An shRNA targeting the *P3F1* breakpoint (*shP3F1*; sequence: 5′-CCTCTCACCTCA GAATTCATT-3′) was designed based on prior published work^7^, but adapted to extend further into the *FOXO1* sequence and with one missense mutation to limit off-target effects. Cloning these sequences into EZ-Tet-pLKO-Blast was performed as per a published protocol^103^.

#### TurboID vectors

To generate pMT025-TurboV5-NLS, the miniTurbo-V5 sequence was subcloned from C1(1-29)-miniTurbo-V5_pLX304 into pMT025 by first PCR amplification with Q5 polymerase (NEB) with the primers 5′-TTCAGGTGTCGTGAGGCTAGCGCCA CCATGATCCCGCTGCTGAACGCTAAAC-3′ and 5′-CGCGTACTAGTCCCGGGATCCTCAG TCCAGCTTCGCCCTCTTGGCGGCCGGGGTGCTGTCCAGGCCCAGC-3′, which incorporate an NheI restriction site at the 5′ end and an NLS sequence followed by a BamHI restriction site at the 5′ end of the amplicon. The amplicon and pMT025 were then digested using NheI and BamHI restriction enzymes (NEB), ligated, and transformed into Stbl3 *E. coli*. The plasmid sequence was verified by Sanger sequencing through the open-reading frame.

pMT025-P3F1-, P3F4-, P3N1-, P3N2-, P3M-, P3I-, P7F1-, FOXO1-, EP3-, and EF-TurboV5 were created by cloning the cDNA encoding the desired oncofusions^9,17–21^ with the miniTurbo-V5 sequence added in-frame at the 3′ end (generated by gene synthesis, GenScript Biotech), into the NheI and BamHI sites of pMT025. To create transcripts generated by pMT025-P3F1-TurboV5 resistant to the shRNA targeting endogenous *P3F1* gene, the below four nucleotide changes were made to the cDNA sequence surrounding the fusion breakpoint.

*PAX3::FOXO1*: 5′-cctctcacctcagaattcaatt-3′

*PAX3::FOXO1* shRNA resistant: 5′-cctctcTccAcagaaCtcTatt-3′

#### Plasmid availability

The following plasmids generated in this study have been deposited with Addgene:

#218804EZ-Tet-pLKO-Blast - shP3F1

#218805EZ-Tet-pLKO-Blast - shScr

#218806pMT025-P3F1-TurboV5

#218807pMT025-P3F4-TurboV5

#218808pMT025-P7F1-TurboV5

#218809pMT025-P3N1-TurboV5

#218810pMT025-P3N2-TurboV5

#218811pMT025-P3I-TurboV5

#218812pMT025-P3M-TurboV5

#218813pMT025-TurboV5-NLS

#221083pMT025-EP3-TurboV5

#221084pMT025-EF-TurboV5

#### Individual sgRNAs

sgRNA sequences were encoded in oligos with 5′ and 3′ adaptor sequences (IDT) as described in Suppl. Dataset 15. 100 µM oligos were diluted 1:10 in water and amplified using Phusion Hotstart Flex master mix (New England Biolabs Inc) with the ArrayF and ArrayR primers (IDT) as follows:

ArrayF-

5′-TAACTTGAAAGTATTTCGATTTCTTGGCTTTATATATCTTGTGGAAAGGACGAAACA CCG-3′

ArrayR-

5′-ACTTTTTCAAGTTGATAACGGACTAGCCTTATTTTAACTTGCTATTTCTAGCTCTAAA AC-3′

sgRNA amplicons were cloned into lentiCRISPR v2. 5 µg of the lentiCRISPR v2 vector was digested with FastDigest Esp3I (Thermo Fisher Scientific) enzyme and electrophoresed on a 1% agarose gel. The ~11 kb was excised from the gel and purified using a QIAquick Gel Extraction Kit according to the manufacturer’s instructions (Qiagen). The amplified library was assembled into the restriction-digested vector using Gibson Assembly Master Mix (New England Biolabs Inc.) and transformed in Stbl3 competent E. coli (Thermo Fischer).

### Lentivirus production

293T cells were cultured to 70 to 80 % confluency and transfected with lentiviral packaging system at the ratio of 1:1:2 for psPAX2:pVSVg: Packaging Plasmid using Fugene6 (Promega) transfection reagent. Transfected cells were washed with 1x *P*hosphate *B*uffered *S*aline (PBS: 0.137 M NaCl, 0.0027 M KCl, 0.01 M Na_2_HPO_4_, and 0.0018 M KH_2_PO_4_) and the media was changed 4 to 6 h later. 48 to 72 h after transfection, media was harvested from cells, centrifuged at 400 *g* for 3 min and filtered using a 45 µm pore filter (VWR) to remove cell debris. Virus was either used immediately or snap frozen for future use.

### Infection

Cells were infected in a 6-well plate in 2 to 3 ml per well of RPMI media containing virus with of 8 µg/ml polybrene (Millipore Sigma) and spun 265 *g* for 1 h. After 4 to 6 hat 37 °C, the cells were washed in 1x PBS and the media was replaced with RPMI supplemented with 10% FBS. 18 h later, each infection was pooled into a 10 cm culture dish and selected with 10 µg/ml blasticidin for at least 7 days or 2 µg/ml puromycin for at least 48 hours.

### *siRNA* sequences and transfection

*siRNA* sequences were ordered from Integrated DNA Technologies as 27mer duplex siRNAs. Double-stranded NC1 duplex (51-01-14-03) was used as a negative control for this experiment. For the *PAX3::WWTR1* fusion, a custom design was made spanning the fusion breakpoint:

CD.Ri.228178.13.1-SEQ1:

5′-rCrCrUrCrUrCrArCrCrUrCrArGrArArGrArUrGrArArUrCCG-3′

CD.Ri.228178.13.1-SEQ2:

5′-rCrGrGrArUrUrCrArUrCrUrUrCrUrGrArGrGrUrGrArGrArGrGrCrC-3′

*siRNA* transfections were performed by harvesting RMS000EEC cells and re-plating them in 24-well plates at 200,000 cells per well in 500 µl BM1* supplemented with 0.3% BME. After 24 hours, transfections were performed by adding a mixture of 50 µl of Lipofectamine RNAiMAX (ThermoFisher Scientific 13778100) and DsiRNA in OptiMEM (ThermoFisher Scientific 31985062) to each well, so that the final concentrations in the well were 3 µl LF/ml and 30 nM DsiRNA. Medium was removed and cells were washed after 24 h and then harvested after 48 h. For RNA isolations, two wells were washed briefly with ice-cold PBS and cells were lysed directly in TRIzol (ThermoFisher Scientific 15596026). For immunoblot analysis, two wells were washed with PBS and cells were lysed directly in 2xLaemmli sample buffer with β-mercaptoethanol.

### Growth assays

#### Long term (13 days)

1;× 10^5^ cells were plated per well of a 6-well plate, and where appropriate, induced with doxycycline. Cells were imaged and counted using a Countess II cell counter (Thermo Fischer Scientific) on days 2, 6, and 13 and split into subcultures at a 1:5 ratio on days 4, 7, and 11. Cell counts were adjusted based on the number of splits. To determine the average doubling times, a model of exponential growth was fit to the cell counts of individual wells over time using PRISM statistical software (GraphPad, ver. 10.0.3 (275)) and averaged across replicates. Statistical analysis of average doubling times was also carried out with the same PRISM software using a one-way ANOVA analysis.

#### Short term (96 hours)

1 × 10^5^ cells were plated per well of a 12-well, and where appropriate, induced with doxycycline. One set of replicate wells were inoculated for each time point. 3, 48, and 96 hours later, cells were fixed in 10% neutral buffered formalin and stained with 0.5% crystal violet (MilliporeSigma). Plates were then rinsed three times with distilled water and allowed to dry completely. Dried plates were imaged on a flatbed scanner and analyzed by measuring absorbance at 590 nm in a 6 × 6 circle for each well using a Tecan plate reader. Readings in each well were averaged and values obtained from an empty well were subtracted as background. Like with the long-term growth assays, exponential growth curves were fit to absorbance values over time to determine and compare average doubling times.

#### Colony formation

A total of 400 cells were plated in 3 wells of a 6 well plate. The media was changed every 3 days and cells were cultured for 14 days total. cells were fixed in 10% neutral buffered formalin and stained with 0.5% crystal violet (Millipore Sigma). Plates were then rinsed three times with distilled water and allowed to dry completely. Dried plates were imaged on a flatbed scanner and analyzed by counting the number of colonies visible to the naked eye.

#### Hanging droplet spheroid growth

Methylcellulose stock media is necessary to efficiently generate spheroids. Stocks were made by autoclaving 1.2 *g* of 4000 cp methylcellulose with a magnetic stir-bar in 100 ml glass bottle. 50 ml base media heated to 60 °C was added to cooled methylcellulose and stirred for 30 min. 40 ml of base media, 10 ml FBS were added and further supplemented with penicillin (100 U/ml), and streptomycin (100 mg/ml). Stock media was stirred for 16 hours at 4 °C. Media was then moved to two 50 ml conical tubes and centrifuged at 800 g for 60 min. 10 ml aliquots were frozen at −20 °C. 1 × 10^4^ cells were resuspended in 125 µl of methylcellulose stock media supplemented with 1 µg/ml doxycycline and brought to 1 ml total volume with DMEM supplemented with 10% FBS and doxycycline. Each spheroid was formed by 20 µl (~200 cells) in 1 micro-well of a 60 micro-well plate (Nunc). Spheroids were cultivated by inverting the plate in a humidity chamber at 37 °C and 5% CO_2_. In most wells an individual spheroid formed at the media-air interface after 48 hours. Only wells exhibiting spheroids were analyzed. Every 3 days, 10 µl of DMEM supplemented with 10% FBS and doxycycline was added to each well. Spheroids were imaged 9 days after initial plating. Spheroids were imaged using an EVOS m5000 (Thermo Fisher Scientific) equipped with a 10x objective and camera. Spheroid area was measured in ImageJ (v 1.53t) by manually outlining each spheroid using a stylus.

#### Growth assays following sgRNA transduction

Cells were infected with lentivirus encoding sgRNA and Cas9. 24 hours after infection cells were selected with 2 µg/ml puromycin for 72 hours. 7 days after infection, 4 × 10^4^ cells were plated in three wells of a 12-well plate and incubated for 7 days with one media change at day 4. Cells were then fixed, crystal violet stained and analyzed as above.

### Immunoblot

#### Cultured cells

Cells in plates were washed three times in 1x PBS and then lysed by scraping cells in Radioimmunoprecipitation Assay (RIPA, Tris Chloride, 10 mM; EDTA 1 mM; EGTA, 0.5 mM; Triton X-100, 1%; Sodium Deoxycholate, 0.1%; SDS, 0.1%; Sodium Chloride, 140 mM) buffer supplemented with Complete Protease Inhibitor Cocktail (Roche) and 50 mM sodium fluoride. Lysates were rotated at 4 °C for 30 min and centrifuged at 4 °C for 15 min at 21,000 *g* to clear lysate. Protein concentration of lysates was assayed by Bicinchoninic Acid solution assay (Millipore Sigma) and read on a Glomax Multidetection System (Promega). Lysates were resolved by SDS-PAGE, transferred to a PVDF membrane (Bio-Rad, 1704273), blocked in 5% milk in Tris-buffered saline with Tween (TBST: 20 mM Tris, 150 mM NaCl, and 0.1% (v/v) Tween-20 detergent), or for Streptavidin probing, blocked in 5% BSA (MilliporeSigma) in TBST, followed by immunoblot with the following antibodies in 5% milk in TBST or 5% BSA in TBST: V5 (Thermo Fisher Scientific, R960-25, 1:1,000), flag (Millipore Sigma, f1804, 1:1000), FOXO1 (Cell Signaling, 2880S, 1:1,000), Streptavidin-HRP (Thermo Fisher Scientific, SA10001, 1:5,000), GAPDH (Santa Cruz, sc-365062, 1:1,000), NCOA1 (Abclonal, A9058, 1:500), NCOA2(Abclonal, A10280, 1:500), INO80D(Millipore Sigma, HPA043976, 1:500), MAML1 (Cell Signaling, 12166, 1:500), TYMS (Cell Signaling, 5449, 1:1,000), DHFR (Cell Signaling, 45710, 1:1,000) and ß-Tubulin (Sigma, T5201, 1:5,000). Primary antibody incubation was performed at 4 °C overnight followed by the secondary antibody incubation for 1 h at room temperature. FIJI (v1.54 f)was used for densitometry analysis of immunoblots. Standard sized regions of interest (ROI) were analyzed for each band across samples as well as an ROI on a region of the membrane not containing samples for background subtraction. Integrated intensity was measured for each ROI and background was subtracted for each sample. The protein of interest was then compared to the loading control (protein/loading control) and all values were normalized to the mean value for control samples resulting in a normalized expression value with arbitrary units.

#### Tumors

30 to 50 mg of a tumor were lysed on ice with a mechanical tissue homogenizer in 500 µl RIPA supplemented with protease inhibitors. All lysates were rotated at 4 °C for 30 min and centrifuged at 4 °C for 15 min at 21,000 *g* to generate clear lysates for immunoblot analysis.

#### Tumoroid cultured cells

RMS000EEC tumoroid cells were collected and washed in PBS, and subsequently lysed in 2xLaemmli buffer with β-mercaptoethanol at 2 × 10^6^ cells/ml. A stain-free kit was used to cast 10% SDS-PAGE gels and blotting was performed by using a TurboBlot device (BioRad). Antibodies used were TAZ (E9J5A) (Cell Signaling Technology, #72804) and GAPDH (Abcam, ab9485), both at 1 in 1000 dilutions. For detection, Goat-anti-Rabbit-coupled HRP was used 1:10,000, followed by ECL detection. Experiments were performed in duplicate. ImageJ was used for quantification by normalizing to the GAPDH loading control.

### Immunofluorescence microscopy

Cells were plated on glass coverslips for 24 hours. Cells were then fixed in ice-cold 4% formaldehyde in PBS for 5 min on ice, permeabilized with 0.1% Triton-X100 in PBS for 5 min at room temperature, washed three times in PBS and blocked in 5% BSA in PBS for 1 hour at room temperature. Coverslips were then incubated with the V5 (Thermo Fischer Scientific; 1:500) primary antibody diluted in 5% BSA in PBS and incubated at 4 °C for 16 h. Coverslips were then washed three times in PBS and incubated in alexafluor-555 conjugated goat anti-mouse antibody (Thermo Fischer Scientific, 1:500), alexafluor-488 conjugated streptavidin (Thermo Fischer Scientific, 1:1,000), and 4′,6-diamidino-2-phenylindole (DAPI) (Millipore Sigma; 1:1,000) diluted in 5% BSA in PBS for 1 hour at room temperature. Coverslips were washed three times in PBS and mounted onto a slide using Fluoromount G (Thermo Fischer Scientific). Phase contrast micrographs of these slides were captured a Leica binocular inverted microscope equipped with a 10x objective and camera. Fluorescence and phase contrast micrographs were captured using an Evos M5000 epifluorescence microscope with RFP, GFP, and DAPI fluorescence filter sets and light sources.

### Proximity ligation assays

Proximity ligation assays were performed using the DuoLink In Situ Orange Starter Kit Mouse/Rabbit (Millipore Sigma) using V5 (Thermo Fisher Scientific, R960-25) and TYMS (Cell Signaling, 5449) antibodies at 1:500 in the supplied diluent. Fluorescence and phase contrast micrographs were captured using an Evos M5000 epifluorescence microscope equipped with RFP and DAPI fluorescence filter sets and light sources and an Invitrogen Plan FL PH 40x/0.65 RMS objective. Images were analyzed applying standardized thresholding values across images and particle analysis algorithms in FIJI (v1.54 f). PLA points/nucleus for each image was calculated by dividing the number of particles in the RFP channel by the number in the DAPI channel.

### Dose response and synergy assays

*A*ssays were carried out in 96 well plates with 2000 cells/well initially. 6 to 8 h after plating cells they were treated with 5-FU (Millipore Sigma), Raltitrexed (Selleckchem), Methotrexate (Selleckchem), Pemetrexed (Selleckchem), and/or Pralatrexate (Millipore Sigma) in a total volume per well of 100 µl. In the case of single-agent treatment, cells were incubated for 96 h and then the number of metabolically active cells was determined with the CellTiter-Glo reagent (Promega) following the manufacturer’s protocol. CellTiter-Glo luminescence was measured using a Promega Glomax multi-detection plate reader. For synergy assays, cells were incubated for 72 h. Luminescence values were normalized to the largest value equaling 100%.

### Xenografts

#### Genetically engineered cell lines

RH28 cells were transduced with lentiCRISPRv2 derived lentivirus and selected for 48 hours using 2 µg/ml puromycin. 1.5 × 10^6^ cells were transplanted into the right flank of 6–8 week old female Fox Chase SCID/*beige* mice. Mice were monitored three days a week and resultant tumor growth was measured using calipers to assess the length and width of the tumor. Tumor volume was estimated using the formula (Length * Width^2^)/2. Mice were monitored for 100 days or until the tumor volume reached 1,500 mm^3^, at which point the mouse was considered to have reached end-point and was humanely euthanized by CO_2_ asphyxiation followed by decapitation.

#### Single agent treatment

Cells were transplanted into the right flank of 6–8 week old female Fox Chase SCID/*beige* mice (Charles River Laboratories). For each transplant, 5 × 10^6^ cells were resuspended in 200 µl sterile PBS and kept on ice until injection (less than 2 hours). Mice were anesthetized in a bell jar with diffuse isoflurane, their injection site sterilized with iodine, and subcutaneously injected with 200 µl cell suspension in the right flank using a 30 G needle (BD). Tumor volume was monitored by calipers and the volume was estimated using the formula (Length * Width^2^)/2. Once tumor volumes reached 150 mm^3^ treatment began by intraperitoneal injection with i) 67 mg/kg 5-fluorouricil once per week for 10 weeks as previously described in ref. ^67^, ii) 7.5 mg/kg Raltitrexed daily for 5 days, then a drug holiday for 2 days, followed again by daily injections for 5 days, as previously described in ref. ^68^, iii) 60 mg/kg Pralatrexate twice weekly for 2 weeks as previously described in ref. ^69^, iv) 1 mg/kg Vincristine once a week for 10 weeks as previously described in ref. ^70^, and v) sterile PBS of at the same volume as vincristine once a week for 10 weeks as a control. Mice weight and tumor volumes were assessed every Monday, Wednesday, and Friday. If weight decreased by more than 15% from the initial value, the mouse was considered to have reached end-point. Once tumor volume reached 1500 mm^3^, the mouse was considered to have reached end-point and was humanely euthanized by CO_2_ asphyxiation followed by decapitation. Tumor growth inhibition was assessed by comparing individual inhibitor treated tumor volume to the mean vehicle control tumor volume at the point which the first vehicle control treated tumor reached end-point.

#### Combination treatments

Cells were transplanted and tumors were monitored as described for single agent therapy. Treatment regimens consisted of 5 arms with 10 mice per arm: i) PBS control same volume as Vinorelbine once a week for 10 weeks. ii) 60 mg/kg Pralatrexate 2x weekly (M, R) for 10 weeks on weeks 2,4,6,8 and 10. iii)10 mg/kg Vinorelbine 1x weekly (M) for 10 weeks. iv) 10 mg/kg Vinorelbine 1x weekly (M) for 10 weeks on weeks, 1,3,5,7 and 9. v) 60 mg/kg Pralatrexate 2x weekly (M, R) for 10 weeks on weeks 2,4,6,8 and 10 + 10 mg/kg Vinorelbine 1x weekly (M) for 10 weeks on weeks, 1,3,5,7 and 9.

#### PDX establishment

A rhabdomyosarcoma patient-derived xenograft (PDX) line SJRHB03117_X1 was obtained from St. Jude Children’s Research Hospital through the Childhood Solid Tumor Network (CSTN)^73,105^. CSTN PDX development pipeline have extensively described the PDX generation and propagation procedures for this model. The frozen vial of cells was thawed in a 37 °C water bath for 2 min and cells were transferred into a 15 ml conical tube containing 10 ml of DMEM supplemented with 10% FBS. The cells were centrifuged at 500 *g* for 5 min and resuspended at 6 × 10^6^ cells in 600 µl of cold Matrigel (Corning, Lot 32136002). For initial tumor establishment, 100 µl of the cell-Matrigel suspension was injected subcutaneously into both flanks of four SCID/*beige* mice using a 26-gauge needle. Mice were monitored until tumors reached a measurable size.

#### PDX tumor dissociation and secondary engraftment for treatment study

Once the primary tumors reached the predetermined harvest size, tumors were excised from the mouse. Tumors were dissociated using the St. Jude Rhabdomyosarcoma Tumor Dissociation Technique. Instead of using a tumor press as described in the CSTN protocol, tumors were mechanically minced into fine fragments using a sterile razor blade. All subsequent steps adhered to the established CSTN workflow without modification. Minced tissue was then subjected to an enzymatic dissociation including trypsin and collagenase, followed by an STI, DNase, and MgCl₂ treatment. Then the mixture was filtered through a 40 µm strainer and an RBC lysis step was performed. The single-cell suspensions were counted, pelleted, and re-suspended in cold Matrigel (Lot #24024002) at 1 × 10^6^ cells per 100 µl for reinjection. Ten SCID/*beige* mice (5 male and 5 female, Duke DLAR breeding core) received subcutaneous flank injections of 1 × 10^6^ cells in 100 µl of Matrigel. Once tumors reached approximately 250 mm³, mice were randomized to receive pralatrexate or vehicle control. 60 mg/kg of pralatrexate was administered intraperitoneally twice weekly for 2 weeks. Control animals received vehicle on the same schedule. Tumor volumes were measured thrice weekly using caliper measurements (volume = (length × width²)/2) and monitored for 4 weeks.

### CRISPR/Cas9 interactome screen

#### Library design

A custom sgRNA library (Suppl. Dataset 10) was designed to target the 2,073 genes encoding interacting proteins and 10 essential genes. 3 sgRNAs per target were designed using CRISPick^76,106^ with the following parameters; Reference Genome = Human GRCh38, Mechanism = CRISPRko, Enzyme = SpyoCas9, no-site controls = 15, Intergenic controls = 15.

#### Library cloning

Pooled oligos encoding sgRNA with 5′ and 3′ adaptor sequences (IDT) were diluted 1:10 in water and amplified using Phusion Hotstart Flex master mix (New England Biolabs Inc) with the ArrayF and ArrayR primers (IDT).

ArrayF: 5′-TAACTTGAAAGTATTTCGATTTCTTGGCTTTATATATCTTGTGGAAAGGACGAAAC ACCG-3′

ArrayR: 5′-ACTTTTTCAAGTTGATAACGGACTAGCCTTATTTTAACTTGCTATTTCTAGCTCT AAAAC-3′

sgRNA amplicons were cloned into lentiCRISPR v2-Hygro (a gift from Brett Stringer; Addgene plasmid #98291; http://n2t.net/addgene:98291; RRID:Addgene_98291)19. To prepare the vector, 5 µg of lentiCRISPR v2-Hygro was digested with FastDigest Esp3I (Thermo Fisher Scientific) and resolved on a 1% agarose gel. The ~11 kb band was excised and purified using the QIAquick Gel Extraction Kit following the manufacturer’s instructions (Qiagen). The amplified library was then assembled into the restriction-digested vector using Gibson Assembly Master Mix (New England Biolabs Inc.) and electroporated into library-competent *E. coli* (Lucigen Corp). Library plasmid DNA was isolated using the HiSpeed Plasmid Midi Kit according to the manufacturer’s instructions (Qiagen), and library coverage was confirmed by next-generation sequencing.

#### Lentivirus production

HEK293T cells were cultured to 70–80% confluency and transfected with the lentiviral packaging system at a 1:1:2 ratio of psPAX2:pVSVg:lentiCRISPR v2-hygro library using Fugene6 (Promega) transfection reagent. After 48–72 h, the media was harvested, centrifuged at 400 *g* for 3 min, and filtered through a 45 µm pore filter (VWR) to remove cellular debris. Virus was used immediately or snap frozen for future applications.

#### Cell infection

Cells were maintained in 2 µg/ml doxycycline to sustain P3F1 depletion throughout the screen. For each replicate, 1.2 × 10⁷ cells were infected at an MOI of 0.3 to ensure predominantly single-lentiviral integration events. Infections were performed across six wells of a 6-well plate, with 1 ml of the appropriate media containing virus at a predetermined concentration to achieve the target MOI, supplemented with 8 µg/ml polybrene (MilliporeSigma), and spun at 800 × *g* for 1 h. After 6 h of culture at 37 °C, cells were washed with PBS and the media was replaced. After 18 h, infected cells were pooled into a 15 cm culture dish and selected with 150 µg/ml hygromycin (Thermo Fisher Scientific). Cells were continuously passaged under selection for 1 week, after which 1 × 10^6^ cells were pelleted and snap frozen as the initial sample. An additional 1 × 10^6^ cells were maintained under continuous passage for 2 weeks as the last time point and subsequently pelleted and snap frozen.

#### gDNA isolation, sgRNA amplification, and sequencing

Cell pellets were lysed in 1 ml DNAzol by vigorous pipetting according to the manufacturer’s protocol (Molecular Research Center Inc.). DNA was precipitated in 0.5 ml 100% ethanol (VWR), followed by a second precipitation in 2.5 ml 100% ethanol. Precipitated DNA was pelleted by centrifugation at 20,000 *g* for 1 min, washed twice with 75% ethanol, and solubilized in 0.5 ml of water. To minimize PCR amplification bias, sgRNA sequences from each sample were amplified in triplicate and pooled at each step. First-round PCR was performed using 50–100 µg gDNA, Hotstart Taq (Takara), and 1 µM each of CRISPR F1 and CRISPR R1 primers (IDT):

CRISPR F1: 5′-AATGGACTATCATATGCTTACCGTAACTTGAAAGTATTTCG-3′

CRISPR R1: 5′-TCTACTATTCTTTCCCCTGCACTGTtgtgggcgatgtgcgctctg-3′

Thermal cycling conditions were 95 °C for 5 min; 18 cycles of 95 °C for 30 s, 60 °C for 30 s, 72 °C for 30 s; final extension at 72 °C for 5 min. Amplicons from the same sample were pooled, and a second PCR was performed to add sample barcodes and Illumina adaptors using 20 µl of PCR1 and 0.5 µM CRISPR R2 plus barcoded forward primers (Suppl. Dataset 16).

CRISPR R2: 5′-CAAGCAGAAGACGGCATACGAGATGTGACTGGAGTTCAGACG TGTGCTCTTCCGATCTtctactattctttcccctgcactgt-3′

Thermal cycling conditions were 95 °C for 5 min; 20 cycles of 95 °C for 15 s, 58 °C for 30 s, 72 °C for 30 s; final extension at 72 °C for 5 min. Second-round PCR products were pooled at equal concentrations and purified using Ampure XP Beads according to the manufacturer’s protocol (Beckman Coulter). Purified amplicons were sequenced on an Illumina MiSeq using 75 bp single-end reads.

### CRISPR/Cas9 kinome screen

RH4 cells were infected with lentivirus encoding the Brunello kinome library^76^ at 1,000x coverage. 48 h later, cells were selected with 0.25 µg/ml puromycin for 72 h. A portion of the cells were harvested at this point for baseline measurements. The remaining cells were allowed to recover for 72 h, and then treated with 1.5 nM pralatrexate or vehicle control and passaged at 4-day intervals for 10 days. On day 10, 5 × 10^6^ cells were harvested and genomic DNA isolated using an ammonium acetate precipitation method^107^. The sgRNA sequences from each sample were PCR amplified using primers with Illumina adaptors and indexes for multiplexing as described in ref. ^108^. Samples were pooled and sequenced at the Duke Genome Sequencing Core Facility using an Illumina Nextseq 1000, to an average sequencing depth of 1200 reads per sgRNA.

### Proximity labeling

#### Pulldown

Cells were switched to DMEM supplemented with 10% FBS for 2 weeks in order to reduce base line biotin concentration. Each sample condition was prepared in technical triplicate one time. Induced wells were also treated with doxycycline for greater than 2 weeks prior to biotin treatment. For each sample, ~70% confluent 15 cm dishes were treated with 50 µM biotin for 16 h before lysis in 500 µl RIPA buffer with Complete Protease Inhibitors (Roche). Lysates were rotated at 4 °C for 30 min and centrifuged at 4 °C for 15 min at 21,000 *g* to clear lysates. Protein concentration of lysates was determined as above. For each sample, 75 µl of Dynabeads MyOne Streptavidin C1 (Thermo Fischer Scientific) were equilibrated in 500 µl RIPA buffer with Complete Protease Inhibitors at 4 °C for 30 min while rotated end over end. Beads were magnetically separated from supernatant and incubated with 2.5 mg of lysate at 4 °C for 16 h with end over end rotation. Beads were again magnetically separated and washed three times in bead wash buffer (0.5% (w/v) sodium deoxycholate, 0.5% (w/v) igepal CA-630 (Millipore Sigma), 1 mM EDTA, 100 mM NaCl, 50 mM TrisHCl, pH 7.5). Beads were resuspended in 80 µl PBS, 20 µl 5x SDS loading dye (10 mM dithiothreitol, 0.02% Bromophenol Blue, 30% Glycerol, 10% sodium dodecyl sulfate and 250 mM Tris-Cl, pH 6.8) with 5 µM biotin and boiled for 10 min. Beads were magnetically separated as above and 50 µl of sample was used for QC immunoblot and Coomassie stained gel. The remaining 50 µl were analyzed by mass spectrometry.

#### Mass spectrometry

Samples were spiked with undigested bovine casein at a total of either 1 or 2 pmol as an internal quality control standard. Next, samples were reduced (10 mM dithiothreitol) for 30 min at 80 °C, alkylated with 20 mM iodoacetamide for 30 minutes at room temperature, then supplemented with a final concentration of 1.2% phosphoric acid and 485 µl of S-Trap (Protifi) binding buffer (90% MeOH/100 mM Triethylamine Buffer TEAB). Proteins were trapped on the S-Trap micro cartridge, digested using 20 ng/µl sequencing grade trypsin (Promega) for 1 hour at 47 °C, and eluted using 50 mM TEAB, followed by 0.2% formic acid, and lastly, using 50% acetonitrile/0.2% formic acid. All samples were then lyophilized to dryness. Samples were resolubilized using 12 µl of 1% trifluoroacetic acid/2% acetonitrile with 12.5 fmol/µl yeast alcohol dehydrogenase.

Quantitative liquid chromatography with tandem mass spectrometry (LC/MS/MS) was performed on 1 µl using an MClass UPLC system (Waters Corp) coupled to a Thermo Orbitrap Fusion Lumos high resolution accurate mass tandem mass spectrometer (Thermo Fischer Scientific) equipped with a FAIMSPro device via a nanoelectrospray ionization source. Briefly, the sample was first trapped on a Symmetry C18 20 mm × 180 µm trapping column (5 μl/min at 99.9/0.1 v/v water/acetonitrile), after which the analytical separation was performed using a 1.8 µm Acquity HSS T3 C18 75 µm × 250 mm column (Waters Corp.) with a 90-min linear gradient of 5 to 30% acetonitrile with 0.1% formic acid at a flow rate of 400 nanoliters/minute (nl/min) with a column temperature of 55 °C. Data collection on the Fusion Lumos mass spectrometer was performed for three different compensation voltages (−40 v, −60 v, −80 v). Within each CV, a data-dependent acquisition (DDA) mode of acquisition with a *r* = 120,000 (@ m/z 200) full MS scan from m/z 375–1500 with a target AGC value of 4 × 10^5^ ions was performed. MS/MS scans were acquired in the ion trap in Rapid mode with a target AGC value of 1 × 10^4^ and max fill time of 35 ms. The total cycle time for each CV was 0.66 s, with total cycle times of 2 s between like full MS scans. A 20 s dynamic exclusion was employed to increase depth of coverage. The total analysis cycle time for each injection was approximately 2 h.

Following UPLC-MS/MS analyses, data were imported into Proteome Discoverer 2.5 (Thermo Fischer Scientific). In addition to quantitative signal extraction, the MS/MS data was searched against the SwissProt H. sapiens database (downloaded in Nov 2019) and a common contaminant/spiked protein database (bovine albumin, bovine casein, yeast ADH, etc.), and an equal number of reversed-sequence “decoys” for false discovery rate determination. Sequest (v 2.5, Thermo PD) was utilized to produce fragment ion spectra and to perform the database searches. Database search parameters included fixed modification on Cys (carbamidomethyl) and variable modification on DPMSR Fusion Protein Rescue Met (oxidation). Search tolerances were 2 ppm precursor and 0.8 Da product ion with full trypsin enzyme rules. Peptide Validator and Protein FDR Validator nodes in Proteome Discoverer were used to annotate the data at a maximum 1% protein false discovery rate based on q-value calculations. Note that peptide homology was addressed using razor rules in which a peptide matched to multiple different proteins was exclusively assigned to the protein has more identified peptides. Protein homology was addressed by grouping proteins that had the same set of peptides to account for their identification. A master protein within a group was assigned based on % coverage. Prior to imputation, a filter was applied such that a peptide was removed if it was not measured in at least two unique samples (50% of a single group). After that filter, any missing data missing values were imputed using the following rules; (1) if only one single signal was missing within the group of three, an average of the other two values was used or (2) if two out of three signals were missing within the group of three, a randomized intensity within the bottom 2% of the detectable signals was used. To summarize to the protein level, all peptides belonging to the same protein were summed into a single intensity. These protein levels were then subjected to a normalization in which the top and bottom 10 % of the signals were excluded and the average of the remaining values was used to normalize across all samples. These data are presented in Suppl. Datasets 6–8.

### Sequencing

#### mRNA purification from cell culture

Except for cells transduced with *shP3F1* and Turbo-V5-NLS, each *RH4-flag* cell line was induced with doxycycline for more than 2 weeks before harvesting mRNA samples. Cells transduced with *shP3F1* and Turbo-V5-NLS were induced with doxycycline for 5 days before harvesting mRNA samples. For each sample, mRNA was isolated using RNeasy Plus Mini kits following the manufacturer’s instructions (Qiagen) from between 1 × 10^6^ and 1 × 10^7^ cells. Extracted total RNA quality and concentration were assessed on Fragment Analyzer (Agilent Technologies) and Qubit 2.0 (Thermo Fisher Scientific), respectively. All samples had an RQN > 7. RNA-seq libraries were prepared using the commercially available KAPA Stranded mRNA-Seq Kit (Roche) using 500 ng of total RNA for each sample. Illumina sequencing adapters were ligated to the output cDNA fragments and amplified to produce the final RNA-seq library with a dual-indexing approach for pooled library multiplex sequencing.

#### mRNA sequencing of cell culture samples

Before pooling and sequencing, each sub-library was assessed by a Fragment Analyzer (Agilent) and Qubit (Thermo Fisher Scientific) to determine fragment length distribution and Molarity. All libraries were then pooled in equimolar ratio and subjected to 100 bp paired-end sequencing by Illumina NovaSeq 6000 sequencer. RNA-seq data was processed using the fastp toolkit^109^ to trim low-quality bases and sequencing adapters from the 3′ end of reads, then mapped to GRCh38 (downloaded from Ensembl, version 106)^110^ using the STAR RNA-seq alignment tool^111^, and reads aligning to a single genomic location were summarized across genes. For genes having an overlap of at least 10 reads, gene counts were normalized and differential expression was carried out using the DESeq2^112^ Bioconductor^113^ package implemented for the R programming environment (R 4.3.0). Consistent with the recommendation of the DESeq authors, independent filtering^114^ was utilized prior to calculating adjusted *p*-values and moderated log2 fold-changes were derived using the ashr package^115^.

#### mRNA purification from tumoroid culture

RNA was isolated from the TRIzol samples as previously described in ref. ^52^. Briefly, after addition of 20% chloroform, the aqueous phase was used to isolate RNA using a Direct-zol RNA Miniprep kit (Zymo Research). The resulting RNA was tested by RT-qPCR for the P3W fusion mRNA abundance, and normalized to G6PD (primer sequences and methods in ref. ^52^. Fusion detection primers (product 192 bp):

*PAX3*-F_EEC:5′-AGCACCAGGCATGGATTT-3′

*WWTR1*-R_EEC:5′-TTCGAGGTCTGTGTCTAGGT-3′

#### mRNA sequencing of tumoroid culture samples

After confirming knockdown using RT-qPCR, RNA from both wells were pooled and tested for RNA quality by Bioanalyzer Bioanalyzer2100 RNA Nano 6000 chips (Agilent). Quantities were measured by Qubit. Strand specific mRNA libraries were prepared by Novogene (UK). RNA samples were sequenced using a paired-end approach with the Illumina NovaSeq 6000 platform at Novogene (UK) (PE150).

#### Cleavage Under Targets and Tagmentation (CUT&Tag)

CUT&Tag was performed as previously described^45^ using the commercial EpiCypher CUTANA (14-1102) kit and following the manufacturer’s detailed protocols. Briefly, 100,000 human tumor cells were used in each assay followed by sample preparation, multiplex library preparation, and deep sequencing (Illumina NextSeq 1000). The primary antibodies were used at 1:100 concentration: anti-Flag (Cell Signaling Technology, 8146T), anti-V5 tag (Abcam, 9116), anti-INO80D (Atlas Antibodies, HPA043976), anti-MAML1 (Cell Signaling Technology, 12166), anti-NCOA1 (ABclonal, A1128), anti-Rabbit (EpiCypher, 13-0047), and anti-Mouse (Abcam, 46540). Anti-Flag was used to probe endogenous PAX3::FOXO1 in parental RH4-flag cells and anti-V5 tag was used to probe the oncofusions in the *shP3F1* RH4-flag cells. After sequencing, Fastq files (trim_galore) were mapped to the reference genome (hg38) using bowtie2 (v.2.4.4). The non-primary alignment and PCR duplicates were removed from aligned data using Samtools (v.1.10) (-q 30 -F 1804 -f 2 for unique mapping reads), the Picard ‘MarkDuplicates’ function (v.2.18.2), and bedtools (v.2.30.0) (‘intersect’ function used to exclude genome blacklist regions), respectively. Peak calling was performed using MACS2 (v.2.2.6) (macs2 callpeak -f BAMPE -g hs/mm –keep-dup 1 --cutoff-analysis -q 0.05). For spike-in samples, fastq files were mapped to the reference genome using STAR (v.2.7.11a). Non-primary alignment and PCR duplicates alignments were removed by Samtools (v.1.10) (-F 256) and Picard ‘MarkDuplicates’ function (v.2.18.2). The scale factor used for normalization could be calculated by comparing spike-in alignments number among different groups. Motif analysis was performed by Hypergeometric Optimization of Motif Enrichment (HOMER)^116^. Deeptools (v3.3.0) was used to generate bigwig files, heatmaps and averaged plotting of CUT&RUN signals^117^. Genomic binding profiles were generated using the deepTools ‘bamCompare’ functions. The Genomic Regions Enrichment of Annotations Tool (GREAT)^118^ was used to determine enriched gene functions (McClean et al., 2010) and Integrative Genomics Viewer (IGV) was used to display and input normalized CUT&Tag signals in bigwig format.

### Bioinformatics

#### Principal Component Analysis (PCA)

PCA as carried out in R programming environment (R v4.3.0) in RStudio (v2023.03.0 Build 386) using the stats package (v3.6.2). Data were plotted using the ggplot package (v3.4.4).

#### Cytoscape

Protein-Protein interaction networks were created using Cytoscape (v3.10.0)^119^ and stringApp (v2.1.0)^47^. A list on Uniprot accession numbers meeting the enrichment standards were input into stringApp. Clusters were generated using Markov clustering within stringApp using a granularity parameter of 4.

#### Gene Set Enrichment (GSE) analysis

GSE analysis^120^ was performed on the normalized counts output by DEseq2 using the GSEA (4.3.2) software package. The h.all.v2023.Hs.symbols.gmt and c2.all.v2023.1.hs.symbols.gmt gene sets from the Molecular Signatures Database were used for the analysis. Gene symbols were collapsed, permutations were performed on gene_set rather than phenotype and the chip platform selected was the Human_Ensembl_Gene_ID_MSigDB. v2023.1.Hs.chip. The Signal2Noise metric was used to rank genes for data generated from cell culture samples. The Diff_of_classes metric was used to rank genes for data generated from tumoroid models as only two replicates were performed.

#### STAR Fusion analysis

Raw sequencing data from control and *PAX3::WWTR1* knockdown models were trimmed to remove adapters and keep high quality reads by using trim galore v0.6.10. Thereafter, STAR (v2.7.8a) was used to align read counts against the human genome (Gencode v. 37) and STARFusion (v1.10.0) was used to identify candidate fusion transcripts, also against the aforementioned version of the human genome. The function featureCounts from the subread package (v2.0.6) was used to extract raw read counts from the aligned files.

#### Differential expression and pathway analysis

Raw read counts were loaded into *R* (v4.2.1). Differential analysis was performed using DESeq2 (v1.36.0) by using condition (i.e., control or fusion knockdown) as contrast. Limma (v3.52.2) was used to correct for potential batch effects from the technical replicates. Pathway analyses was performed on differentially expressed genes using the package clusterProfiler (v4.4.4).

#### Gene Ontology enrichment analysis

Gene ontology enrichment analysis was performed using the g:Profiler2^121^ web portal (version *e113_eg59_p19_f6a03c19*, database updated on 23/05/2025). To reduce redundancy, terms were filtered by algorithmically highlighted annotations that include between 5 and 500 genes.

#### Dose response curve fitting

Dose response curves were fit to normalized CellTiter-Glo luminescence readings with the dose log_10_ transformed in GraphPad Prism (v10.1.2) using the formula: Y=Bottom + (Top-Bottom)/(1 + 10^((X-LogIC50))). Top value was constrained to 100.

#### Zip synergy score calculation

Zip synergy scores were calculated using the web based SynergyFinder+ tool (07.09.2024-R-3.10.3)^78^.

#### Statistical tests

Curve fitting and statistical tests for cell growth analysis, tumor growth, proximity ligation, immunoblot densitometry, IC50 calculations, and survival were performed using GraphPad Prism (v10.1.2). All other statistical tests were performed using R (v4.5.0).

#### MAGeCK CRISPR screen analysis

Screen analysis was performed using MAGECK-RRA and MLE^122^ for the kinome and interactome screen respectively to identify genes selectively enriched or depleted compared to the control.

#### Normalized Enrichment Score (NES) permutation test

Spearman correlation coefficients for GSE NES scores were calculated across each data set. NES scores were then randomly permutated 2000 times and each time Spearman coefficients recalculated. The measured coefficient distribution was then compared to the distribution of permutated coefficients and *p*-values calculated.

#### DepMap analysis

DepMap^123^, Broad (2025). DepMap Public 222Q2. CRISPR_Gene_Effect.csv and Sample_info.csv was utilized for this analysis. FP-RMS samples were first identified from the sample information. Interactome genes were then subset and the mean gene effects across samples were computed for FP-RMS samples and the remainder. Mean FP_RMS gene effects were subtracted from the remainder to calculate gene effect differences and adjusted p-values were determined using individual sample gene dependency scores.

### Reporting summary

Further information on research design is available in the Nature Portfolio Reporting Summary linked to this article.

## Supplementary information

Supplementary Information

Description of Additional Supplementary Files

Supplementary Dataset 1

Supplementary Dataset 2

Supplementary Dataset 3

Supplementary Dataset 4

Supplementary Dataset 5

Supplementary Dataset 6

Supplementary Dataset 7

Supplementary Dataset 8

Supplementary Dataset 9

Supplementary Dataset 10

Supplementary Dataset 11

Supplementary Dataset 12

Supplementary Dataset 13

Supplementary Dataset 14

Supplementary Dataset 15

Supplementary Dataset 16

Reporting Summary

Transparent Peer Review file

## Source data

Source Data

## Data Availability

The mass spectrometry proteomics data have been deposited to the ProteomeXchange Consortium via the PRIDE^124^ partner repository with the dataset identifiers PXD047629, PXD047630, PXD051746, and PXD071912. RNA sequencing data pertaining to the RH4-flag cells rescued with oncofusion-TurboV5 have been deposited to the GEO repository with the series record accession number GSE250125. RNA sequencing data pertaining to the RH4-flag cells targeted with individual sgRNA have been deposited to the GEO repository with the series record accession number GSE314949. CRISPR Sequencing Data pertaining to interactome screen have been deposited to the GEO repository with the series record accession number GSE314907. CRISPR Sequencing Data pertaining to Kinome screen have been deposited to the GEO repository with the series record accession number GSE314862. CUT&Tag DNA sequencing data have been deposited to the GEO repository with the series record accession number GSE285855. RNA sequencing data pertaining to the RMS000EEC tumoroids have been deposited to ArrayExpress with the accession number E-MTAB-13715. Normalized peptide count data was obtained from Ref. ^32^ for comparison of protein enrichment. ChimerSeq^92^ and FusionGDB2^93–95^ datasets were accessed through the relevant web portal on 12-4-2023. FOdb-II dataset was obtained from ref. ^96^. DepMap Public 222Q2. CRISPR_Gene_Effect.csv and Sample_info.csv was accessed through the relevant web portal. Source data are provided with this paper.
