# Supplementary material for: Fusion-positive rhabdomyosarcoma oncofusions share a common interactome
Source: Nat Commun. 2026 May 28;17:6933. doi: 10.1038/s41467-026-73749-y (PMC13389464; doi:10.1038/s41467-026-73749-y)
Supplement: Supplementary file 1 — Supplementary Information [file 41467_2026_73749_MOESM1_ESM.pdf]

Supplementary Information

Zimmerman_SuppFig1

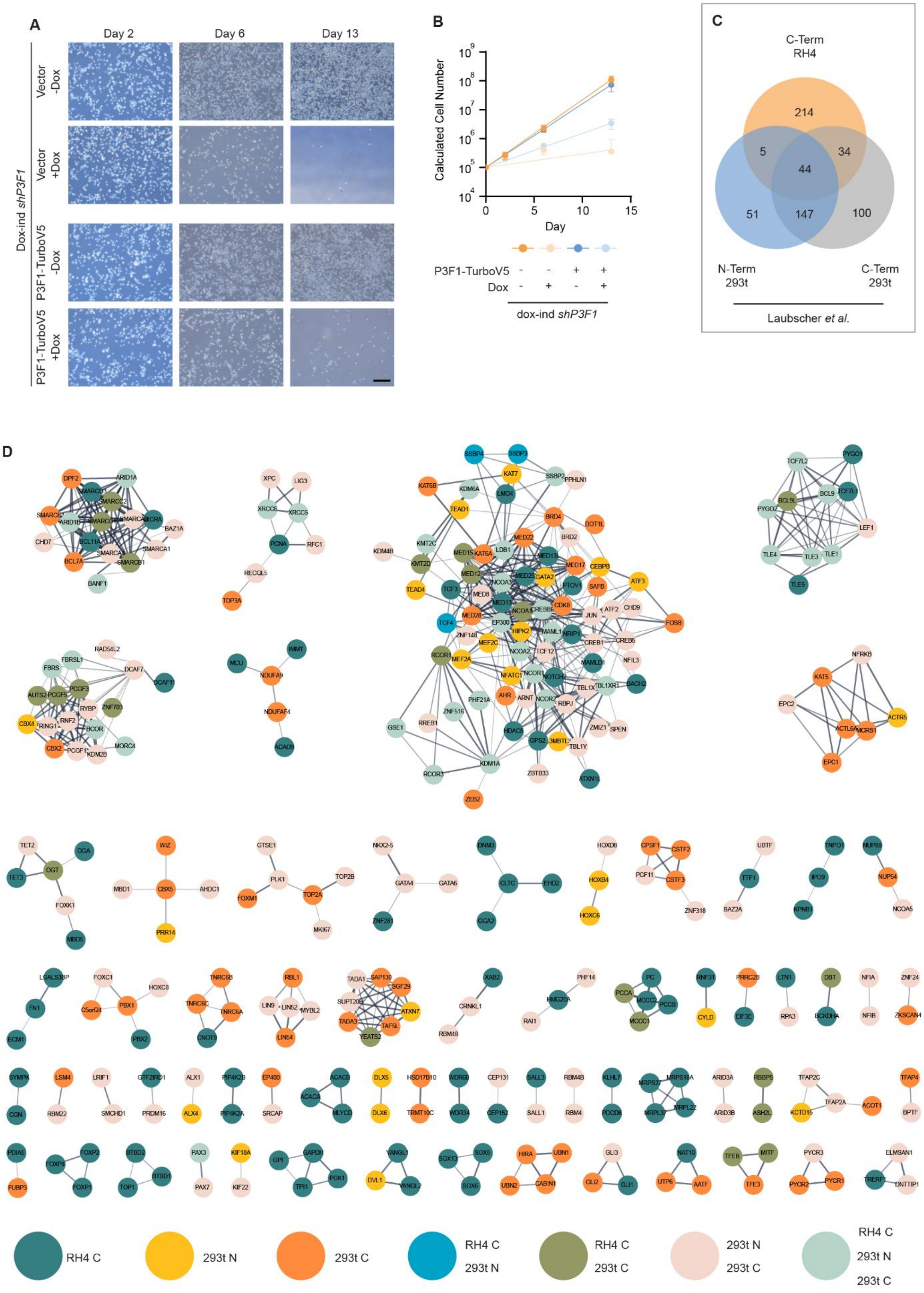

Supplemental Figure 1. ***A***) Representative image of RH4-flag cells analyzed in **Fig. 1B** and **Supp. Fig. 1B**. Scale Bar = 250 μm. ***B***) Scatter plot representing cell growth assays after doxycycline induction of *shP3F1*. Cells were counted on days 2, 6, and 13. Cells were split on day 4, 7, and 11 at a 1:5 ratio and factored into the calculated cell number. (n = 6 technical replicates from 2 independent experiments. ***C***) Venn diagram of proteins identified by proximity labeling by P3F1-TurboV5 (fold-change > 5 and $p < 0.01$) performed in human RH4-flag cancer cells compared to previous[32] proximity labeling with P3F1 fused to the BirA* tag on both the N- and C-terminus and expressed in 293T virally-transformed kidney cells (fold change > 2 and p < 0.01). ***D***) Markov clustered interaction network of all proteins identified in **A** based on STRING score. Color denotes the dataset.

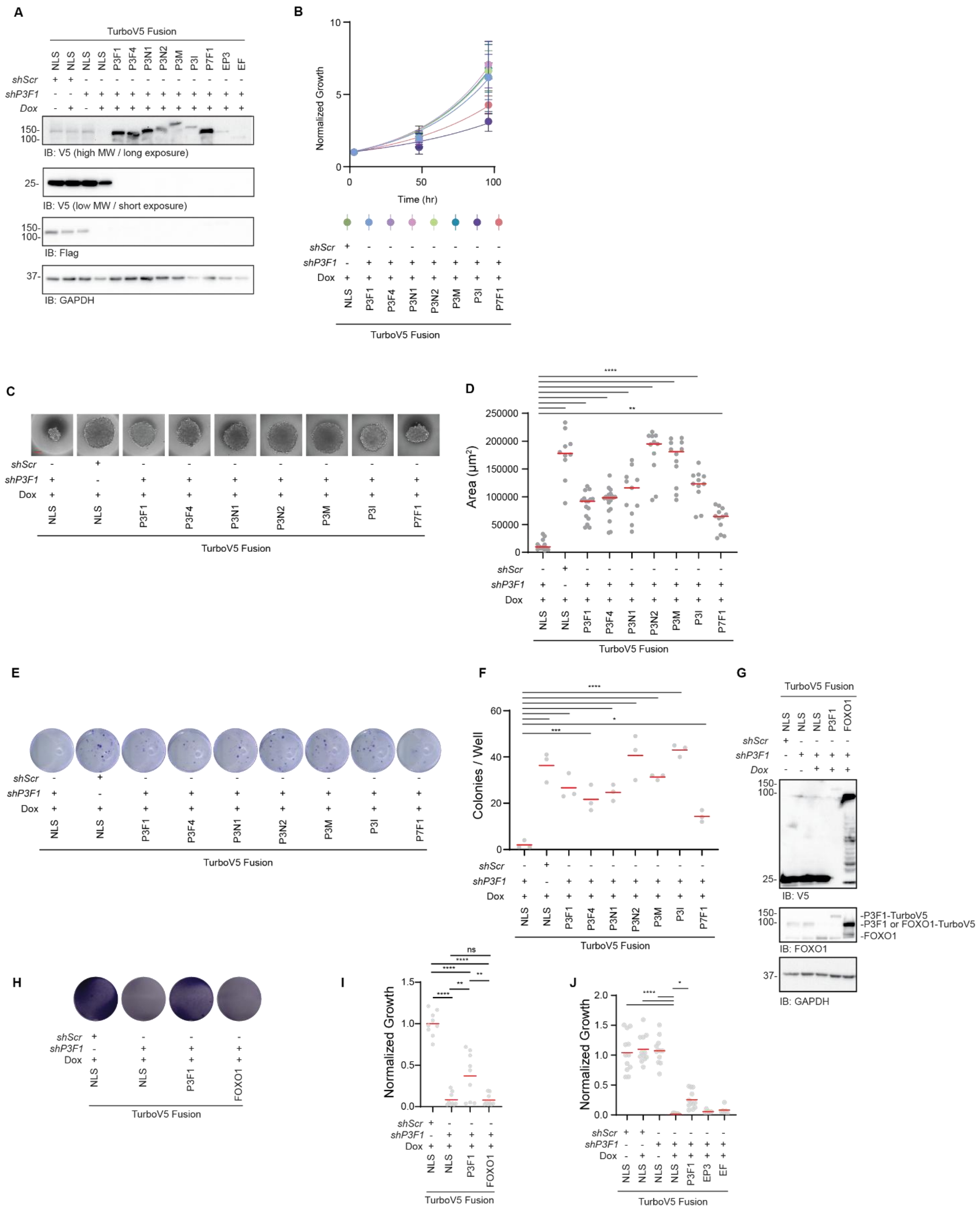
A
TurboV5 Fusion
NLS NLS NLS NLS P3F1 P3F4 P3N1 P3N2 P3M P3I P7F1 EP3 EF
shScr
shP3F1
Dox
IB: V5 (high MW / long exposure)
IB: V5 (low MW / short exposure)
IB: Flag
IB: GAPDH
B
Normalized Growth
Time (hr)
NLS P3F1 P3F4 P3N1 P3N2 P3M P3I P7F1
TurboV5 Fusion
C
TurboV5 Fusion
D
Area (µm²)
TurboV5 Fusion
E
TurboV5 Fusion
F
Colonies / Well
TurboV5 Fusion
G
TurboV5 Fusion
NLS NLS NLS P3F1 FOXO1
IB: V5
-P3F1-TurboV5
-P3F1 or FOXO1-TurboV5
-FOXO1
IB: FOXO1
IB: GAPDH
H
TurboV5 Fusion
I
Normalized Growth
TurboV5 Fusion
J
Normalized Growth
NLS NLS NLS NLS P3F1 EP3 EF
TurboV5 Fusion

Supplemental Figure 2. ***A***) Immunoblot analysis (IB) with the indicated antibodies of RH4-flag cells transduced with the indicated TurboV5 fusion and treated with doxycycline (Dox) for 14 days to induce expression of the indicated *shRNA*s prior to analysis. ***B***) Cell growth curves beginning 14 days after doxycycline induction. Normalized data were fit to an exponential to determine doubling time presented in **Fig. 2F**. (*n* = 9 technical replicates from 3 independent experiments). ***C***) Representative images of 9-day spheroid cultures of RH4-flag cells expressing the indicated *shRNA* and TurboV5 fusion. Scalebar = 100 µm. ***D***) Quantification of spheroid area from cells in B. (*n*> 20 spheroids from two independent experiments. Red bar = mean. One-way ANOVA.) ***E***) Representative images of crystal-violet staining of colonies of RH4-flag cells expressing the indicate proteins and *shRNA*s. ***F***) Quantification of colony formation of cells from **E**. (*n* = 3 wells per condition. Red bar = mean. One-way ANOVA.) ***G***) Immunoblot analysis (IB) with the indicated antibodies of RH4-flag cells transduced with the indicated TurboV5 fusion and treated with doxycycline (Dox) for 14 days to induce expression of the indicated *shRNA*s prior to analysis. ***H***) Representative images of crystal-violet staining of colonies of RH4-flag cells expressing the indicate proteins and *shRNA*s. ***I***) Quantification of crystal violet staining of cells from **H** and other replicates. (*n* = 9 wells from 3 independent experiments. Red bar = mean. One-way ANOVA.) ***J***) Quantification of normalized growth rate of RH4-flag cells expressing the indicated *shRNA* and TurboV5 fusion constructs, induced with doxycycline on day 0, counted at day 13, and then growth normalized to RH4-flag cells transduced with *shScr* and TurboV5-NLS and induced with doxycycline. (*n* =9 from 3 independent experiments. Unpaired t-test).

*$p < 0.05$, **$p < 0.01$, ***$p < 0.001$ and ****$p < 0.0001$.

A

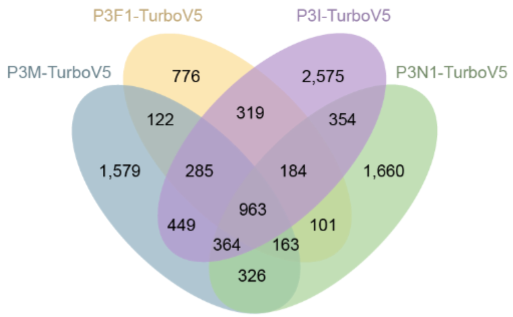

B

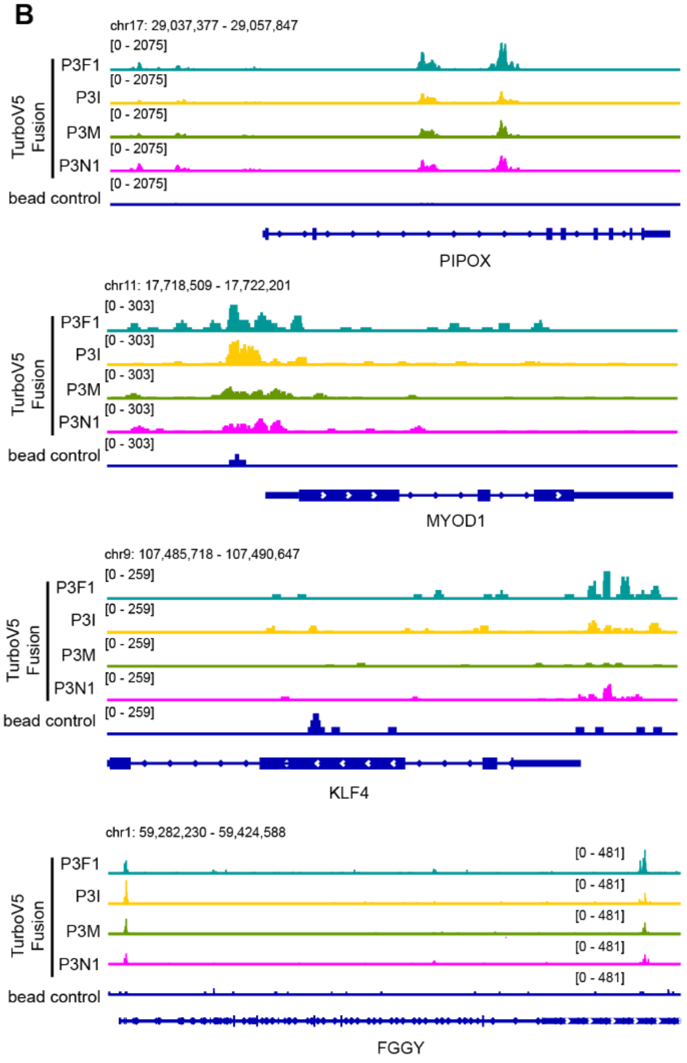

C

Shared Peaks (963)

Motif	Name	% of Targets	p-value
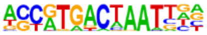	PAX3::FOXO1	8.20%	1e-41
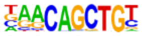	MYF5	18.48%	1e-30
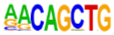	MYOG	23.88%	1e-24
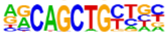	MYOD	18.69%	1e-20

Supplemental Figure 3. ***A)*** Venn diagram of the overlap of the called peaks for V5-tagged P3F1, P3I, P3M, and P3N1 ectopically expressed in *shP3F1* RF4-flag cells. ***B***) Integrative Genome Viewer (IGV) views of the CUT&Tag signals of P3F1, P3I, P3M, and P3N1 at the known P3F1 targets in *shP3F1* RF4-flag cells. ***C)*** The most enriched motifs from a motif search analysis at the called peaks in common between P3F1, P3I, P3M, and P3N1.

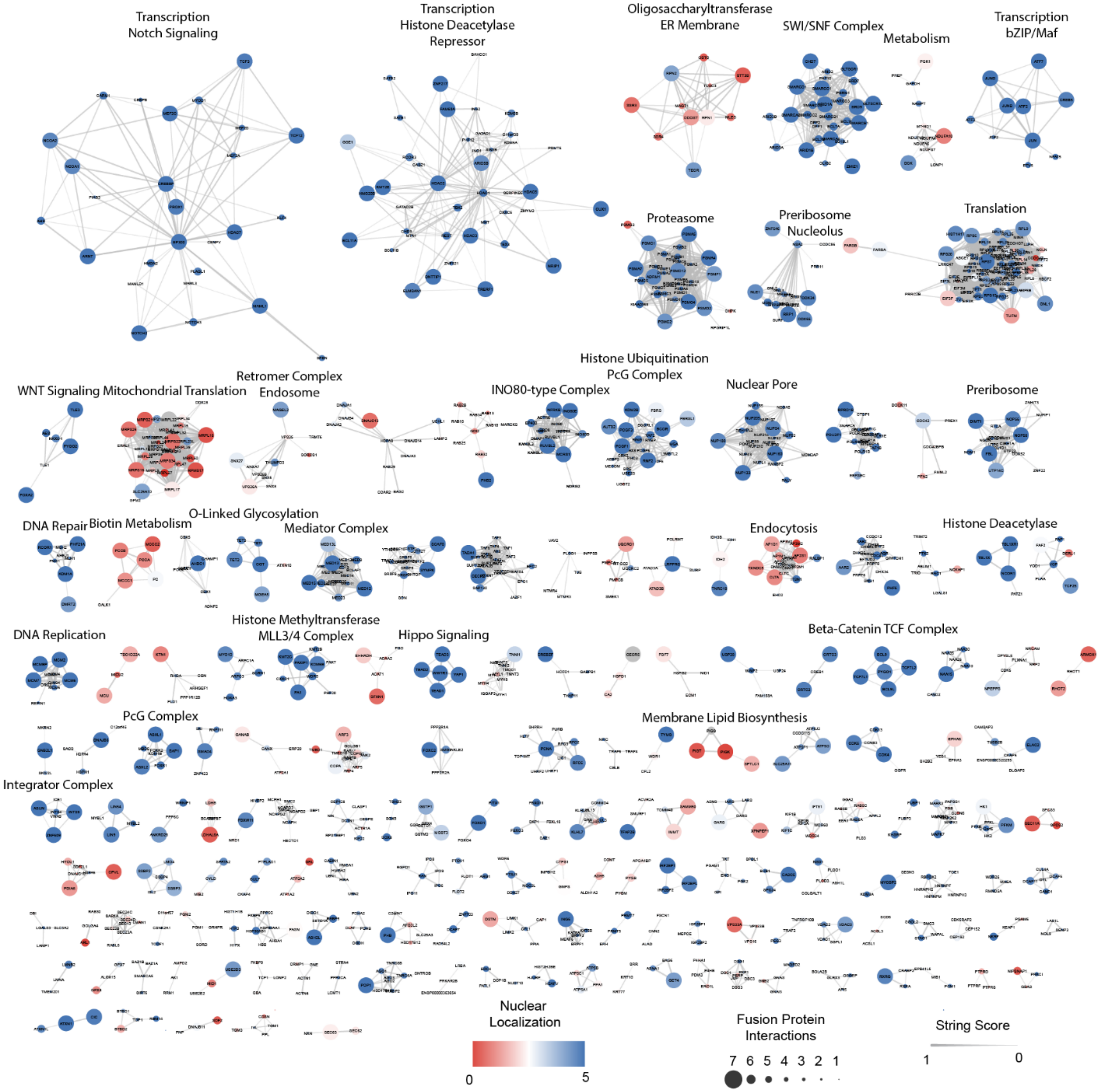

Supplemental Figure 4. Markov clustered STRING interaction network of all proteins identified which were enriched in at least one fusion protein TurboID data set (fold-change > 1.5, $p < 0.05$). Clusters with 3 proteins found to interact with all 7 fusion proteins are annotated using STRING enrichment analysis. Diagram does not include proteins that do not have known interactions with other members.

Zimmerman_SuppFig5

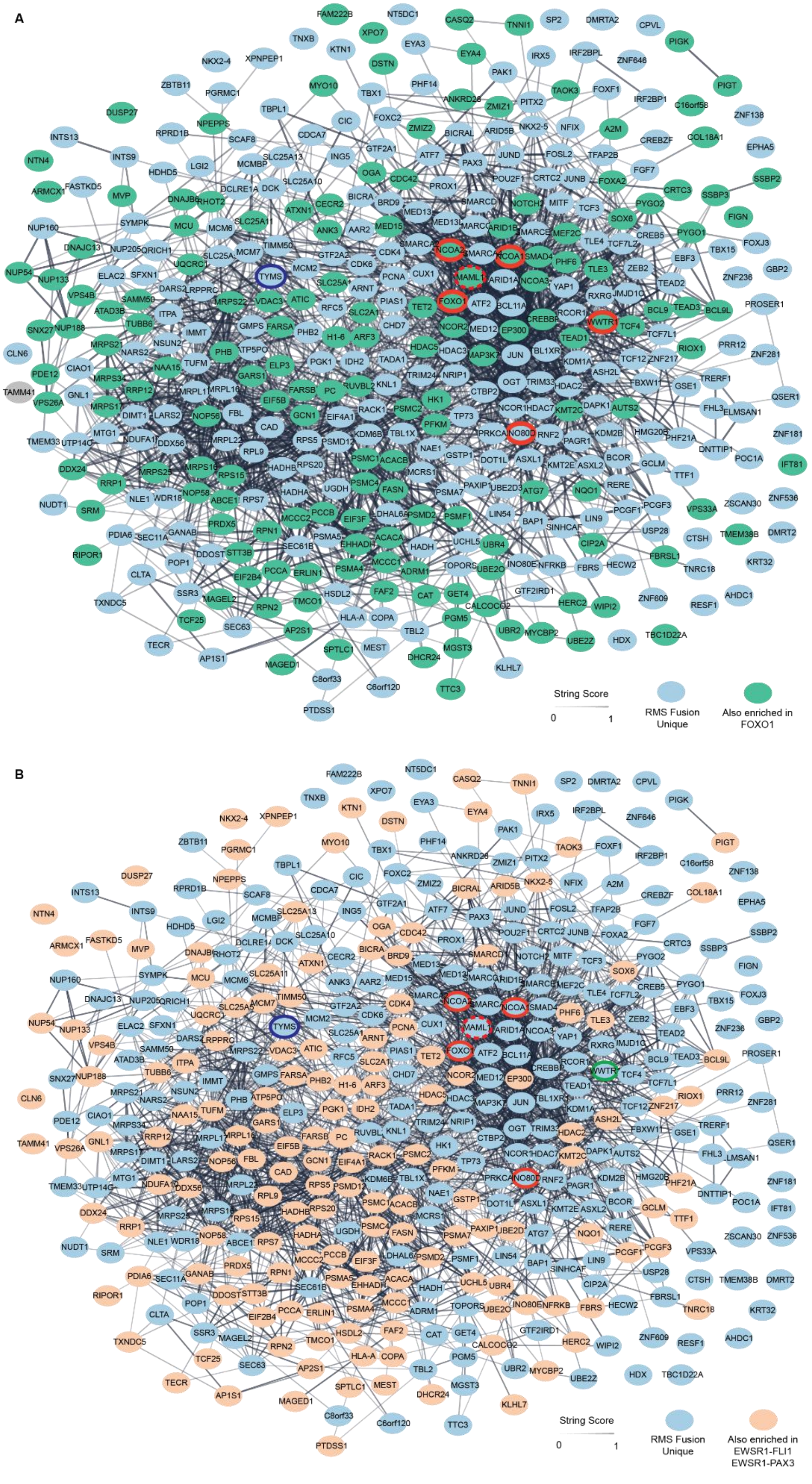

Supplemental Figure 5. ***A, B***) STRING protein interaction network diagrams of the proteins in the common interactome color-coded to indicate whether or not the protein was also found in ***A***) FOXO1-TurboV5 or ***B***) EF-TurboV5 or EP3-TurboV5 data sets. Red outline: full-length version of C-terminal fusion partners. Dashed red outline: MAML1 (paralog of MAML3). Green outline: WWTR1. Dark blue outline: TYMS.

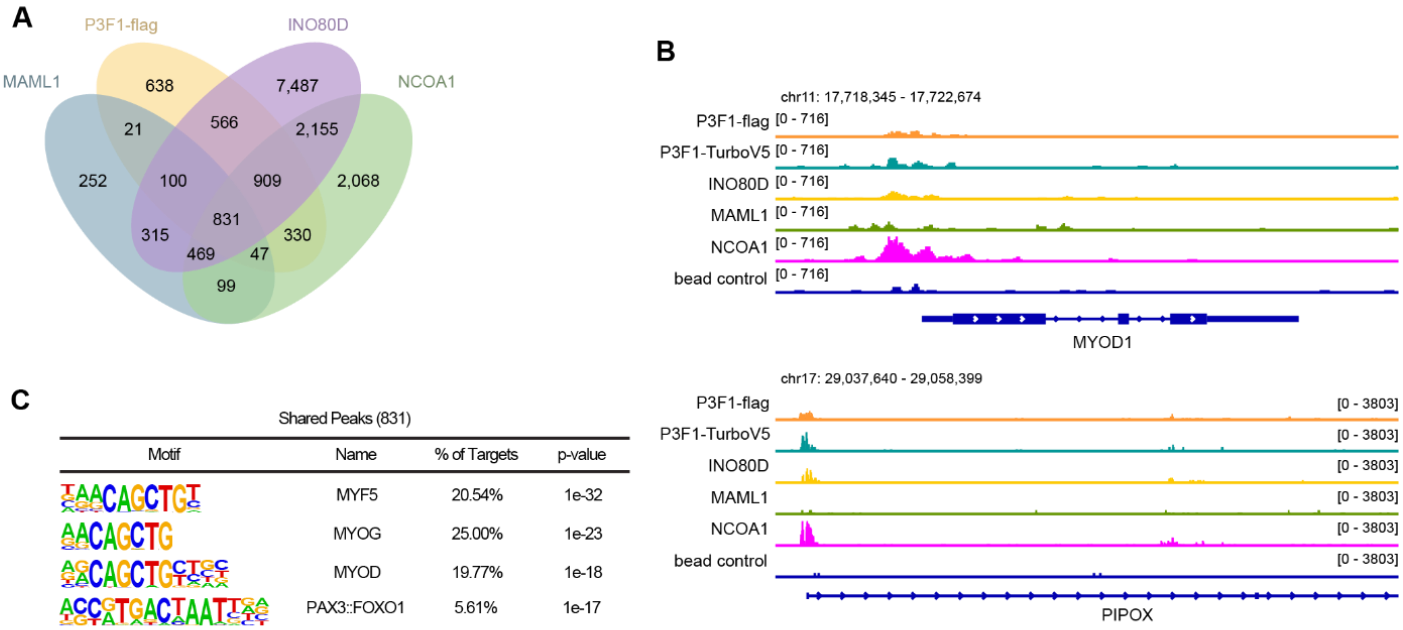

Supplemental Figure 6. ***A***) Venn diagram of the overlap of the called peaks for P3F1-flag, INO80D, MAML1 and NCOA1 in RF4-flag cells. ***B)*** Integrative Genome Viewer (IGV) tracks of the indicated CUT&Tag signals of endogenous P3F1-flag, ectopic P3F1-TurboV5, INO80D, MAML1, and NCOA1 at the known P3F1 targets in RH4-flag cells. ***C***) The most enriched motifs from a motif search analysis at the peaks commonly bound by P3F1-flag, INO80D, MAML1, and NCOA1 in RH4-flag cells.

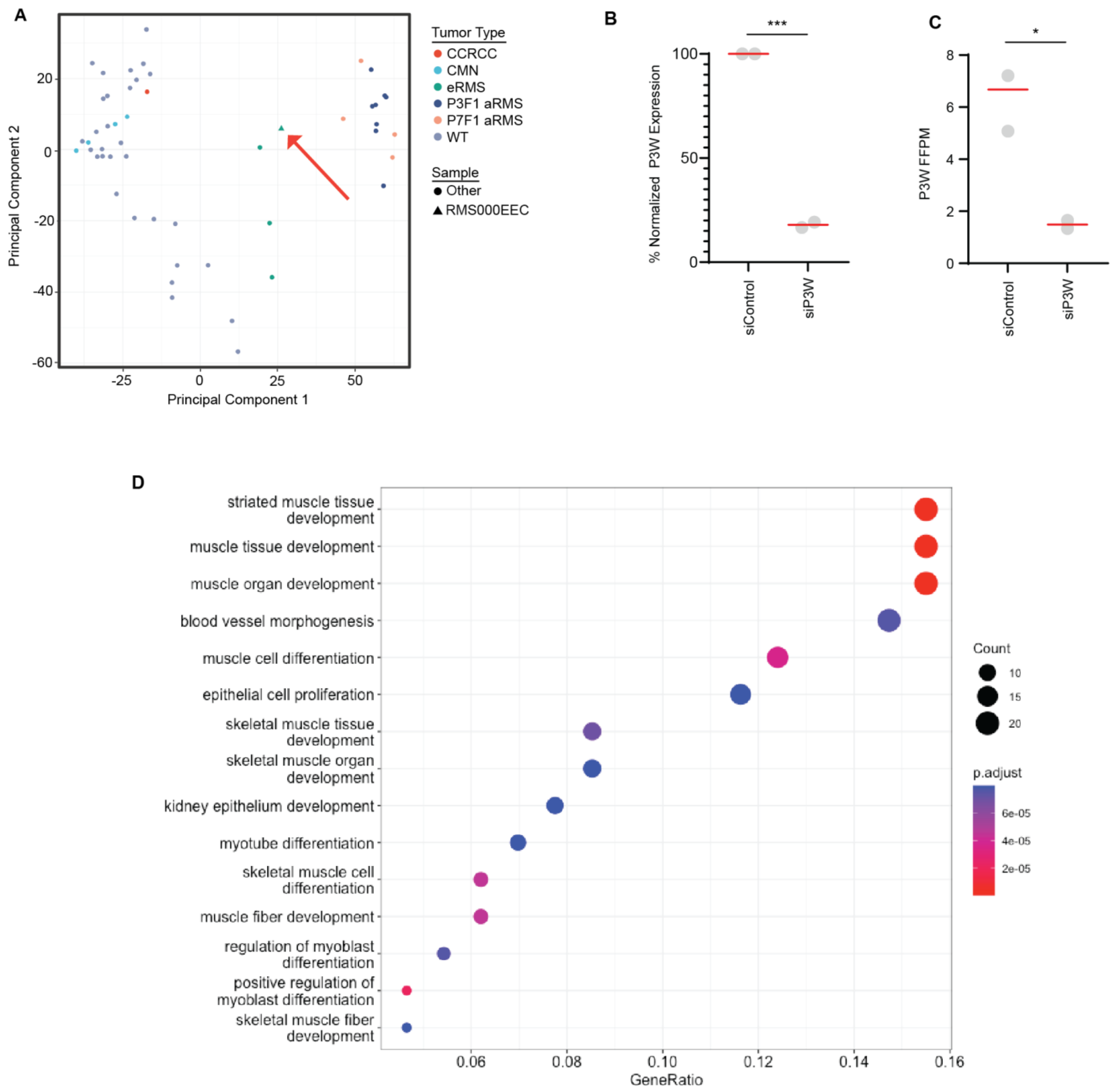

Supplemental Figure 7. ***A***) PCA of previously generated RNA-seq datasets[52] from primary tumor samples of various pediatric kidney and RMS tumors using all protein-coding genes. ***B)*** *P3W* transcript abundance determined by RT-qPCR analysis, normalized to *G6PD* housekeeping transcript and expressed as a percent, from control (siControl) versus *P3W* (siP3W) siRNA-treated RMS000EEC tumoroids. (n = 2 technical replicates per condition. T-test ***$p < 0.001$.) ***C)*** *P3W* fusion fragments per million total reads (FFPM) determine by STAR Fusion analysis from RNAseq datasets from control versus *P3W* siRNA-treated

RMS000EEC tumoroids. (*n* = 2 technical replicates per condition. t-test $*p < 0.05$). ***D)*** Pathway analysis of differentially expressed genes between control versus *P3W* siRNA-treated RMS000EEC tumoroids.

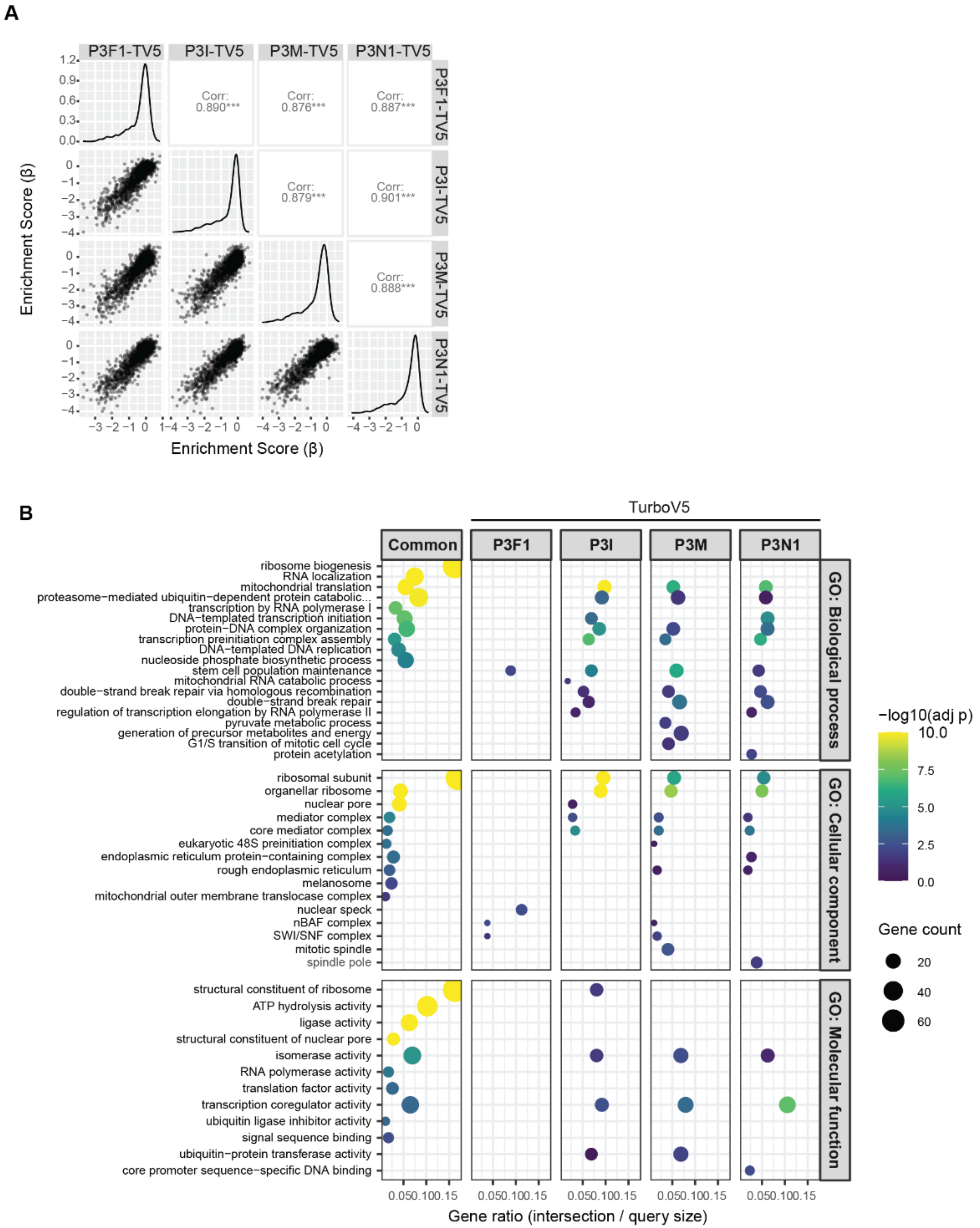

Supplemental Figure 8. ***A***) Faceted scatter, distribution plots and spearman correlation coefficients comparing the enrichment scores of target genes from the CRISPR/Cas9 loss-of-function screen in RH4-

flag *shP3F1* cells rescued with the indicated oncofusions. (*n* = 3 technical replicates per sample tested). ***B***) Bubble plot of GO: Biological Processes, Cellular Component, and Molecular Function term enrichment for sgRNA-target genes negatively enriched from **Fig. 6D** uniquely in each of the RH4 cells rescued with the indicated oncofusions, or in all four (common). Enrichment was assessed by gene ratio or the number of genes in each query classified by the ontology term (intersection) compared to the total number of genes queried. Circle size: gene count in intersection. Color: $-\text{Log}_{10}$(adjusted p value).

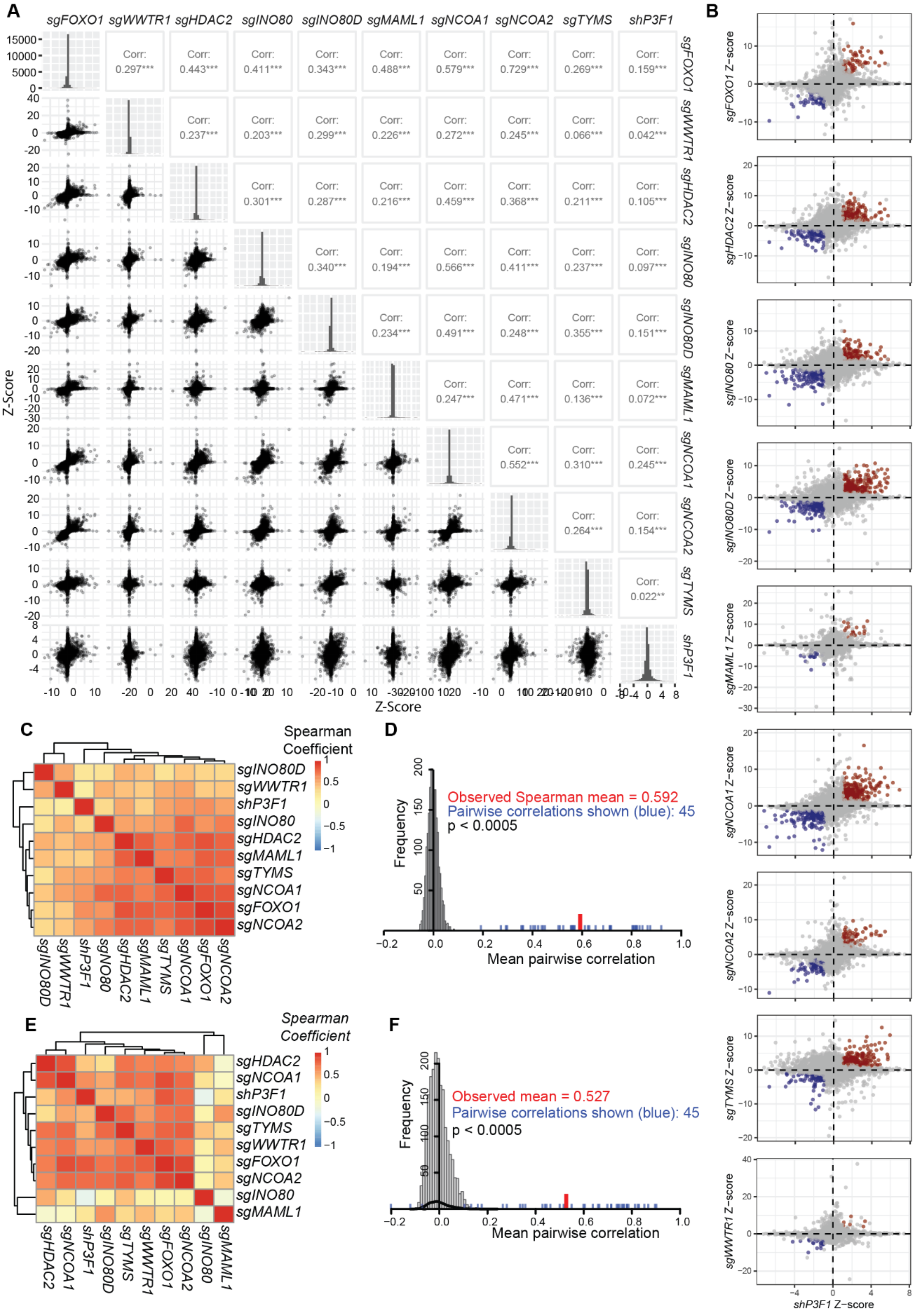
A
sgFOXO1 sgWWTR1 sgHDAC2 sgINO80 sgINO80D sgMAML1 sgNCOA1 sgNCOA2 sgTYMS shP3F1
Corr: 0.297*** Corr: 0.443*** Corr: 0.411*** Corr: 0.343*** Corr: 0.488*** Corr: 0.579*** Corr: 0.729*** Corr: 0.269*** Corr: 0.159***
Corr: 0.237*** Corr: 0.203*** Corr: 0.299*** Corr: 0.226*** Corr: 0.272*** Corr: 0.245*** Corr: 0.066*** Corr: 0.042***
Corr: 0.301*** Corr: 0.287*** Corr: 0.216*** Corr: 0.459*** Corr: 0.368*** Corr: 0.211*** Corr: 0.105***
Corr: 0.340*** Corr: 0.194*** Corr: 0.566*** Corr: 0.411*** Corr: 0.237*** Corr: 0.097***
Corr: 0.234*** Corr: 0.491*** Corr: 0.248*** Corr: 0.355*** Corr: 0.151***
Corr: 0.247*** Corr: 0.471*** Corr: 0.136*** Corr: 0.072***
Corr: 0.552*** Corr: 0.310*** Corr: 0.245***
Corr: 0.264*** Corr: 0.154***
Corr: 0.022**
Z-Score
Z-Score
B
sgFOXO1 Z-score
sgHDAC2 Z-score
sgINO80 Z-score
sgINO80D Z-score
sgMAML1 Z-score
sgNCOA1 Z-score
sgNCOA2 Z-score
sgTYMS Z-score
sgWWTR1 Z-score
shP3F1 Z-score
C
Spearman Coefficient
sgINO80D sgWWTR1 shP3F1 sgINO80 sgHDAC2 sgMAML1 sgTYMS sgNCOA1 sgFOXO1 sgNCOA2
D
Observed Spearman mean = 0.592
Pairwise correlations shown (blue): 45
p < 0.0005
Frequency
Mean pairwise correlation
E
Spearman Coefficient
sgHDAC2 sgNCOA1 shP3F1 sgINO80D sgTYMS sgWWTR1 sgFOXO1 sgNCOA2 sgINO80 sgMAML1
F
Observed mean = 0.527
Pairwise correlations shown (blue): 45
p < 0.0005
Frequency
Mean pairwise correlation

Supplemental Figure 9. ***A***) Faceted scatter, distribution plots and spearman correlation coefficients of changes in mRNA expression z-scores derived from the transcriptome of RH4-flag cells transduced with the indicated shRNA or sgRNA versus *shScr* or *sgNTC*. (*n* = 3 technical replicates per condition) **B**) Scatter plots from **A** comparing changes in transcript expression in RH4-flag cells transduced with the *shP3F1* and *shScr* to the indicated sgRNA and *sgNTC*. Blue and red coloring: transcripts commonly down- or up-regulated in both samples (2 standard deviations from mean, adj-p < 0.05). (*n* = 3 technical replicates per condition). ***C,E***) Heat map of Spearman correlation coefficients of normalized enrichment scores from gene set enrichment analysis using the ***C***) MSigDB hallmark gene sets or ***E***) the MSigDB C2 gene sets related to FP-RMS. Both analyses compare RH4-flag cells transduced with the indicated shRNA or sgRNA to an *shScr* or *sgNTC*. ***D,F***) Histogram of permutation test for the spearman coefficients reported in **C** or **E**, respectively.

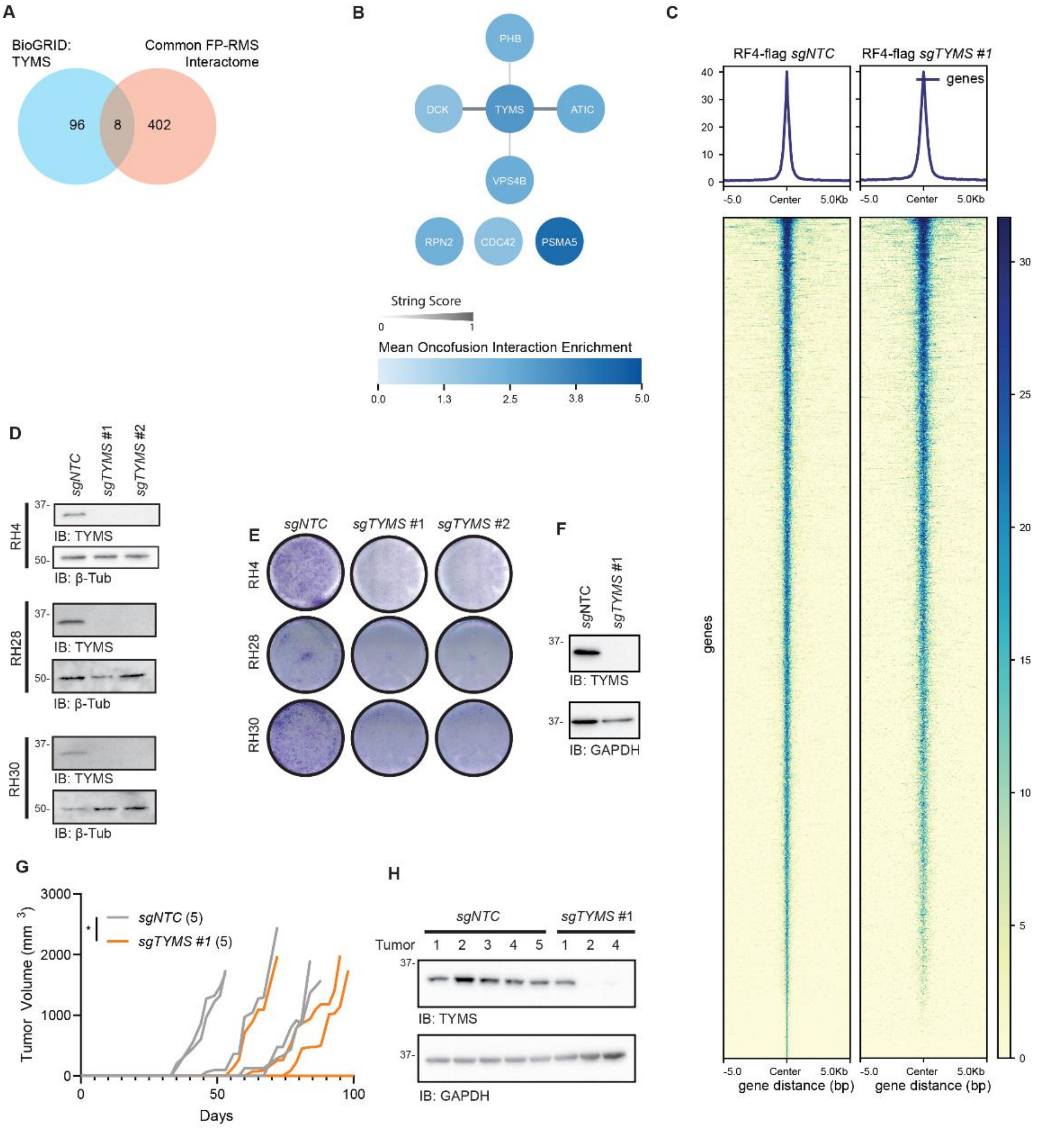

Supplemental Figure 10. ***A***) Venn diagram comparing known TYMS interactions from BioGRID to the common interactome. ***B***) STRING network diagram of the 8 overlapping proteins from **A**. ***C***) Heatmap of CUT&Tag signal for endogenous P3F1 (flag-tagged) in RH4-flag cells transduced with indicated sgRNA from ± 5 kb of the called endogenous P3F1 peaks from the *sgNTC* (Non-Targeting Control) sample. ***D***) Immunoblot analysis (IB) with the indicated antibodies of RH4, RH28, and RH30 cells transduced with the

indicated sgRNA. ***E***) Representative images of crystal-violet staining of colonies of RH4, RH28, and RH30 cells transduced with the indicated sgRNA. Images correspond to the analysis in **Fig. 8F.** ***F***) Immunoblot analysis (IB) with the indicated antibodies of RH28 cells transduced with the indicated sgRNA prior to xenograft transplant. ***G***) Tumor volume versus time of mice (n = 5) bearing subcutaneous xenograft tumors derived from RH28 cells transduced with the indicated sgRNA. (mixed effects model *$p < 0.05$). ***H***) Immunoblot analysis (IB) with the indicated antibodies of RH28 xenograft tumors from **G** transduced with the indicated sgRNA.

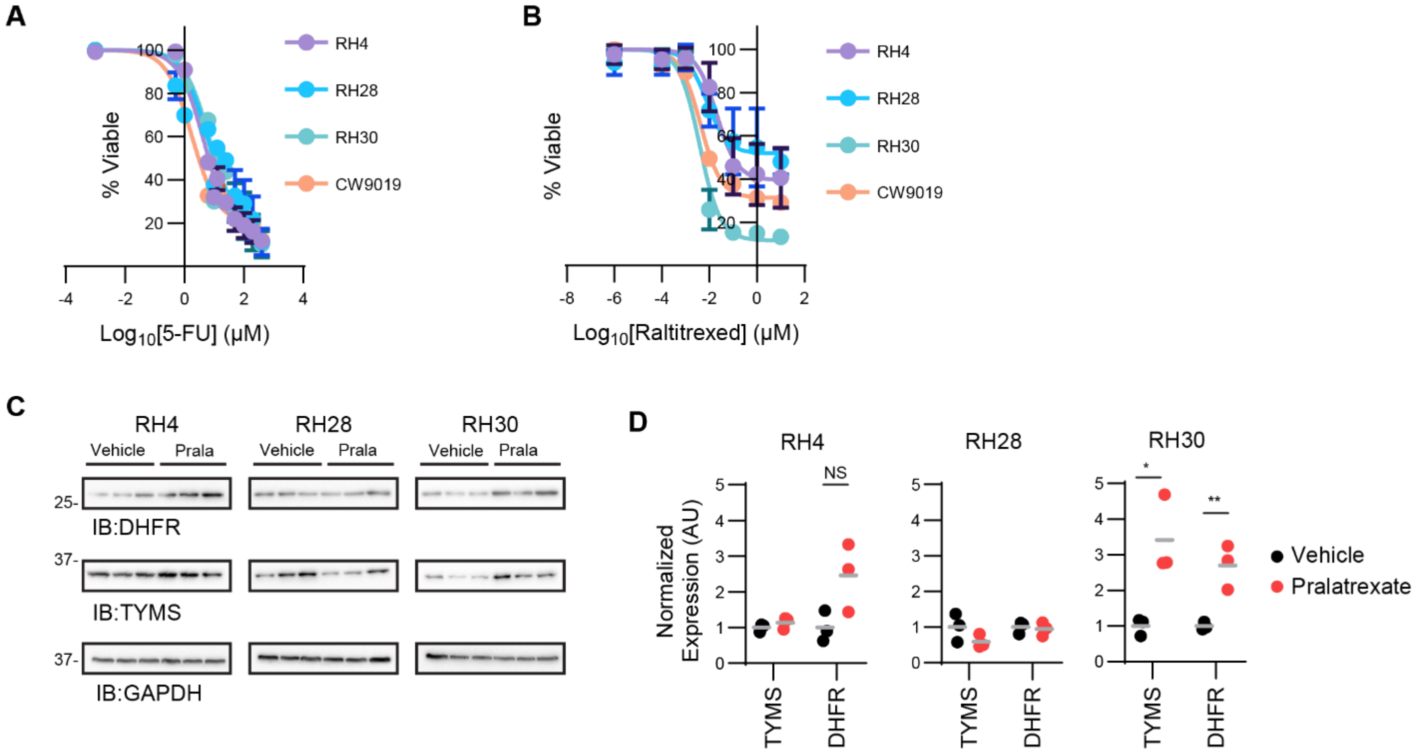

Supplemental Figure 11. ***A,B***) Scatter plot of mean dose response and fit curves represented as percent (%) viability versus concentration of ***A***) 5-fluorouricil (5-FU) of ***B***) Raltitrexed for RH4, RH28, RH30, and CW9019 cells. ***B***) Scatter plot of mean dose response and fit curves represented as percent (%) viability versus concentration of for RH4, RH28, RH30, and CW9019 cells, as determined by the CellTiter-Glo assay after 96 hours of treatment with the indicated inhibitor and normalized to the maximum mean value for each curve. Summaries of curve fitting, IC_{50} values, and *n* for each curve are provided in **Suppl. Table 13.** ***C***) Immunoblot analysis (IB) with the indicated antibodies of RH4, RH28, and RH30 xenograft tumors treated as indicated. **D**) Plot of the densitometric quantification of the immunoblots in ***C.*** Values are normalized to the vehicle control within each cell line (n = 3 tumor samples per cell line treatment, Unpaired t-test *$p < 0.05$, **$p < 0.01$).

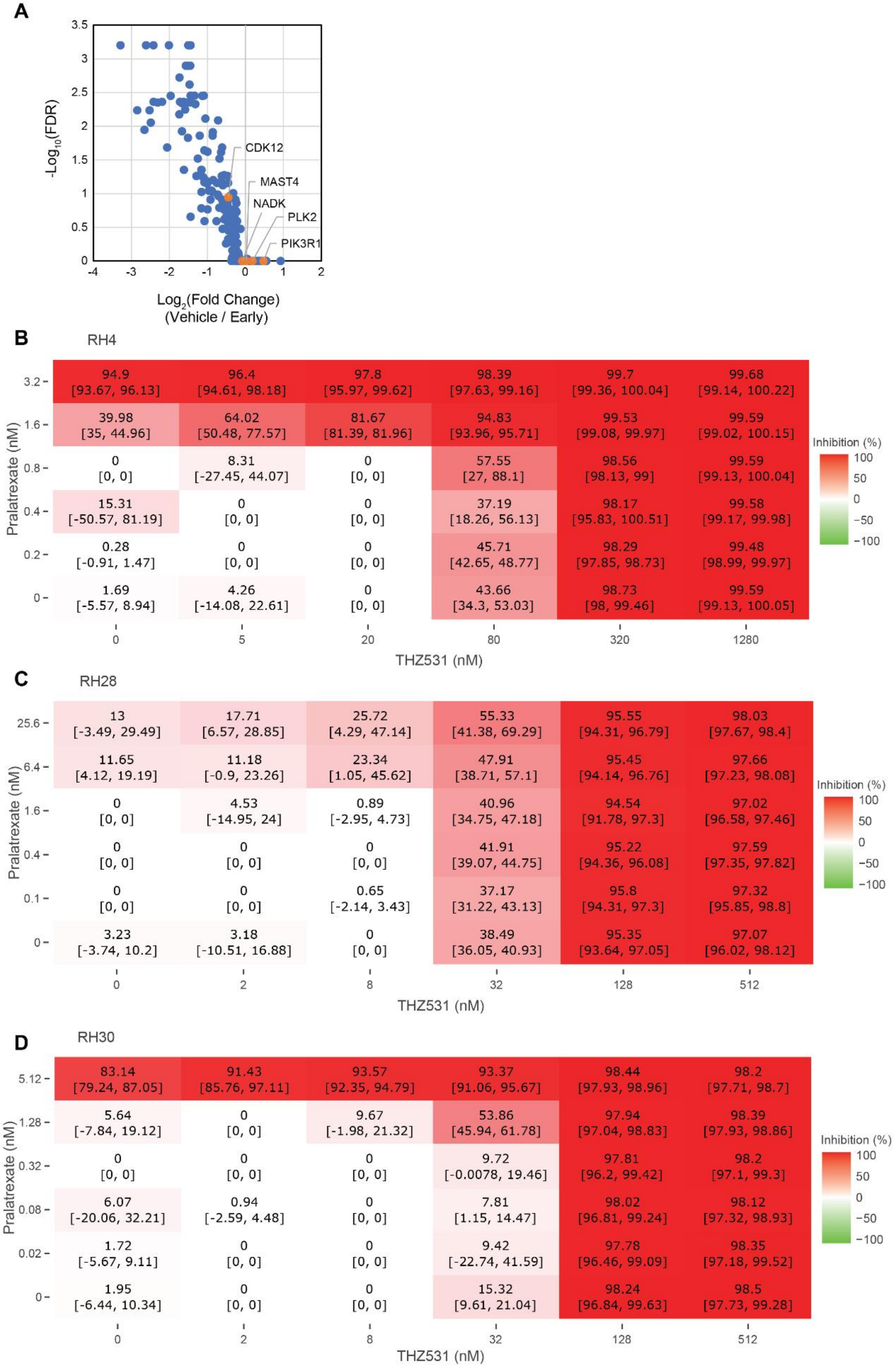

A
3.5
3
2.5
2
1.5
1
0.5
0
-4
-3
-2
-1
0
1
2
-Log10(FDR)
Log2(Fold Change)
(Vehicle / Early)
CDK12
MAST4
NADK
PLK2
PIK3R1
B
RH4
Pralatrexate (nM)
THZ531 (nM)
3.2: 94.9 [93.67, 96.13]; 96.4 [94.61, 98.18]; 97.8 [95.97, 99.62]; 98.39 [97.63, 99.16]; 99.7 [99.36, 100.04]; 99.68 [99.14, 100.22]
1.6: 39.98 [35, 44.96]; 64.02 [50.48, 77.57]; 81.67 [81.39, 81.96]; 94.83 [93.96, 95.71]; 99.53 [99.08, 99.97]; 99.59 [99.02, 100.15]
0.8: 0 [0, 0]; 8.31 [-27.45, 44.07]; 0 [0, 0]; 57.55 [27, 88.1]; 98.56 [98.13, 99]; 99.59 [99.13, 100.04]
0.4: 15.31 [-50.57, 81.19]; 0 [0, 0]; 0 [0, 0]; 37.19 [18.26, 56.13]; 98.17 [95.83, 100.51]; 99.58 [99.17, 99.98]
0.2: 0.28 [-0.91, 1.47]; 0 [0, 0]; 0 [0, 0]; 45.71 [42.65, 48.77]; 98.29 [97.85, 98.73]; 99.48 [98.99, 99.97]
0: 1.69 [-5.57, 8.94]; 4.26 [-14.08, 22.61]; 0 [0, 0]; 43.66 [34.3, 53.03]; 98.73 [98, 99.46]; 99.59 [99.13, 100.05]
0
5
20
80
320
1280
Inhibition (%)
100
50
0
−50
−100
C
RH28
Pralatrexate (nM)
THZ531 (nM)
25.6: 13 [-3.49, 29.49]; 17.71 [6.57, 28.85]; 25.72 [4.29, 47.14]; 55.33 [41.38, 69.29]; 95.55 [94.31, 96.79]; 98.03 [97.67, 98.4]
6.4: 11.65 [4.12, 19.19]; 11.18 [-0.9, 23.26]; 23.34 [1.05, 45.62]; 47.91 [38.71, 57.1]; 95.45 [94.14, 96.76]; 97.66 [97.23, 98.08]
1.6: 0 [0, 0]; 4.53 [-14.95, 24]; 0.89 [-2.95, 4.73]; 40.96 [34.75, 47.18]; 94.54 [91.78, 97.3]; 97.02 [96.58, 97.46]
0.4: 0 [0, 0]; 0 [0, 0]; 0 [0, 0]; 41.91 [39.07, 44.75]; 95.22 [94.36, 96.08]; 97.59 [97.35, 97.82]
0.1: 0 [0, 0]; 0 [0, 0]; 0.65 [-2.14, 3.43]; 37.17 [31.22, 43.13]; 95.8 [94.31, 97.3]; 97.32 [95.85, 98.8]
0: 3.23 [-3.74, 10.2]; 3.18 [-10.51, 16.88]; 0 [0, 0]; 38.49 [36.05, 40.93]; 95.35 [93.64, 97.05]; 97.07 [96.02, 98.12]
0
2
8
32
128
512
Inhibition (%)
100
50
0
−50
−100
D
RH30
Pralatrexate (nM)
THZ531 (nM)
5.12: 83.14 [79.24, 87.05]; 91.43 [85.76, 97.11]; 93.57 [92.35, 94.79]; 93.37 [91.06, 95.67]; 98.44 [97.93, 98.96]; 98.2 [97.71, 98.7]
1.28: 5.64 [-7.84, 19.12]; 0 [0, 0]; 9.67 [-1.98, 21.32]; 53.86 [45.94, 61.78]; 97.94 [97.04, 98.83]; 98.39 [97.93, 98.86]
0.32: 0 [0, 0]; 0 [0, 0]; 0 [0, 0]; 9.72 [-0.0078, 19.46]; 97.81 [96.2, 99.42]; 98.2 [97.1, 99.3]
0.08: 6.07 [-20.06, 32.21]; 0.94 [-2.59, 4.48]; 0 [0, 0]; 7.81 [1.15, 14.47]; 98.02 [96.81, 99.24]; 98.12 [97.32, 98.93]
0.02: 1.72 [-5.67, 9.11]; 0 [0, 0]; 0 [0, 0]; 9.42 [-22.74, 41.59]; 97.78 [96.46, 99.09]; 98.35 [97.18, 99.52]
0: 1.95 [-6.44, 10.34]; 0 [0, 0]; 0 [0, 0]; 15.32 [9.61, 21.04]; 98.24 [96.84, 99.63]; 98.5 [97.73, 99.28]
0
2
8
32
128
512
Inhibition (%)
100
50
0
−50
−100

Supplemental Figure 12. ***A***) Volcano plot of the $\log_2$ fold change versus the $-\log_{10}$ false discovery rate (FDR) of differential gene scores comparing RH4 cells transduced with a sgRNA library targeting the kinome and treated for 10 days with DMSO (vehicle) or harvested directly after puromycin selection (early). (*n* = 3 technical replicates per condition. Orange: data points highlighted in **Fig. 10B**). ***B-D***) Heatmap of dose response matrix for ***B***) RH4, ***C***) RH28, and ***D***) RH30 cells treated with pralatrexate and THZ531. Values represent mean [95% confidence intervals] of % inhibition (*n* = 3). Data used to calculate Zip synergy scores in **Fig. 10C-E**.

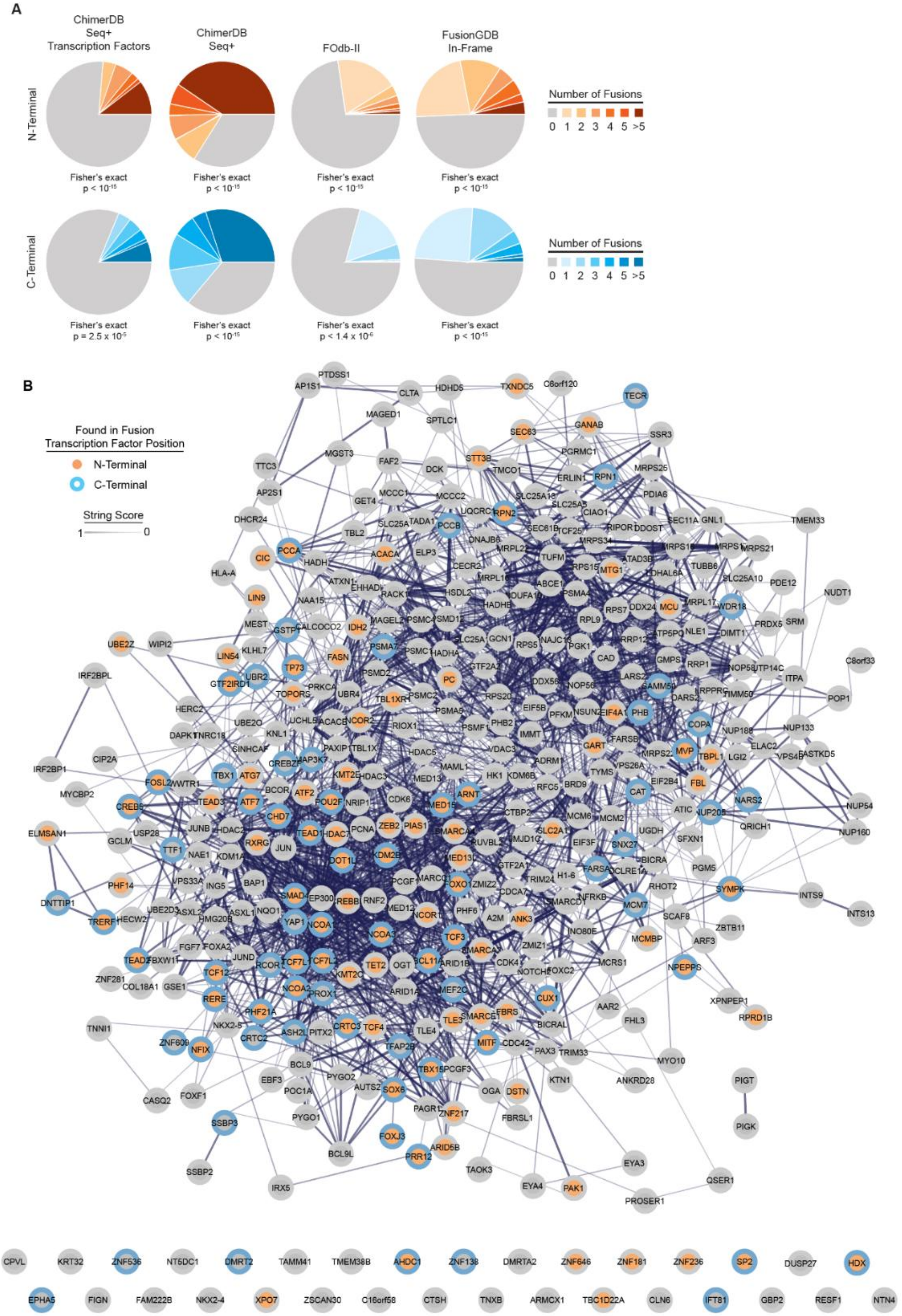
A
ChimerDB Seq+ Transcription Factors
ChimerDB Seq+
FOdb-II
FusionGDB In-Frame
N-Terminal
C-Terminal
Number of Fusions
0 1 2 3 4 5 >5
Fisher's exact p < 10-15
Fisher's exact p = 2.5 x 10-5
Fisher's exact p < 1.4 x 10-6
B
Found in Fusion Transcription Factor Position
N-Terminal
C-Terminal
String Score
1 0
CPVL
KRT32
ZNF536
NT5DC1
DMRT2
TAMM41
TMEM38B
AHDC1
ZNF138
DMRTA2
ZNF646
ZNF181
ZNF236
SP2
DUSP27
HDX
EPHA5
FIGN
FAM222B
NKX2-4
XPO7
ZSCAN30
C16orf58
CTSH
TNXB
ARMCX1
TBC1D22A
CLN6
IFT81
GBP2
RESF1
NTN4

Supplemental Figure 13. ***A***) Pie charts of the number of the 410 proteins of the common oncofusion interactome detected in the indicated number of fusion genes at either the N- or C-terminal position, collated from ChimerDB[92], FOdb-II[96], and FusionGDB[94]. ***B***) Diagram of the STRING protein interaction network of proteins in the common interactome, color coded to indicate the position in which the protein is found in other fusion transcription factors. *Bottom*: interactome proteins that are not part of the STRING interaction network.

Zimmerman_SuppFig2A immunoblot

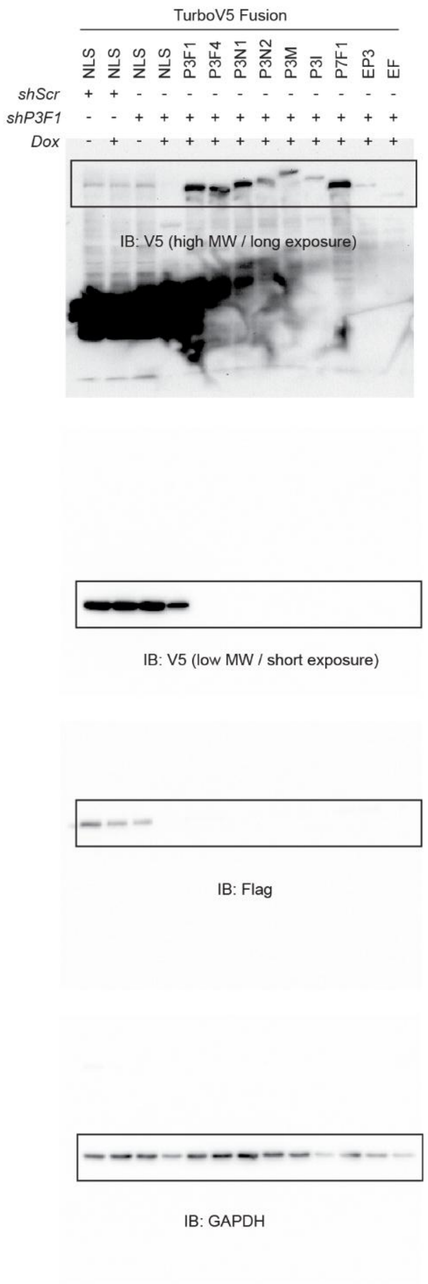

Zimmerman_SuppFig2G immunoblot

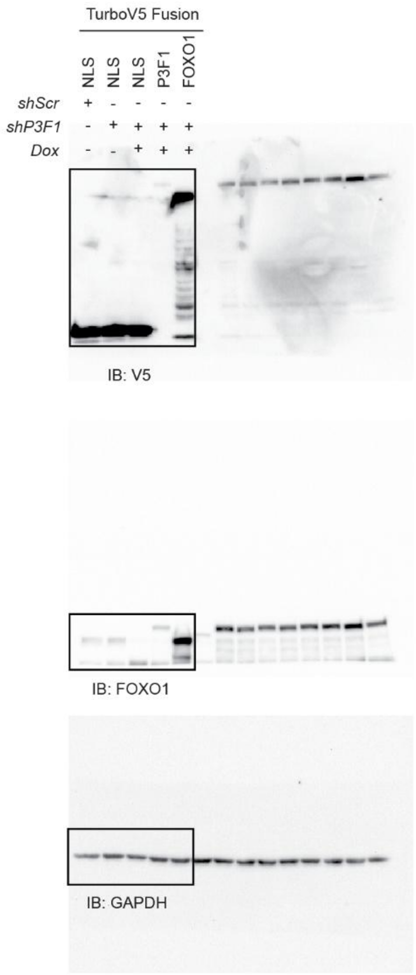

Zimmerman_SuppFig10D immunoblot

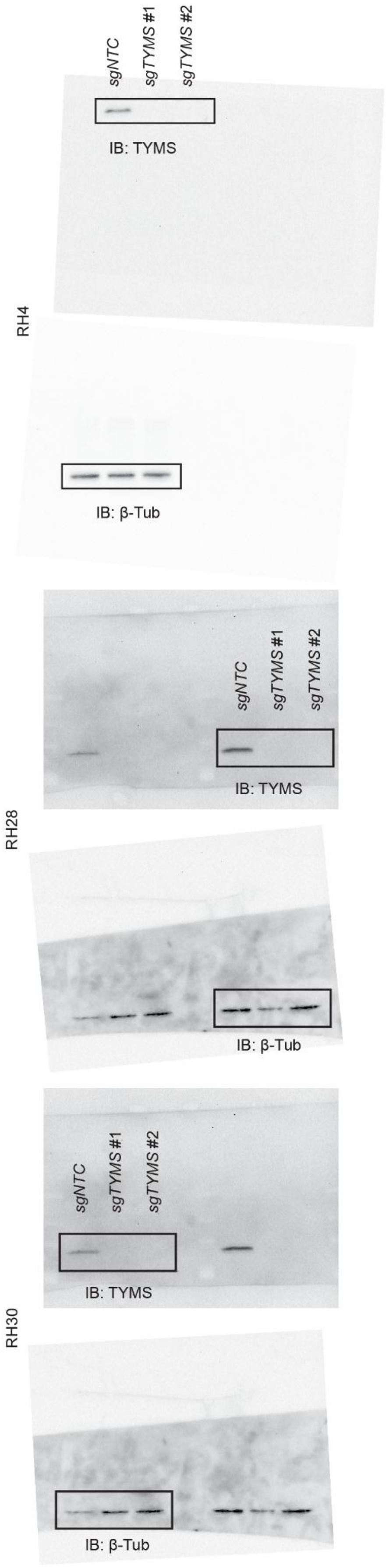

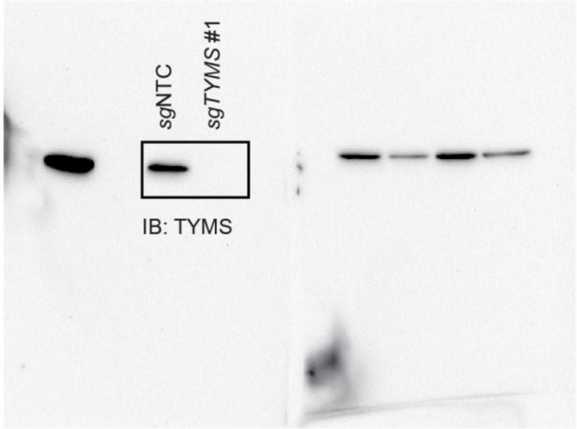
sgNTC
sgTYMS #1
IB: TYMS

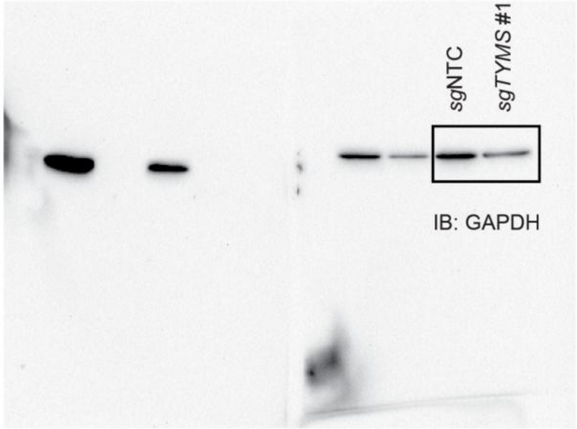
sgNTC
sgTYMS #1
IB: GAPDH

Zimmerman_SuppFig10H immunoblot

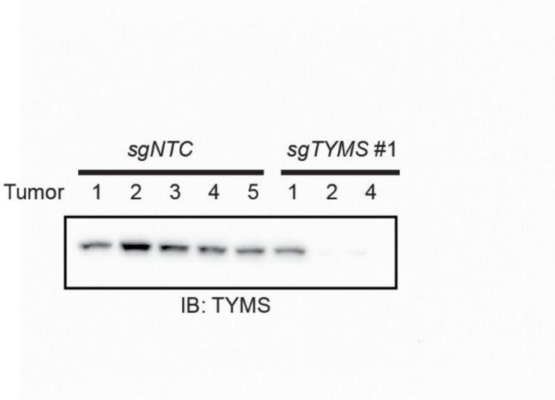

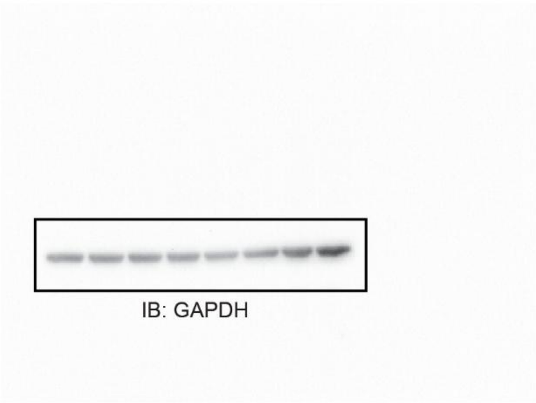

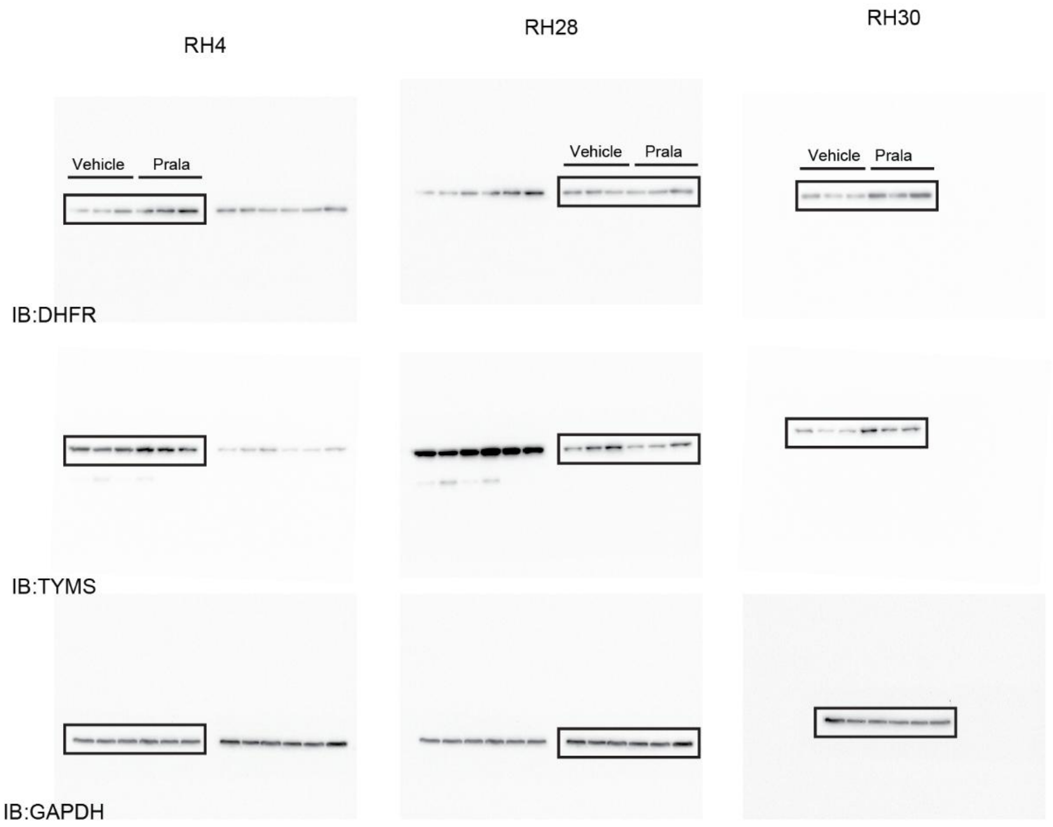
RH4
RH28
RH30
Vehicle
Prala
Vehicle
Prala
Vehicle
Prala
IB:DHFR
IB:TYMS
IB:GAPDH
