# Supplementary material for: Fusion-positive rhabdomyosarcoma oncofusions share a common interactome
Source: Nat Commun. 2026 May 28;17:6933. doi: 10.1038/s41467-026-73749-y (PMC13389464; doi:10.1038/s41467-026-73749-y)
Supplement: Supplementary file 2 — Description of Additional Supplementary Files [file 41467_2026_73749_MOESM2_ESM.pdf]

Description of Additional Supplementary Files

Supplementary Dataset 1: Normalized Peptide Counts for PAX3-FOXO1 interactions.xlsx

Supplementary Dataset 2: Fusion Amino Acid Seq.xlsx

Supplementary Dataset 3: Normalized Counts of Fusion Rescue Transcriptomics.xlsx

Supplementary Dataset 4: C2 GSEA.xlsx

Supplementary Dataset 5: Hallmark GSEA.xlsx

Supplementary Dataset 6: Normalized Peptide Count For Oncofusion Interactions.xlsx

Supplementary Dataset 7: FOXO1 Normalized Peptide Counts.xlsx

Supplementary Dataset 8: Normalized Peptide Counts for Ewings Fusion Interactions.xlsx

Supplementary Dataset 9: Normalized Counts for Tumoroid Transcriptomics.xlsx

Supplementary Dataset 10: Interactome sgRNA Library.xlsx

Supplementary Dataset 11: MAGECK-MLE CRISPR Interactome Screen.xlsx

Supplementary Dataset 12: Transcriptomics Interactome KO.xlsx

Supplementary Dataset 13: Dose Response Analysis.xlsx

Supplementary Dataset 14: Kinome Screen Results.xlsx

Supplementary Dataset 15: sgRNA Oligos.xlsx

Supplementary Dataset 16: Interactome sgRNA Barcodes.xlsx
