# Supplementary material for: Fusion-positive rhabdomyosarcoma oncofusions share a common interactome
Source: Nat Commun. 2026 May 28;17:6933. doi: 10.1038/s41467-026-73749-y (PMC13389464; doi:10.1038/s41467-026-73749-y)
Supplement: Supplementary file 19 — Reporting Summary [file 41467_2026_73749_MOESM19_ESM.pdf]

nature portfolio

Corresponding author(s): Christopher M Counter, Corinne M Linardic

Last updated by author(s): 4-1-26

Reporting Summary

Nature Portfolio wishes to improve the reproducibility of the work that we publish. This form provides structure for consistency and transparency in reporting. For further information on Nature Portfolio policies, see our Editorial Policies and the Editorial Policy Checklist.

Statistics

For all statistical analyses, confirm that the following items are present in the figure legend, table legend, main text, or Methods section.

n/a	Confirmed	
☐	☒	The exact sample size (*n*) for each experimental group/condition, given as a discrete number and unit of measurement
☐	☒	A statement on whether measurements were taken from distinct samples or whether the same sample was measured repeatedly
☐	☒	The statistical test(s) used AND whether they are one- or two-sided<br>*Only common tests should be described solely by name; describe more complex techniques in the Methods section.*
☒	☐	A description of all covariates tested
☐	☒	A description of any assumptions or corrections, such as tests of normality and adjustment for multiple comparisons
☐	☒	A full description of the statistical parameters including central tendency (e.g. means) or other basic estimates (e.g. regression coefficient) AND variation (e.g. standard deviation) or associated estimates of uncertainty (e.g. confidence intervals)
☐	☒	For null hypothesis testing, the test statistic (e.g. *F*, *t*, *r*) with confidence intervals, effect sizes, degrees of freedom and *P* value noted<br>*Give P values as exact values whenever suitable.*
☒	☐	For Bayesian analysis, information on the choice of priors and Markov chain Monte Carlo settings
☒	☐	For hierarchical and complex designs, identification of the appropriate level for tests and full reporting of outcomes
☐	☒	Estimates of effect sizes (e.g. Cohen's *d*, Pearson's *r*), indicating how they were calculated

Our web collection on statistics for biologists contains articles on many of the points above.

Software and code

Policy information about availability of computer code

Data collection	*Provide a description of all commercial, open source and custom code used to collect the data in this study, specifying the version used OR state that no software was used.*
Data analysis	Proteomic Analysis: Following UPLC-MS/MS analyses, data were imported into Proteome Discoverer 2.5 (Thermo Fischer Scientific). In addition to quantitative signal extraction, the MS/MS data was searched against the SwissProt H. sapiens database (downloaded in Nov 2019) and a common contaminant/spiked protein database (bovine albumin, bovine casein, yeast ADH, etc.), and an equal number of reversed-sequence “decoys” for false discovery rate determination. Sequest (v 2.5, Thermo PD) was utilized to produce fragment ion spectra and to perform the database searches. Database search parameters included fixed modification on Cys (carbamidomethyl) and variable modification on DPMSR Fusion Protein Rescue Met (oxidation)<br>mRNA sequencing of cell culture samples: RNA-seq data was processed using the fastp toolkit76 to trim low-quality bases and sequencing adapters from the 3’ end of reads, then mapped to GRCh38 (downloaded from Ensembl, version 106)77 using the STAR RNA-seq alignment tool78, and reads aligning to a single genomic location were summarized across genes. For genes having an overlap of at least 10 reads, gene counts were normalized and differential expression was carried out using the DESeq279 Bioconductor80 package implemented for the R programming environment (R 4.3.0). Consistent with the recommendation of the DESeq authors, independent filtering81 was utilized prior to calculating adjusted p-values and moderated log2 fold-changes were derived using the ashr package82.<br>CUT&Tag<br>After sequencing, Fastq files (trim_galore) were mapped to the reference genome (hg38) using bowtie2 (v.2.4.4). The non-primary alignment and PCR duplicates were removed from aligned data using Samtools (v.1.10) (-q 30 -F 1804 -f 2 for unique mapping reads), the Picard ‘MarkDuplicates’ function (v.2.18.2), and bedtools (v.2.30.0) (‘intersect’ function used to exclude genome blacklist regions), respectively. Peak calling was performed using MACS2 (v.2.2.6) (macs2 callpeak -f BAMPE -g hs/mm –keep-dup 1 --cutoff-analysis -q 0.05). For spike-in samples, fastq files were mapped to the reference genome using STAR (v.2.7.11a). Non-primary alignment and PCR duplicates alignments

were removed by Samtools (v.1.10) (-F 256) and Picard 'MarkDuplicates' function (v.2.18.2). The scale factor used for normalization could be calculated by comparing spike-in alignments number among different groups. Motif analysis was performed by Hypergeometric Optimization of Motif Enrichment (HOMER)41. Deeptools (v3.3.0) was used to generate bigwig files, heatmaps and averaged plotting of CUT&RUN signals42. Genomic binding profiles were generated using the deepTools 'bamCompare' functions. The Genomic Regions Enrichment of Annotations Tool (GREAT)84 was used to determine enriched gene functions (McClean et al., 2010) and Integrative Genomics Viewer (IGV) was used to display and input normalized CUT&Tag signals in bigwig format.

Bioinformatics
Principal Component Analysis (PCA): PCA as carried out in R programming environment (R v4.3.0) in RStudio (v2023.03.0 Build 386) using the stats package (v3.6.2). Data were plotted using the ggplot package (v3.4.4).

Cytoscape: Protein-Protein interaction networks were created using Cytoscape (v3.10.0)124 and stringApp (v2.1.0)50. A list on Uniprot accession numbers meeting the enrichment standards were input into stringApp. Clusters were generated using Markov clustering within stringApp using a granularity parameter of 4.

Gene Set Enrichment (GSE) analysis: GSE analysis125 was performed on the normalized counts output by DEseq2 using the GSEA (4.3.2) software package. The h.all.v2023.Hs.symbols.gmt and c2.all.v2023.1.hs.symbols.gmt gene sets from the Molecular Signatures Database were used for the analysis. Gene symbols were collapsed, permutations were performed on gene_set rather than phenotype and the chip platform selected was the Human_Ensembl_Gene_ID_MSigDB. v2023.1.Hs.chip. The Signal2Noise metric was used to rank genes for data generated from cell culture samples. The Diff_of_classes metric was used to rank genes for data generated from tumoroid models as only two replicates were performed.

STAR Fusion analysis: Raw sequencing data from control and PAX3::WWTR1 knockdown models were trimmed to remove adapters and keep high quality reads by using trim galore v0.6.10. Thereafter, STAR (v2.7.8a) was used to align read counts against the human genome (Gencode v. 37) and STARFusion (v1.10.0) was used to identify candidate fusion transcripts, also against the aforementioned version of the human genome. The function featureCounts from the subread package (v2.0.6) was used to extract raw read counts from the aligned files.

Differential expression and pathway analysis: Raw read counts were loaded into R (v4.2.1). Differential analysis was performed using DESeq2 (v1.36.0) by using condition (i.e., control or fusion knockdown) as contrast. Limma (v3.52.2) was used to correct for potential batch effects from the technical replicates. Pathway analyses was performed on differentially expressed genes using the package clusterProfiler (v4.4.4).

Gene Ontology enrichment analysis: Gene ontology enrichment analysis was performed using the g:Profiler2126 web portal (version e113_eg59_p19_f6a03c19, database updated on 23/05/2025). To reduce redundancy, terms were filtered by algorithmically highlighted annotations that include between 5 and 500 genes.

Dose response curve fitting: Dose response curves were fit to normailzed CellTiter-Glo luminescence readings with the dose log10 transformed in GraphPad Prism (v10.1.2) using the formula: Y=Bottom + (Top-Bottom)/(1+10^((X-LogIC50))). Top value was constrained to 100.

Zip synergy score calculation: Zip synergy scores were calculated using the web based SynergyFinder+ tool (07.09.2024-R-3.10.3)80.

Statistical tests: Curve fitting and statistical tests for cell growth analysis, tumor growth, proximity ligation, Immunoblot densitometry, IC50 calculations, and survival were performed using GraphPad Prism (v10.1.2). All other statistical tests were performed using R (v4.5.0).

MAGeCK CRISPR Screen Analysis: Screen analysis was performed using MAGECK-RRA and MLE127 for the kinome and interactome screen respectively to identify genes selectively enriched or depleted compared to the control.

Normalized Enrichment Score (NES) permutation test: Spearman correlation coefficients for GSE NES scores were calculated across each data set. NES scores were then randomly permutated 2000 times and each time Spearman coefficients recalculated. The measured coefficient distribution was then compared to the distribution of permutated coefficients and p-values calculated.

For manuscripts utilizing custom algorithms or software that are central to the research but not yet described in published literature, software must be made available to editors and reviewers. We strongly encourage code deposition in a community repository (e.g. GitHub). See the Nature Portfolio guidelines for submitting code & software for further information.

Data

Policy information about availability of data

All manuscripts must include a data availability statement. This statement should provide the following information, where applicable:

- Accession codes, unique identifiers, or web links for publicly available datasets
- A description of any restrictions on data availability
- For clinical datasets or third party data, please ensure that the statement adheres to our policy

DATA AVAILABILITY
The mass spectrometry proteomics data have been deposited to the ProteomeXchange Consortium via the PRIDE124 partner repository with the dataset identifiers PXD047629, PXD047630, PXD051746, and PXD071912.

RNA sequencing data pertaining to the RH4-flag cells rescued with oncofusion-TurboV5 have been deposited to the GEO repository with the series record accession number GSE250125.

RNA sequencing data pertaining to the RH4-flag cells targeted with individual sgRNA have been deposited to the GEO repository with the series record accession

number GSE314949.

CRISPR Sequencing Data pertaining to interactome screen have been deposited to the GEO repository with the series record accession number GSE314907.

CRISPR Sequencing Data pertaining to Kinome screen have been deposited to the GEO repository with the series record accession number GSE314862.

CUT&Tag DNA sequencing data have been deposited to the GEO repository with the series record accession number GSE285855.

RNA sequencing data pertaining to the RMS000EEC tumoroids have been deposited to ArrayExpress with the accession number E-MTAB-13715.

Normalized peptide count data was obtained from Ref. 32 for comparison of protein enrichment.

ChimerSeq92 and FusionGDB293–95 datasets were accessed through the relevant web portal on 12-4-2023. FOdb-II dataset was obtained from Ref. 96.

DepMap Public 222Q2. CRISPR_Gene_Effect.csv and Sample_info.csv was accessed through the relevant web portal.

Source Data are provided with this paper.

Research involving human participants, their data, or biological material

Policy information about studies with human participants or human data. See also policy information about sex, gender (identity/presentation), and sexual orientation and race, ethnicity and racism.

Reporting on sex and gender	n/a
Reporting on race, ethnicity, or other socially relevant groupings	n/a
Population characteristics	n/a
Recruitment	n/a
Ethics oversight	This research complies with all relevant ethical regulations, including informed patient consent. In relation to rhabdomyosarcoma patient-derived xenograft SJRHB03117_X, excess, de-identified tumour material was collected from patients with solid tumours at St. Jude Children's Research Hospital in agreement with local institutional ethical regulations and institutional review board approval. Patient consent for tissue acquisition was obtained under the guidelines of the MAST protocol (Title: Molecular Analysis of Solid Tumors; St Jude's IRB Number: Pro00001240; Mnemonic: XPD09-234 MAST (NCT01050296); IRB Approval Date: 3/15/22).<br>RMS000EEC tumoroids and patient tumor samples were obtained via an established tumor sample acquisition route from patients treated at the Emma Children's Hospital Amsterdam (Amsterdam UMC; RMS006, RMS007, RMS013) or as part of the biobank initiative of the Princess Maxima Center for Pediatric Oncology, Utrecht, the Netherlands (PMC; remaining tumor samples). Ethics approval was granted for the biobanking initiative, and the PMC biobank committee granted approval for this project (PMCLAB2018-009). All patients and/or their legal representatives signed informed consent to have tumor samples taken for biobank usage. Experiments conformed to the principles set out in the WMA Declaration of Helsinki and the Department of Health and Human Services Belmont Report.

Note that full information on the approval of the study protocol must also be provided in the manuscript.

Field-specific reporting

Please select the one below that is the best fit for your research. If you are not sure, read the appropriate sections before making your selection.

☒ Life sciences ☐ Behavioural & social sciences ☐ Ecological, evolutionary & environmental sciences

For a reference copy of the document with all sections, see nature.com/documents/nr-reporting-summary-flat.pdf

Life sciences study design

All studies must disclose on these points even when the disclosure is negative.

Sample size	Sample sizes were selected based on prior experience and field standards (at least three replicates), and were sufficient to detect reproducible, biologically meaningful differences; no formal calculation was performed
Data exclusions	No data were excluded
Replication	Outside of immunoblots, Experiments contained technical and biological replicates or were validated by other experimental means. CO-IP of oncofusion protein with TYPS and some other common interactions partnes were attempted and the interaction could not bed detected. This may be do to transient or indeirect interactions between the two proteins. However, the proximity labeling was validated by proximity ligation assays.

	Covariate control was not applicable to this study. All experiments were performed in established cancer cell lines under standardized in vitro conditions, eliminating patient-level or demographic covariates. Technical variation in proteomics datasets was minimized through normalization during data processing; differential abundance analysis accounted for replicate-level variation across independent biological replicates. RNA-seq data were processed using DESeq2 with size factor normalization. No animal studies or clinical cohorts were included that would require covariate adjustment.
Randomization	Animals were randomized for treatment administration by calculating tumor volume at the time of initial treatment and evenly distributing the animals across cohorts based on size.
Blinding	Group allocation was blinded for all animal studies. For in vitro studies, blinding was not relevant, as all statistical analyses were based on quantitative, instrument-generated data (Proteomics, Sequencing, CrispR Screens) acquired using predefined settings and analyzed with standardized pipelines.

Reporting for specific materials, systems and methods

We require information from authors about some types of materials, experimental systems and methods used in many studies. Here, indicate whether each material, system or method listed is relevant to your study. If you are not sure if a list item applies to your research, read the appropriate section before selecting a response.

Materials & experimental systems

n/a	Involved in the study	
☐	☒	Antibodies
☐	☒	Eukaryotic cell lines
☒	☐	Palaeontology and archaeology
☐	☒	Animals and other organisms
☒	☐	Clinical data
☒	☐	Dual use research of concern
☒	☐	Plants

Methods

n/a	Involved in the study	
☒	☐	ChIP-seq
☒	☐	Flow cytometry
☒	☐	MRI-based neuroimaging

Antibodies

Antibodies used	Coverslips were incubated with the V5 (Thermo Fischer Scientific; 1:500) primary antibody diluted in 5% BSA in PBS and incubated at 4 °C for 16 hours. Coverslips were then washed three times in PBS and incubated in alexafluor-555 conjugated goat anti-mouse antibody (Thermo Fischer Scientific, 1:500), alexafluor-488 conjugated streptavidin (Thermo Fischer Scientific, 1:1,000), and 4′,6-diamidino-2-phenylindole (DAPI) (Millipore Sigma; 1:1,000) diluted in 5% BSA in PBS for 1 hour at room temperature.<br><br>Immunoblots were blocked in 5% milk in Tris-buffered saline with Tween (TBST: 20 mM Tris, 150 mM NaCl, and 0.1% (v/v) Tween-20 detergent), or for Streptavidin probing, blocked in 5% BSA (MilliporeSigma) in TBST, followed by immunoblot with the following antibodies in 5% milk in TBST or 5% BSA in TBST: V5 (Thermo Fisher Scientific, R960-25, 1:1,000), flag (Millipore Sigma, f1804, 1:1000), FOXO1 (Cell Signaling, 2880S, 1:1,000), Streptavidin-HRP (Thermo Fisher Scientific, SA10001, 1:5,000), GAPDH (Santa Cruz, sc-365062, 1:1,000), NCOA1 (Abclonal, A9058, 1:500), NCOA2(Abclonal, A10280, 1:500), INO80D(Millipore Sigma, HPA043976, 1:500), MAML1 (Cell Signaling, 12166, 1:500), TYMS (Cell Signaling, 5449, 1:1,000), DHFR (Cell Signaling, 45710, 1:1,000) and ß-Tubulin (Sigma, T5201, 1:5,000).
Validation	Antibodies were validated by shRNA knockdown or sgRNA knockout in the case of anti-FOXO1, V5, FLAG, and TYMS. The remaining antibodies were previously published. NCOA2 antibody was validated by the manufacturer using gene knock out.<br>https://static.abclonal.com/datasheet/A10280.pdf<br>https://static.abclonal.com/datasheet/A9058.pdf<br>https://www.scbt.com/p/gapdh-antibody-g-9<br>https://www.sigmaaldrich.com/US/en/product/sigma/hpa043976?msockid=2e8fe9941ebd6f5e0b07fc281f366eb3<br>https://www.cellsignal.com/products/primary-antibodies/maml1-d3k7b-rabbit-monoclonal-antibody/12166<br>https://www.cellsignal.com/products/primary-antibodies/dhfr-antibody/45710

Eukaryotic cell lines

Policy information about cell lines and Sex and Gender in Research

Cell line source(s)	RH4-flag cells were previously published in "Laubscher, D. et al. BAF complexes drive proliferation and block myogenic differentiation in fusion-positive rhabdomyosarcoma. Nat Commun 12, 6924 (2021)" and provided by one of the senior authors of this study, Dr. Shafter.<br>RMS000EEC tumoroids were originally isolated in "Meister, M. T. et al. Mesenchymal tumor organoid models recapitulate rhabdomyosarcoma subtypes. EMBO Mol Med 14, 1–23 (2022)."
Authentication	RH4, RH28, RH30, and CW9019 cells were STTR profiled and validated.<br>Endogenous oncofusion and P3F1-flag expression was validated by immunoblot analysis with anti-flag or anti-FOXO1 antibodies and/or RT-PCR for RH4, RH4-flag, RH28, and RH30 cells. PAX3::WWTR1 expression was validated by immunoblot analysis from RMS000EEC tumoroid lysates with anti-WWTR1 antibody.

Mycoplasma contamination	Cell lines are tested monthly for mycoplama and consistently tested negative.
Commonly misidentified lines (See ICLAC register)	*Name any commonly misidentified cell lines used in the study and provide a rationale for their use.*

Animals and other research organisms

Policy information about studies involving animals; ARRIVE guidelines recommended for reporting animal research, and Sex and Gender in Research

Laboratory animals	6-8 week old Fox Chase SCID/beige mice (CB17.Cg-PrkdcscidLystbg-J/Crl, Charles River) were used in all studies.
Wild animals	*Provide details on animals observed in or captured in the field; report species and age where possible. Describe how animals were caught and transported and what happened to captive animals after the study (if killed, explain why and describe method; if released, say where and when) OR state that the study did not involve wild animals.*
Reporting on sex	Only female mice were used for all experiments except PDX model reported in this manuscript. Limited sample size prohibited the determination of sex based differences in PDX models. Therefore, sex differences were therefore not considered.
Field-collected samples	*For laboratory work with field-collected samples, describe all relevant parameters such as housing, maintenance, temperature, photoperiod and end-of-experiment protocol OR state that the study did not involve samples collected from the field.*
Ethics oversight	All mouse care and experiments were performed in accordance with a protocol approved by the Institutional Animal Care and Use Committee (IACUC) of Duke University (protocol no. A143-22-08).

Note that full information on the approval of the study protocol must also be provided in the manuscript.

Plants

Seed stocks	*Report on the source of all seed stocks or other plant material used. If applicable, state the seed stock centre and catalogue number. If plant specimens were collected from the field, describe the collection location, date and sampling procedures.*
Novel plant genotypes	*Describe the methods by which all novel plant genotypes were produced. This includes those generated by transgenic approaches, gene editing, chemical/radiation-based mutagenesis and hybridization. For transgenic lines, describe the transformation method, the number of independent lines analyzed and the generation upon which experiments were performed. For gene-edited lines, describe the editor used, the endogenous sequence targeted for editing, the targeting guide RNA sequence (if applicable) and how the editor was applied.*
Authentication	*Describe any authentication procedures for each seed stock used or novel genotype generated. Describe any experiments used to assess the effect of a mutation and, where applicable, how potential secondary effects (e.g. second site T-DNA insertions, mosiacism, off-target gene editing) were examined.*
