# Supplementary material for: Fusion-positive rhabdomyosarcoma oncofusions share a common interactome
Source: Nat Commun. 2026 May 28;17:6933. doi: 10.1038/s41467-026-73749-y (PMC13389464; doi:10.1038/s41467-026-73749-y)
Supplement: Supplementary file 20 — Transparent Peer Review file [file 41467_2026_73749_MOESM20_ESM.pdf]

nature portfolio

Peer Review File

Fusion-positive rhabdomyosarcoma oncofusions share a common interactome

Corresponding Author: Professor Christopher Counter

This file contains all reviewer reports in order by version, followed by all author rebuttals in order by version.

Attachments originally included by the reviewers as part of their assessment can be found at the end of this file.

Version 1:

Reviewer comments:

Reviewer #1

(Remarks to the Author)
The initial observation of this exciting manuscript is that fusion–positive rhabdomyosarcoma (alveolar rhabdomyosarcoma) is typically driven by fusions involving the DNA-binding domains of the transcription factors PAX3 or PAX7 with a variety of partner genes, resulting in oncofusions with diverse C-terminal protein sequences. The authors demonstrate that, despite the heterogeneity in these C-terminal regions, the oncofusions share a common interactome that drives the pathogenesis of fusion–positive rhabdomyosarcoma.

This observation led to the hypothesis that additional, previously unrecognized members of this shared interactome might also drive rhabdomyosarcoma when fused to an N-terminal PAX3/7 domain. Consistent with this idea, the authors identified a novel oncofusion involving PAX3 and WWTR1, a known component of the shared interactome.

Building on this insight, the authors proposed that the common interactome could serve as a source for identifying previously unknown therapeutic vulnerabilities. A CRISPR screen revealed that rhabdomyosarcoma cells depend on the expression of thymidylate synthase, similar to their dependence on PAX3 and FOXO1. Given the essential role of thymidylate synthase in folate metabolism, the authors investigated whether rhabdomyosarcoma cells are sensitive to antifolate agents. They found that pralatrexate inhibits cell growth and acts synergistically with a CDK12 inhibitor, suggesting that this combination may represent a promising therapeutic strategy for this high-risk malignancy.

The findings presented in this manuscript are innovative and supported by high-quality data.

Suggestions:

The translational impact of this manuscript would be significantly enhanced by demonstrating the synergistic effect of pralatrexate and the CDK12 inhibitor in PDX (patient-derived xenograft) mouse models.

As the study exclusively focuses on alveolar/fusion-positive rhabdomyosarcoma, this specificity should be clearly reflected in the manuscript title.

Reviewer #2

(Remarks to the Author)
Zimmerman et al. in the present manuscript “Rhabdomyosarcoma oncofusions share a common interactome” investigate six rare gene fusions found in alveolar rhabdomyosarcoma generated by the PAX3 DNA binding domain fused to C-terminal domains of proteins other than FOXO1. The authors report that these oncofusions behave as PAX3-FOXO1 (P3F1) and P7F1 in vitro supporting cell growth when expressed in the P3F1 RH4 cell line after silencing of the endogenous P3F1. The authors also show that all the seven fusions share a similar interactome, which is specific for the rhabdo-fusions since they used as negative control the EWSR-FLI1 of Ewing sarcoma to eliminate common interactors, induce a common core of genes and bind a common core of genes related to the tumorigenesis of P3F1 rhabdo.
Among common interactors are the full-length C-terminal partners participating in the 6 fusions or their paralogs such as

NCOA1/2, MAML1, INO80D, which were found to bind the same loci than P3F1. Thus the authors suggest that they could be co-activators that serve for the recruitment of the oncofusions.
They also validate a novel fusion PAX3-WWTR1 in an organoid model of rhabdomyosarcoma showing similar gene profiling than RH4 P3F1 cells.
Then, the authors point to one specific interactor, TYMS i.e., the enzyme Thymidylate Synthase, based on Depmap vulnerability data of the 410 common interactors comparing data of P3F1 (and one P7F1) cell lines to all the other tumor cell lines (1079 non-rhabdo cells), to validate their model. After a proximity ligation assay to verify TYMS-P3F1 interaction, they apply TYMS KO to show that three P3F1 cell lines reduce cell proliferation after TYMS depletion.
They then use a folate inhibitor in clinical trial, Pralatrexate, showing that it has the higher effect on the three fusion positive cell lines compared to other folate inhibitors slowering tumor growth in vivo in short term exps. Since the drug-treated tumors recur after the termination of the drug, the authors did a CRISPR KO screen for about 750 kinases identifying a number of kinases that could be targeted to enhance the response to Pralatrexate by synergy, identifying CDK12. In vitro assays effectively showed that the combination of Pralatrexate and a CDK12i has synergistic effects on the three fusion-positive cells augmenting the response lowering the effective dose. Therefore, the authors conclude saying that the identified common interactors "represent a source of potential therapeutic targets". The main conclusion is that the fusions, thanks to the shared interactome, gene profiling and binding to the chromatin, lead to the same tumorigenic phenotype.

The data reported here are interesting and the data reported is in line with some of the conclusions. However, in my opinion there are some key points crucial to support the interpretation that need to be improved and clarified.

Major:
1. The of the full-length C-terminal partners in the oncogenic activity of fusions needs to be clarified. They need to be modulated to evaluate if they are necessary for the recruitment of the fusions on the chromatin and fusion-dependent cell growth. For those full-length C-terminal proteins for which the binding domain is known, the authors could use CRISPR dCas9. This set of experiments will clarify if they cooperate to the oncogenic activity of the fusions.
2. The validation by IP of the interactors has been done only on C-terminal partners in P3F1 cells. Additional interactors need to be validated, which participate in different clusters, and in different oncofusions.
3. The authors report that "…the interactomes and transcriptomes of different oncofusions are largely distinct. As such, proteins uniquely associating with specific oncofusions may provide fusion-specific therapeutic targets". Thus, specific interactors for each fusion need to be identified through the analyses of the author's data and some of these validated by gene modulation to understand their role in each tumorigenic phenotype.
4. The demonstration of a mechanistic link between the targeted interactor TYMS and the fusion proteins is lacking. Dissecting the mechanistic crosstalk between each fusion and TYMS is important to shed light on the role of this enzyme with respect to the oncofusions specific activity. Indeed, TYMS has been related to tumorigenesis in diverse cancers and to promote the development of sarcomas, among which rhabdomyosarcoma, in a Ink4a/Arf –/– phenotype (Guijarro et al, Oncogene 2023). This suggests that TYMS works in a pro-tumorigenic manner also in fusion-negative rhabdomyosarcoma. This raise the following questions: Is the fusions-dependent signature modified after TYMS depletion? And the binding of the oncofusions to the chromatin? In other words, what is the mechanism by which TYMS promotes proliferation and tumorigenesis in fusion-positive rhabdomyosarcoma?
5. In vivo TYMS pharmacologic inhibition did not have the expected long term results based on in vitro data perhaps because TYMS activity should be continuously inhibited, which is difficult to be done with drugs due to side effects. The authors should test this hypothesis by specific depletion of TYMS in vivo.
6. TYMS has been suggested to work with SHMT1 and DHFR, and among them, DHFR is the only gene among the three clearly downregulated by P3F1 shRNA and rescued by P3F1 expression. As reported in Ref. 73, DHFR degradation by NADKi promotes response to the folate inhibitor MTX. Is DHFR up-regulated in tumors that recur after Pralatrexate? This will open the way to a combination of Pralatrexate with DHFR inhibitors not only in vitro but also in vivo. This will add translational value to the manuscript.

Minor:
• The authors should discuss/clarify that the aRMS in which have been detected novel fusions were previously considered fusion-negative aRMS having the same clinical behavior than fusion-negative eRMS (Ref. n. 3 and Missiaglia et al., JCO 2012). This could suggest these oncofusions do not have the same negative impact on overall survival than P3/7F1.
• The authors should provide a Suppl Table with the aminoacid sequences of all the fusions highlighting the fusion point
• SFig. 2D: no visible difference between the blue color for RH4/293N/293C and the other blue colors
• Methods section: what it means that the experiments have been done in quadruplicate or triplicate? Are they biological or technical replicates?
• TYMS has mostly a cytoplasmic and a partial nuclear localization and it is important to understand where the interaction with the fusions is localized: the PLA with V5 and TYMS Abs in Fig.7D seems to show white spots in both nucleus and cytoplasm.
• The interaction with TYMS also needs to be validated in other oncofusions.
• Since "components of the common interactome are also found in other oncofusions", it could be interesting to verify how many of these occupy fragile sites, in order to predict potential new fusions.

Reviewer #3

(Remarks to the Author)
The research is innovative and scientifically significant, exploring how different cancer fusion proteins in RMS share a common interactome. This offers a new perspective on RMS carcinogenesis and holds potential for developing new therapies.

The experimental design is rigorous and uses advanced methods like proximity labeling, mass spectrometry, CRISPR screening, RNA sequencing, and CUT&Tag. These techniques provide a comprehensive analysis of the fusion proteins' interactome, transcriptional regulation, and chromatin binding, ensuring reliable results.
The data is extensive and thoroughly analyzed, revealing that different oncofusions are functionally interchangeable. They interact with a common set of core proteins and drive similar transcriptional programs, strongly supporting the study's conclusions.
The clinical sample analysis and drug screening have translational research value. A new PAX3::WWTR1 fusion was found, and the common interactome predicted this new fusion, showing the study's potential in clinical diagnosis and treatment. Additionally, the anti-folate drug pralatrexate showed significant inhibitory effects on FP-RMS, and a synergistic effect of CDK12 inhibitors with pralatrexate was revealed, providing strong support for future clinical trials.

To enhance the impact of the article, the following optimizations are recommended:

1.Provide more in-depth analysis of the functional significance of the shared interactome. Mechanistic insights into how these interactions specifically drive RMS-related transcriptional programs could further solidify the study's conclusions.

2.Augment the evidence that the common interactome drives oncogenesis. Additional experiments, such as disrupting the interaction between the fusion proteins and the common interactome components to assess the impact on oncogenic phenotypes, would strengthen the conclusions.

3.How do oncogenic fusion proteins lead to similar interactomes? Is it due to the common N-terminus PAX3/PAX7 or various C-terminus? Or is it the shared characteristics after fusion? To clarify this, additional controls such as PAX3/PAX7 N terminous and various C-terminus should be included.

4.In Fig.4B, the control shScrRH4-flag cells expressing TurboV5-NLS produce the same transcriptional signature with the shP3F1 RH4-flag cells rescued with TurboV5-tagged oncofusion proteins. shP3F1 RH4-flag cells should be introduced as a negative control.

Version 2:

Reviewer comments:

Reviewer #1

(Remarks to the Author)
This manuscript reports interesting novel findings indicating common feaures of the interactomes of different fusion proteins in FP-RMS. Specifically, the indentification of TYMS is of particular interest from a translational perspective. The authors have identified folate antagonsm as a potential therapeutic strategy in these tumors and new data show in mice that combination of pralatrexate with vinorelbie is synergistic in mice. These new data have improved the translational outlook of this paper.

Reviewer #2

(Remarks to the Author)
The authors have thoroughly and convincingly addressed all of the reviewers’ comments.
The revised manuscript is substantially strengthened by a large body of new experimental data, including extensive in vivo validation, functional genetic screens, and mechanistic analyses.

The responses are clear, scientifically rigorous, and demonstrate a commendable effort to improve both the depth and translational relevance of the study.

I have no further major or minor concerns and recommend acceptance of the manuscript in its current form.

Reviewer #3

(Remarks to the Author)
The authors have thoroughly addressed all of my previous concerns through additional experiments and clarifications in the revised manuscript. The new data substantially strengthen the conclusions, and the manuscript is now suitable for publication. I recommend acceptance of this work in Nature Communications.

Reviewer #4

(Remarks to the Author)
In the submitted manuscript, Zimmerman et al. examine the mechanistic action of several oncofusions found in fusion-positive rhabdomyosarcoma. I was asked to assist with the review specifically of the bioinformatic analysis of the omics and CUT&Tag within the manuscript.

Overall, the bioinformatic analysis look well-justified and well performed. The methods description is detailed and straightforward (and pretty standard) and the data are presented well. The CUT&Tag bigwig tracks from GEO look like high-quality tracks indicative of a high-quality CUT&Tag run, and the RNA quality data and expression data look good.

In my reading of the rest of the paper, the paradigm is interesting, the data are presented well, and the conclusions are interesting and justified. I think this will be a highly relevant and interesting report with strong implications for the field.

Open Access This Peer Review File is licensed under a Creative Commons Attribution 4.0 International License, which permits use, sharing, adaptation, distribution and reproduction in any medium or format, as long as you give appropriate credit to the original author(s) and the source, provide a link to the Creative Commons license, and indicate if changes were made.
In cases where reviewers are anonymous, credit should be given to 'Anonymous Referee' and the source.
The images or other third party material in this Peer Review File are included in the article's Creative Commons license, unless indicated otherwise in a credit line to the material. If material is not included in the article's Creative Commons license and your intended use is not permitted by statutory regulation or exceeds the permitted use, you will need to obtain permission directly from the copyright holder.
To view a copy of this license, visit https://creativecommons.org/licenses/by/4.0/

<u>Editor comments</u>

<u>Editor summary for Reviewer 1:</u> *"demonstrate the synergistic effect of pralatrexate and the CDK12 inhibitor in PDX mouse models"*

As requested, we provide ***<u>new data</u>*** in which we established a PDX model in our laboratory, no small feat, and tested and found that pralatrexate actually induced *<u>tumor regression</u>*. Unfortunately however, THZ531 is a tool compound, and the dosing described in one published study proved to be rather toxic in our experiments. Thus, while the combination with CDK12 inhibitors is promising, THZ531 will require further development for in vivo analysis. Nevertheless, to address the reviewer's comment to evaluate pralatrexate combinations, we provide ***<u>new data</u>*** that pralatrexate in combination with the second line chemotherapy, vinorelbine (as this is the likely clinical setting for a pralatrexate combination therapy) inhibited tumorigenesis better than either drug alone *<u>and</u>* reduced cumulative toxicity of vinorelbine. These new data establish a relatively quick path for pralatrexate to the clinic.

<u>Editor summary for Reviewer 2</u>: *"clarifying the requirement of the full-length C-terminal partners for the oncogenic activity of the fusions…"*

As requested, we provide ***<u>new data</u>*** in which we explicitly knocked out the individual C-terminal fusion partners in RH4-flag cells with two independent sgRNAs each, all of which inhibited proliferation. As a key control, we then *<u>repeated</u>* this analysis in in the Ewing's sarcoma A673 cells, which are driven by the EWSR1::FLI oncofusion. We now show that the same sgRNAs had no effect in these cells. Thus, the individual C-terminal fusion partners are *<u>specifically</u>* required for FP-RMS oncogenesis. Mechanistically, we provide ***<u>new data</u>*** that inactivating these individual C-terminal fusion partners led to similar changes to the transcriptome as observed upon inactivating the oncofusion itself. Thus, these partners are required for oncofusion activity.

"…validating additional interactors…"

As requested, pilot co-IPs turned out to be a very expensive hit-and-miss approach as we ordered and tried new antibodies. Not surprising as interactions with proteins characterized with intrinsically disordered regions are notoriously difficult to capture, as they typically are weak and transient. Thus, we took another approach, namely we focused on validating the *<u>functional</u>* significance of these interactions. To this end, we provide ***<u>new data</u>*** in which we targeted 13 selected interactome genes with two independent sgRNAs and determining the consequence on growth of RH4-flag cells versus A673 cells, which revealed that all but one sgRNA reduced growth of the RH4-flag cell line, all the while having no effect in the A673 cell line. Furthermore, we provide ***<u>new data</u>*** in which inactivating these genes by sgRNA yields similar changes to the transcriptome as observed upon inactivating the endogenous P3F1 oncofusion. Collectively, these experiments show that the tested interactome proteins are *<u>specifically</u>* required for the oncofusion to drive RMS oncogenesis. To extend this to the *<u>entire</u>* interactome, we provide ***<u>new data</u>*** in which we repeated this analysis, but with an sgRNA library targeting all the genes encoding proteins specifically biotinylated by any TurboV5-oncofusion, as described below. This is in addition to previously demonstrating that common interactome proteins were all proximity labeled by each of the seven separate oncofusions, and that the common interactome components INO80D, MAML1, and NCOA1 were both pulled down in cells expressing P3F1-TurboV5 and co-habitat chromatin sites with P3F1 by CUT&Tag analysis.

"…identifying specific interactors for each fusion…"

As requested, we provide ***new data*** in which we took the huge step of generating an sgRNA library against all proteins specifically biotinylated by any TurboV5-oncofusion. We then screened this library in RH4-flag cells in which the endogenous *P3F1* gene was knocked down and rescued with each of the different types of FP-RMS oncofusions, namely P3F1 (representative of P3F1, P7F1, P3F4), P3N1 (representative of P3N1 and P3N2), P3M1, and P3I. As predicted by the reviewer, this did indeed identify dependencies tracking with activities of specific oncofusions (*i.e.,* acetylation-specific dependencies tracked with P3N1, which contains a C-terminal acetyltransferase domain) or oncofusion-specific interactions that were also oncofusion-specific dependencies (*e.g.,* the kinase MAP2K7 and E3 ubiquitin ligase LTN1 both interacting with P3N1 and being dependencies for cells driven by P3N1). Nevertheless, the majority of proteins shared between the four interactomes that were dependencies, were dependencies in all four cell lines.

"…establishing a mechanistic link between TYMS and the fusions…"

As requested, we provide ***new data*** in which we knocked out TYMS in RH4-flag cells by an sgRNA followed by RNAseq analysis, which revealed similar changes to the transcriptome that were observed upon knocking down P3F1, arguing that TYMS supports the transcriptional program of the oncofusion.

"…and complementing the TYMS pharmacologic inhibition experiment by depleting TYMS specifically…"

As requested, we provide ***new data*** in which we knocked out TYMS in RH28 cells by an sgRNA and then assayed the growth of the resultant cells as xenograft tumors in five mice. This revealed that a loss of TYMS completely prevented tumor growth in two mice while significantly inhibiting tumors growth in the other three compared to the control tumors. These experiments thereby genetically replicated the findings from pharmacologic inhibition of TYMS.

Editor summary for Reviewer 3: *"provide additional evidence that the common interactome drives oncogenesis"*

As requested, we provide ***new data*** in which demonstrate that *1*) targeting 13 selected interactome genes with two independent sgRNAs inhibited the growth of all but one of the 26 derived RH4-flag cell lines while having no effect in the A673 cell line (*see above*), *2*) the common interactome is enriched with FP-RMS dependencies through DepMap analysis, and finally, *3*) a CRISPR loss-of-function screen targeting all oncofusion interactions in four cell lines driven by different oncofusions revealed that the largest contribution to the identified dependencies is derived from the common interactome (*see above*).

"…more in-depth analysis of the functional significance of the shared interactome."

As requested, we provide ***new data*** in which performed Gene Ontology enrichment analysis on dependencies identified above from the aforementioned CRISPR loss-of-function screen. This revealed that common dependencies are enriched for genes involved in ribosome biogenesis and translation, transcription and chromatin remodeling, and DNA replication, consistent with the oncogenic mechanisms of FP-RMS. In addition, we provide ***new data*** that revealed that a panel of common interactome proteins support FP-RMS cell growth and different subsets of the oncogenic transcriptional program of P3F1 through targeted CRISPR/Cas9 knockout of interactome genes (*see above*).

Reviewer 1 comments

Comment 1: *"The translational impact of this manuscript would be significantly enhanced by demonstrating the synergistic effect of pralatrexate and the CDK12 inhibitor in PDX (patient-derived xenograft) mouse models."*

As requested, we provide ***new data*** in which we established the SJRHB031117_X1 PDX model of FP-RMS in our laboratory, and then tested and found that pralatrexate inhibited growth and even induced regression of these PDX tumors (**Fig. 9J,K**). Unfortunately however, THZ531 is a tool compound, and the dosing described in the only published study (PMC8835885) proved to be rather toxic in our hands (not shown), precluding further analysis. Thus, while the combination with CDK12 inhibitors is promising, THZ531 will require further development for preclinical analysis. Nevertheless, to address what we believe to be the essence of this comment – the potential clinical utility of a pralatrexate combination therapy – we provide ***new data*** that pralatrexate in combination with second line chemotherapy vinorelbine (as this is the likely clinical setting for a pralatrexate combination therapy) inhibited tumorigenesis better than either drug alone *and* reduced cumulative toxicity of vinorelbine (**Fig. 10F,G**). These new data point toward a relatively quick path for pralatrexate to the clinic.

Comment 2. *"As the study exclusively focuses on alveolar/fusion-positive rhabdomyosarcoma, this specificity should be clearly reflected in the manuscript title."*

As requested, we changed the title to "Fusion-positive rhabdomyosarcoma oncofusions share a common interactome"

Reviewer 2 comments

Major comment 1. *"The role of the full-length C-terminal partners in the oncogenic activity of fusions needs to be clarified. They need to be modulated to evaluate if they are necessary for the recruitment of the fusions on the chromatin and fusion-dependent cell growth. For those full-length C-terminal proteins for which the binding domain is known, the authors could use CRISPR dCas9. This set of experiments will clarify if they cooperate to the oncogenic activity of the fusions."*

As requested, we provide ***new data*** in which we explicitly tested this point by individually knocking out the genes encoding the full-length C-terminal fusion partners (*FOXO1, NCOA1, NCOA2, INO80D,* and *MAML1*) in RH4-flag cells with two independent sgRNAs each, all of which inhibited proliferation compared to a non-targeting control sgRNA (**Fig. 7A**). We then repeated this entire analysis in in the Ewing's sarcoma A673 cell line, which is also a pediatric sarcoma driven by a transcriptional oncofusion, although in this case, the oncofusion is EWSR1::FLI. None of the sgRNAs had any effect on proliferation (**Fig. 7A**). Thus, the individual C-terminal fusion partners are *specifically* required for RMS oncogenesis. We next performed RNAseq on these cells, and provide ***new data*** that loss of these full-length C-terminal fusion partners resulted in a subset of the transcriptional changes observed upon targeting the P3F1 oncofusion itself, including transcriptional targets and myogenic and proliferative gene sets (**Fig. 7B-D**). To independently validate these results, we took advantage of the new oncofusion we described in this study, PAX3::WWTR1 (**Fig. 5**). Namely, we repeated both experiments, but targeted WWTR1, which behaved in a similar manner to the other six partners (**Fig. 7A-D**). Unfortunately, CUT&Tag analysis proved difficult, as loss of these full-length C-terminal fusion partners had a profound effect on cell viability, which in our experience leads to widespread nonspecific changes in chromatin interaction. Nevertheless, as predicted by this reviewer, the loss of full-length C-terminal fusion partners, including the novel partner that was not included in the original proximity labeling experiment, all reduced cell growth and generated transcriptional changes consistent with the loss of the oncofusion itself.

Major comment 2: *"The validation by IP of the interactors has been done only on C-terminal partners in P3F1 cells. Additional interactors need to be validated, which participate in different clusters, and in different oncofusions."*

As requested, pilot co-IPs turned out to be a very expensive hit-and-miss approach as we ordered and tried new antibodies. Not surprising as interactions with proteins characterized with intrinsically disordered regions are typically weak and transient (PMC10666024; PMC6295254; PMC10208215). We also tried to validate these interactions by PLA analysis, however the reagents necessary for this analysis have been on backorder for months. Thus, we took another approach, namely we focused on validating the *functional* significance of these interactions. To this end, we provide ***new data*** in which we targeted 13 selected interactome genes with two independent sgRNAs and determining the consequence on growth of RH4-flag versus A673 cells, which revealed that all but one sgRNA reduced growth of the RH4-flag cell line while having no effect in the A673 cell line (**Fig. 7A**). Furthermore, we provide ***new data*** in which inactivating these genes by sgRNA yielded similar changes to the transcriptome as observed upon inactivating the endogenous P3F1 oncofusion (**Fig. 7B-D**). Collectively, these experiments show that the tested interactome proteins are *specifically* required for the oncofusion to drive FP-RMS oncogenesis. To extend this analysis to the *entire* interactome, we provide ***new data*** in which we constructed a custom library comprised of three independent sgRNAs targeting any gene encoding a protein specifically biotinylated by any of the seven TurboV5-oncofusions. We then

screened this library in RH4-flag cells in which the endogenous *P3F1* gene was knocked down with an *shP3F1* specifically targeting the breakpoint and rescued with each of the different types of FP-RMS oncofusions, namely P3F1 (representative of P3F1, P7F1, P3F4), P3N1 (representative of P3N1 and P3N2), P3M1, and P3I. As predicted by the reviewer, this did indeed identify common interactome proteins that were dependencies (**Fig. 6B,C,E**). Finally, to determine if genes encoding proteins of the common interactome were specifically required for FP-RMS, we provide ***new data*** in which we interrogated DepMap, finding that the common interactome is indeed enriched with FP-RMS dependencies (**Fig. 6A**). Thus, by focusing on the functional consequence of those proteins specifically biotin-labeled by all seven TurboV5-oncofusions, an event that statistically cannot occur by mere chance, we were able to interrogate the entire common interactome, not just a few proteins. This comprehensive and unbiased approach confirmed that a major portion of the common interactome do indeed contribute to the oncogenic activity of the different oncofusions.

Major comment 3: "*The authors report that "…the interactomes and transcriptomes of different oncofusions are largely distinct. As such, proteins uniquely associating with specific oncofusions may provide fusion-specific therapeutic targets". Thus, specific interactors for each fusion need to be identified through the analyses of the author's data and some of these validated by gene modulation to understand their role in each tumorigenic phenotype."*

As requested, we provide the above ***new data*** in which we screened an sgRNA library targeting every protein specifically labeled by any of the seven TurboV5-oncofusions in a panel of FP-RMS cell lines engineered to be driven by P3F1, P3N1, P3M1, or P3I. We then cross-referenced the genes that were negatively enriched in one, two, three, or all four of these cell lines with proteins that were biotin labeled by one, two, three, or all four of these oncofusions. As predicted by the reviewer, this did indeed identify dependencies tracking with activities of specific oncofusions (*i.e.,* acetylation-specific dependencies tracked with P3N1, which contains a C-terminal acetyltransferase domain) or oncofusion-specific interactions that were also oncofusion-specific dependencies (*e.g.,* the kinase MAP2K7 and E3 ubiquitin ligase LTN1 both interacting with P3N1 and being dependencies for cells driven by P3N1). Nevertheless, the majority of proteins shared between the four interactomes that were dependencies, were dependencies in all four cell lines (**Fig. 6E**). Thank you for this suggestion, as this analysis could serve as the foundation for not only interrogating the differences between the oncofusions, but perhaps even new opportunities to specifically target them.

Major comment 4: *"The demonstration of a mechanistic link between the targeted interactor TYMS and the fusion proteins is lacking. Dissecting the mechanistic crosstalk between each fusion and TYMS is important to shed light on the role of this enzyme with respect to the oncofusions specific activity. Indeed, TYMS has been related to tumorigenesis in diverse cancers and to promote the development of sarcomas, among which rhabdomyosarcoma, in a Ink4a/Arf −/− phenotype (Guijarro et al, Oncogene 2023). This suggests that TYMS works in a pro-tumorigenic manner also in fusion-negative rhabdomyosarcoma. This raise the following questions: Is the fusions-dependent signature modified after TYMS depletion? And the binding of the oncofusions to the chromatin? In other words, what is the mechanism by which TYMS promotes proliferation and tumorigenesis in fusion-positive rhabdomyosarcoma?"*

We thank the reviewer for this suggestion and pointing us to the interesting article linking TYMS to rhabdomyosarcoma. The article is now included in the discussion. ***As requested***, we provide ***new data*** in which we depleted TYMS by sgRNA in RH4-flag cells (**Supp. Fig. 10D**) and assessed the effects on both PAX3::FOXO1 chromatin interactions by CUT&Tag (**Supp. Fig. 10C**)

and transcriptional changes by RNAseq (**Fig. 7B-D**). This revealed that that the loss of TYMS does not significantly alter P3F1 interactions with chromatin, but does indeed alter the transcriptome in a fashion similar to the loss of P3F1. Thus, TYMS appears to mediate transcriptional activity of the oncofusion, not localization to target genes.

Major Comment 5: *"In vivo TYMS pharmacologic inhibition did not have the expected long term results based on in vitro data perhaps because TYMS activity should be continuously inhibited, which is difficult to be done with drugs due to side effects. The authors should test this hypothesis by specific depletion of TYMS in vivo."*

As requested, we provide ***new data*** in which TYMS was stably knocked out in RH28 cells with an *sgTYMS* (**Supp. Fig. 10F**) and implanted the resultant cells injected into the flank of five SCID Beige mice. As a control, the same cells were stably transduced with a non-targeting control sgRNA and similarly implanted into five mice. Tumor growth (**Supp. Fig. 10G**) and mouse survival (**Fig. 8G**) was then compared between the two cohorts. This revealed that a loss of TYMS completely prevented tumor growth in two mice, and in another three, the tumors grew significantly slower compared to the control tumors. Interesting, despite validating TYMS knockout prior to injection (**Supp. Fig. 10F**), one of the latter tumors exhibited of TYMS expression (**Supp. Fig. 10H**), suggestive of a strong selective pressure for TYMS expression. Thus, genetic inactivation of TYMS replicates the antineoplastic effect of pharmacologically inhibiting this enzyme.

Major Comment 6: *"TYMS has been suggested to work with SHMT1 and DHFR, and among them, DHFR is the only gene among the three clearly downregulated by P3F1 shRNA and rescued by P3F1 expression. As reported in Ref. 73, DHFR degradation by NADKi promotes response to the folate inhibitor MTX. Is DHFR up-regulated in tumors that recur after Pralatrexate? This will open the way to a combination of Pralatrexate with DHFR inhibitors not only in vitro but also in vivo. This will add translational value to the manuscript."*

We thank this reviewer for their insight, and as requested, to explore this possibility we determined the levels of DHFR and TYMS protein by immunoblot analysis in six tumor xenografts derived from RH4, RH28, and RH30 cells implanted in mice and treated with either vehicle or pralatrexate. These ***new data*** revealed that both DHFR and TYMS protein levels were significantly upregulated in RH30-derived tumors after pralatrexate treatment, while DHFR levels trended towards being higher in RH4-derived tumors after pralatrexate treatment (**Supp. Fig. 11C,D**). Thus, as suggested by this reviewer, these data indeed point towards upregulation of this pathway as a possible mechanism of resistance in these cells.

Minor comment 1: *"The authors should discuss/clarify that the aRMS in which have been detected novel fusions were previously considered fusion-negative aRMS having the same clinical behavior than fusion-negative eRMS (Ref. n. 3 and Missiaglia et al., JCO 2012). This could suggest these oncofusions do not have the same negative impact on overall survival than P3/7F1."*

As requested, we thank this reviewer for this insight, and address this concept in the revised discussion.

Minor comment 2: *"The authors should provide a Suppl Table with the amino acid sequences of all the fusions highlighting the fusion point"*

As requested, we now include a new **Supp. Table 2** providing the amino acid sequence of the N- and C-terminal fusion portions of each oncofusion.

Minor Comment 3. "*SFig. 2D: no visible difference between the blue color for RH4/293N/293C and the other blue colors."*

As requested, the color scheme of this figure has been changed so that it is easier to determine what proteins belong to what classification (**Supp. Fig. 1D**).

Minor Comment 4: *"Methods section: what it means that the experiments have been done in quadruplicate or triplicate? Are they biological or technical replicates?"*

As requested, we now denote when replicates are biological or technical replicates in the materials and methods as well as the figure legends.

Minor Comment 5: *"TYMS has mostly a cytoplasmic and a partial nuclear localization and it is important to understand where the interaction with the fusions is localized: the PLA with V5 and TYMS Abs in Fig.7D seems to show white spots in both nucleus and cytoplasm."*

As requested, we revised the text to explicitly note the localization.

Minor Comment 6: *"The interaction with TYMS also needs to be validated in other oncofusions."*

We apologize, we have attempted to repeat PLA analysis with other oncofusions but the reagents have been backordered now for months.

Minor Comment 7: *"Since "components of the common interactome are also found in other oncofusions", it could be interesting to verify how many of these occupy fragile sites, in order to predict potential new fusions."*

We thank this reviewer for this extremely intriguing idea, which we put a fair bit of effort into. First, we used the HumCFS data set to identify if any known fusion-partner genes fell within a cytogenetically defined fragile site. We found that most did not. In fact, the most common oncofusion gene partners, *PAX3* and *FOXO1*, did not. This suggested that while oncofusion genes from other cancers are found within fragile sites, it is not necessarily the same in FP-RMS. We then repeated this analysis with the common interactome, which revealed about a quarter of the common interactome genes localized to fragile sites. Second, enriched sites of gamma-H2AX incorporation in chromatin have previously been used as indicators of common break points. Further, these sites become larger in size and more frequent during replication stress. We therefore compared gene loci for known FP-RMS fusion gene partners to sites of significantly enriched gamma-H2AX under untreated or replicative stressed conditions. As observed in the case of fragile sites, most oncofusion partner genes did not contained chromatin enriched with gamma-H2AX under either condition. Similarly, *PAX3* and *FOXO1* genes do not contain gamma-H2AX. Nevertheless, there is clearly some feature of common interactome genes that favors being a fusion partner. Namely, we cross referenced the common interactome with ChimerDB and found that half the common interactome genes reside within translocations from other tumors and a quarter in other transcription factor oncofusions (**Supp. Fig. 13A,B**).

Reviewer 3 comments

Comment 1: *"Provide more in-depth analysis of the functional significance of the shared interactome. Mechanistic insights into how these interactions specifically drive RMS-related transcriptional programs could further solidify the study's conclusions."*

As requested, we provide ***new data*** in which individually knocked out genes encoding 13 components of the common interactome with two independent sgRNAs each in RH4-flag cells with, all of which inhibited proliferation compared to a non-targeting control sgRNA. We then repeated this entire analysis in in the Ewing's sarcoma A673 cells, which are also a pediatric sarcoma that are also driven by a transcriptional oncofusion, although in this case EWSR1::FLI. None of the sgRNAs had any effect on proliferation (**Fig. 7A**). Thus, these tested interactome proteins are *specifically* required for RMS oncogenesis. We next performed RNAseq on nine of these cell lines, which included sgRNAs targeting the all the C-terminal fusion partners, TYMS, and two additional interacting proteins, and provide ***new data*** that loss of these proteins resulted in a subset of the transcriptional changes observed upon targeting the P3F1 oncofusion itself, including transcriptional targets and myogenic and proliferative gene sets (**Fig. 7B-D**). This in addition to the previous analysis demonstrating that three tested interactome proteins that are also C-terminal fusion partners co-localize with P3F1 on chromatin (**Fig. 4H,I**), including known P3F1-target genes (**Fig. 4J**), consistent with these interactions mediating P3F1 oncogenic transcription. However, to more comprehensively interrogate the common interactome across different oncofusions, we provide ***new data*** in which we constructed a custom sgRNA library targeting with three independent sgRNAs any gene encoding a protein specifically biotinylated by any of the seven TurboV5-oncofusions. We then screened this library in RH4-flag cells in which the endogenous *P3F1* gene was knocked down with an *shP3F1* specifically targeting the breakpoint and rescued with each of the different types of FP-RMS oncofusions, namely P3F1 (representative of P3F1, P7F1, P3F4), P3N1 (representative of P3N1 and P3N2), P3M1, and P3I. This revealed the majority of dependencies were associated with proteins of the common interactome, and mapped to GO terms associated with tumorigenesis (**Fig. 6B-F**). Finally, to determine if genes encoding proteins of the common interactome were specifically required for FP-RMS, we provide ***new data*** in which we interrogated DepMap and find that the common interactome is indeed enriched with FP-RMS dependencies (**Fig. 6A**). This comprehensive and unbiased approach confirmed that a major portion of the common interactome does indeed contribute to the oncogenic activity of the different oncofusions.

Comment 2: *"Augment the evidence that the common interactome drives oncogenesis. Additional experiments, such as disrupting the interaction between the fusion proteins and the common interactome components to assess the impact on oncogenic phenotypes, would strengthen the conclusions."*

As requested, as noted above, in conjunction with the ***new data*** from the above CRISPR screen (**Fig. 6**), we also provide ***new data*** demonstrating that in a panel of cell lines in which common interactome proteins were specifically targeted by sgRNA, including TYMS and WWTR1, FP-RMS cell growth and different subsets of the oncogenic transcriptional programs of P3F1 were disrupted (**Fig. 7**).

<u>Comment 3:</u> *"How do oncogenic fusion proteins lead to similar interactomes? Is it due to the common N-terminus PAX3/PAX7 or various C-terminus? Or is it the shared characteristics after fusion? To clarify this, additional controls such as PAX3/PAX7 n-terminus and various C-terminus should be included."*

We thank this reviewer for this excellent insight. Initially we addressed this point by demonstrating that many of the common interactome proteins were lost if we fused the PAX3 DNA-binding domain to the EWSR1 portion of the EWSR1::FLI1 oncofusion, arguing that it was the C-termini imparting much of this specificity. In agreement, the opposite experiment – namely fusing the different C-termini to PAX3 in form of the different oncofusions – recruited the same core set of proteins. To further explore this, we attempted to perform proximity labeling with full-length PAX3, or just the PAX3 N-terminus, fused to TurboV5, but were unable to express these proteins. However, the opposite experiment was more successful. Namely, we were able to express full-length FOXO1 fused to TurboV5 in RH4-flag cells (**Supp. Fig. 2G**). Importantly, we demonstrate that this did *<u>not</u>* rescue the loss of P3F1 (**Supp. Fig. 2H,I**). We now provide further ***<u>new data</u>*** whereby we captured and identified proteins biotinylated by FOXO1-TurboV5, but not control NLS-TurboV5 (**Supp. Table 7**), and demonstrate significant overlap of these proteins with the common interactome (**Supp. Fig. 5A**). Importantly, this overlap included all the full-length C-terminal partners, except INO80D, indicating that the C-terminus of P3F1 is likely driving those interactions (**Supp. Fig. 5A**). Again, we thank the review for suggesting this line of investigation, as it provided independent validation supporting a model whereby the C-termini recruits the common interactome.

<u>Comment 4:</u> *"In Fig.4B, the control shScrRH4-flag cells expressing TurboV5-NLS produce the same transcriptional signature with the shP3F1 RH4-flag cells rescued with TurboV5-tagged oncofusion proteins. shP3F1 RH4-flag cells should be introduced as a negative control."*

As requested, we clarified the text to indicate that each column in this figure (now **Fig. 3B**) represents gene set enrichment analysis of each of the cell lines labeled in comparison to cells expressing *shP3F1* and TurboV5-NLS.

Reviewer 1 comments

Comment: “This manuscript reports interesting novel findings indicating common feaures of the interactomes of different fusion proteins in FP-RMS. Specifically, the indentification of TYMS is of particular interest from a translational perspective. The authors have identified folate antagonsm as a potential therapeutic strategy in these tumors and new data show in mice that combination of pralatrexate with vinorelbie is synergistic in mice. These new data have improved the translational outlook of this paper.”

Reviewer 2 comments

Comment: “The authors have thoroughly and convincingly addressed all of the reviewers’ comments. The revised manuscript is substantially strengthened by a large body of new experimental data, including extensive in vivo validation, functional genetic screens, and mechanistic analyses. The responses are clear, scientifically rigorous, and demonstrate a commendable effort to improve both the depth and translational relevance of the study. I have no further major or minor concerns and recommend acceptance of the manuscript in its current form.”

Reviewer 3 comments

Comment: “The authors have thoroughly addressed all of my previous concerns through additional experiments and clarifications in the revised manuscript. The new data substantially strengthen the conclusions, and the manuscript is now suitable for publication. I recommend acceptance of this work in Nature Communications.”

Reviewer 4 comments

Comment: “In the submitted manuscript, Zimmerman et al. examine the mechanistic action of several oncofusions found in fusion-positive rhabdomyosarcoma. I was asked to assist with the review specifically of the bioinformatic analysis of the omics and CUT&Tag within the manuscript. Overall, the bioinformatic analysis look well-justified and well performed. The methods description is detailed and straightforward (and pretty standard) and the data are presented well. The CUT&Tag bigwig tracks from GEO look like high-quality tracks indicative of a high-quality CUT&Tag run, and the RNA quality data and expression data look good. In my reading of the rest of the paper, the paradigm is interesting, the data are presented well, and the conclusions are interesting and justified. I think this will be a highly relevant and interesting report with strong implications for the field.”

Response

We are grateful to all reviewers for their insightful comments and feedback throughout the review process.

Zimmerman et al. in the present manuscript “Rhabdomyosarcoma oncofusions share a common interactome” investigate six rare gene fusions found in alveolar rhabdomyosarcoma generated by the PAX3 DNA binding domain fused to C-terminal domains of proteins other than FOXO1. The authors report that these oncofusions behave as PAX3-FOXO1 (P3F1) and P7F1 *in vitro* supporting cell growth when expressed in the P3F1 RH4 cell line after silencing of the endogenous P3F1. The authors also show that all the seven fusions share a similar interactome, which is specific for the rhabdo-fusions since they used as negative control the EWSR-FLI1 of Ewing sarcoma to eliminate common interactors, induce a common core of genes and bind a common core of genes related to the tumorigenesis of P3F1 rhabdo.
Among common interactors are the full-length C-terminal partners participating in the 6 fusions or their paralogs such as NCOA1/2, MAML1, INO80D, which were found to bind the same loci than P3F1. Thus the authors suggest that they could be co-activators that serve for the recruitment of the oncofusions.
They also validate a novel fusion PAX3-WWTR1 in an organoid model of rhabdomyosarcoma showing similar gene profiling than RH4 P3F1 cells.
Then, the authors point to one specific interactor, TYMS i.e., the enzyme Thymidylate Synthase, based on Depmap vulnerability data of the 410 common interactors comparing data of P3F1 (and one P7F1) cell lines to all the other tumor cell lines (1079 non-rhabdo cells), to validate their model. After a proximity ligation assay to verify TYMS-P3F1 interaction, they apply TYMS KO to show that three P3F1 cell lines reduce cell proliferation after TYMS depletion.
They then use a folate inhibitor in clinical trial, Pralatrexate, showing that it has the higher effect on the three fusion positive cell lines compared to other folate inhibitors slowering tumor growth *in vivo* in short term exps. Since the drug-treated tumors recur after the termination of the drug, the authors did a CRISPR KO screen for about 750 kinases identifying a number of kinases that could be targeted to enhance the response to Pralatrexate by synergy, identifying CDK12. *In vitro* assays effectively showed that the combination of Pralatrexate and a CDK12i has synergistic effects on the three fusion-positive cells augmenting the response lowering the effective dose. Therefore, the authors conclude saying that the identified common interactors “represent a source of potential therapeutic targets”. The main conclusion is that the fusions, thanks to the shared interactome, gene profiling and binding to the chromatin, lead to the same tumorigenic phenotype.

The data reported here are interesting and the data reported is in line with some of the conclusions. However, in my opinion there are some key points crucial to support the interpretation that need to be improved and clarified.

Major:
1. The of the full-length C-terminal partners in the oncogenic activity of fusions needs to be clarified. They need to be modulated to evaluate if they are necessary for the recruitment of the fusions on the chromatin and fusion-dependent cell growth. For those full-length C-terminal proteins for which the binding domain is known, the authors could use CRISPR dCas9. This set of experiments will clarify if they cooperate to the oncogenic activity of the fusions.
2. The validation by IP of the interactors has been done only on C-terminal partners in P3F1 cells. Additional interactors need to be validated, which participate in different clusters, and in different oncofusions.
3. The authors report that “…the interactomes and transcriptomes of different oncofusions are largely distinct. As such, proteins uniquely associating with specific oncofusions may provide fusion-specific therapeutic targets”. Thus, specific interactors for each fusion need to be identified through the analyses of the author’s data and some of these validated by gene modulation to understand their role in each tumorigenic phenotype.
4. The demonstration of a mechanistic link between the targeted interactor TYMS and the fusion proteins is lacking. Dissecting the mechanistic crosstalk between each fusion and TYMS is

important to shed light on the role of this enzyme with respect to the oncofusions specific activity. Indeed, TYMS has been related to tumorigenesis in diverse cancers and to promote the development of sarcomas, among which rhabdomyosarcoma, in a *Ink4a/Arf −/−* phenotype (Guijarro et al, Oncogene 2023). This suggests that TYMS works in a pro-tumorigenic manner also in fusion-negative rhabdomyosarcoma. This raise the following questions: Is the fusions-dependent signature modified after TYMS depletion? And the binding of the oncofusions to the chromatin? In other words, what is the mechanism by which TYMS promotes proliferation and tumorigenesis in fusion-positive rhabdomyosarcoma?

5. *In vivo* TYMS pharmacologic inhibition did not have the expected long term results based on *in vitro* data perhaps because TYMS activity should be continuously inhibited, which is difficult to be done with drugs due to side effects. The authors should test this hypothesis by specific depletion of TYMS *in vivo*.

6. TYMS has been suggested to work with SHMT1 and DHFR, and among them, DHFR is the only gene among the three clearly downregulated by P3F1 shRNA and rescued by P3F1 expression. As reported in Ref. 73, DHFR degradation by NADKi promotes response to the folate inhibitor MTX. Is DHFR up-regulated in tumors that recur after Pralatrexate? This will open the way to a combination of Pralatrexate with DHFR inhibitors not only *in vitro* but also *in vivo*. This will add translational value to the manuscript.

Minor:

- The authors should discuss/clarify that the aRMS in which have been detected novel fusions were previously considered fusion-negative aRMS having the same clinical behavior than fusion-negative eRMS (Ref. n. 3 and Missiaglia et al., JCO 2012). This could suggest these oncofusions do not have the same negative impact on overall survival than P3/7F1.
- The authors should provide a Suppl Table with the aminoacid sequences of all the fusions highlighting the fusion point
- SFig. 2D: no visible difference between the blue color for RH4/293N/293C and the other blue colors
- Methods section: what it means that the experiments have been done in quadruplicate or triplicate? Are they biological or technical replicates?
- TYMS has mostly a cytoplasmic and a partial nuclear localization and it is important to understand where the interaction with the fusions is localized: the PLA with V5 and TYMS Abs in Fig.7D seems to show white spots in both nucleus and cytoplasm.
- The interaction with TYMS also needs to be validated in other oncofusions.
- Since “components of the common interactome are also found in other oncofusions”, it could be interesting to verify how many of these occupy fragile sites, in order to predict potential new fusions.
